# A multi-omics machine learning classifier for outgrowth of cow's milk allergy in children†

**DOI:** 10.1039/d4mo00245h

**Published:** 2025-05-09

**Authors:** Diana M. Hendrickx, Mariyana V. Savova, Pingping Zhu, Ran An, Sjef Boeren, Kelly Klomp, Sumanth K. Mutte, PRESTO study team, Harm Wopereis, Renate G. van der Molen, Amy C. Harms, Clara Belzer

**Affiliations:** a Laboratory of Microbiology, Wageningen University Wageningen The Netherlands clara.belzer@wur.nl; b Metabolomics and Analytics Centre, Leiden Academic Centre for Drug Research, Leiden University Leiden The Netherlands; c Laboratory of Biochemistry, Wageningen University Wageningen The Netherlands; d Danone Nutricia Research Utrecht The Netherlands; e Department of Laboratory Medicine, Laboratory of Medical Immunology Radboudumc Nijmegen The Netherlands

## Abstract

Cow's milk protein allergy (CMA) is one of the most common food allergies in children worldwide. However, it is still not well understood why certain children outgrow their CMA and others do not. While there is increasing evidence for a link of CMA with the gut microbiome, it is still unclear how the gut microbiome and metabolome interact with the immune system. Integrating data from different omics platforms and clinical data can help to unravel these interactions. In this study, we integrate clinical, microbial, (meta)proteomics, immune and metabolomics data into machine learning (ML) classification, using multi-view learning by late integration. The aim is to group infants into those that outgrew their CMA and those that did not. The results show that integration of microbiome data with clinical, immune, (meta)proteomics and metabolomics data could considerably improve classification of infants on outgrowth of CMA, compared to only considering one type of data. Moreover, pathways previously linked to development of CMA could also be related to outgrowth of this allergy.

## Abstract 2

A multi-omics machine learning classifier was developed to distinguish between infants that outgrew their cow's milk allergy and those who did not.

## Graphical abstract

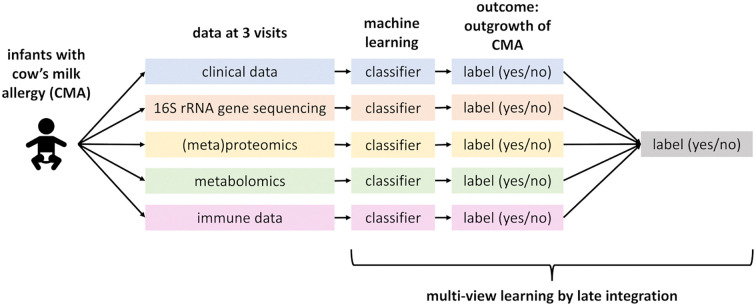

## 1. Introduction

Cow's milk protein allergy (CMA) is a common food allergy in children characterized by abnormal reactions of the immune system to cow's milk (CM) proteins. Two types of reactions can be distinguished: immunoglobulin (Ig)E-mediated reactions which are mostly immediate reactions, and non-IgE-mediated reactions which are mostly delayed.^1^ Some children also have a combination of both IgE-mediated and non-IgE-mediated reactions.^1^ The majority of the children outgrow their CMA in the first years of life, and outgrowth of CMA is in general slower in case the CMA is IgE-mediated compared to non-IgE-mediated.^2^

There is increased evidence for a link between CMA, gut microbiome dysbiosis and altered levels of short chain fatty acids (SCFA).^3,4^

It is currently unclear how the gut microbiome interacts with the immune system. The inclusion of data of the faecal metabolome, the microbial metaproteome and the human proteome could advance our understanding of these interactions.^3^

However, multi-omics studies on CMA including both microbiome and host data are limited and in general have small sample size.^3^

In this study, our primary goal is to improve the understanding of CMA through a multi-omics machine learning approach. Developing an efficient classifier that can deal with small sample size studies is essential for achieving this goal.

We aim to integrate 16S rRNA gene sequencing, (meta)proteomics and metabolomics obtained from stool samples, immune data from saliva samples and clinical data by applying a machine learning (ML) classification approach, using multi-view learning. Multi-view learning considers learning from multiple types of data (= views) from the same subjects to improve the performance on independent data (not used for building the ML model),^5^ also called the generalization performance. A straightforward approach would be combining all data into a single data set and fit one ML classifier to these data. However, this would lead to overfitting the data, lowering the generalization performance.^5^ Other drawbacks of combining all data into a single data set include that the different statistical properties of each separate data set are ignored, and that all data sets need to be complete. To overcome these limitations, multi-view learning by late integration is applied. An ML classifier is fitted for each view, and the predictions of all these classifiers are combined.

In this study, we build a multi-view ML classifier to group infants into two categories: those who outgrew IgE-mediated CMA and those who did not.

## 2. Materials and methods

### 2.1 Sample collection and study design

Stool and saliva samples from a subset of 40 infants 13 months and younger from the PRESTO study (NTR3725),^6^ retrieved from Danone Nutricia Research as described previously,^7^ were used for this study. In summary, the PRESTO study included infants with confirmed diagnosis of IgE-mediated CMA randomized to receive a standard amino acid-based formula (AAF) or an amino acid-based formula supplemented with a synbiotic blend (AAF-syn) (probiotic *Bifidobacterium breve* M-16V and prebiotic oligosaccharides (oligofructose and inulin)) as described elsewhere.^6^ Samples were collected at different study sites according to the same protocol (coordinated by Danone Nutricia Research). Stool samples were collected before the start of the study (baseline visit), and after 6 months (visit 6 M) and 12 months (visit 12 M) of intervention with AAF or AAF-syn, and were analysed by 16S rRNA gene sequencing, (meta)proteomics and metabolomics. Saliva samples were analysed for biomarkers of inflammation and immune response at the same three visits. Each -omics analysis was conducted by a single institution/lab. The 16S rRNA gene amplicon sequencing was conducted at LifeSequencing S. L. (Valencia, Spain), metaproteomics at Wageningen University (The Netherlands), metabolomics at Leiden University (The Netherlands) and immune data at Radboudumc (Nijmegen, The Netherlands). Of the 40 infants used in this study, selected as described previously, 24 outgrew their allergy after 12 months (10 AAF, 14 AAF-syn), while the allergy persisted in 15 infants (6 AAF, 9 AAF-syn). As described previously, one infant was excluded because outgrowth of allergy at 12 months was unknown.

### 2.2 Ethical approval

Ethical approval was obtained as described earlier.^8^ In summary, this multicenter study was performed according to the World Medical Association (WMA) Declaration of Helsinki and the International Conference on Harmonization guidelines for Good Clinical Practice.^8^ The samples for our study were collected at 10 sites in 6 countries (United Kingdom, Germany, Italy, Singapore, Thailand, United States of America), and ethical approval was obtained from the relevant institutional ethics committees: NRES Committee North East – Sunderland (Central Ethics Committee MREC) (13/NE/0125), Ethikkommission Charité – Ethikausschuss 2 am Campus (EA2/063/13), Virchow Klinikum Ethikkommission Ärztekammer Nordrhein Düsseldorf (2013119), Ethik-Kommission der Medizinischen Fakultät der Ruhr Universität Bochum (4679-13), Comitato Etico per la Sperimentazione Clinica della Province di Verona e Rovigo (N. prog. 2321), Singhealth Centralised Institutional Review Board (CIRB) (2012/943/E), Institutional Review Board of the Faculty of Medicine, Chulalongkorn University (COA no. 00512013, IRB no. 505/55), Committee on Human Rights Related to Research Involving Human Subjects, Faculty of Medicine Ramathibodi Hospital, Mahidol University (MURA2012/569), Ethics Committee of the Faculty of Medicine, Prince of Songkla University (56-071-01-1-1) and Institutional Review Board for Human Subject Research for Baylor College of Medicine and Affiliated Hospitals (BCM IRB) (H-30791).

### 2.3 Clinical data

In total, 25 clinical variables were used in this study (Table S1, descriptive statistics for each clinical variable are presented in Table S2, ESI†).

### 2.4 16S rRNA gene amplicon sequencing and pre-processing

The V3–V4 region of the 16S rRNA gene was sequenced on DNA extracted from collected stool samples, and the raw sequences were pre-processed as reported elsewhere.^7^ A short summary of the procedure is provided in the ESI† (supplementary methods – Section S1, ESI†). The SILVA 138 database^9^ was used to assign taxonomy at the genus level to each amplicon sequence variant (ASV).

The ASVs were aggregated at genus level (resulting in 173 genera), and genera for which the sum of counts over all samples was lower than 3 were filtered out. On the remaining 145 genera, the robust centred log ratio (RCLR) transformation,^10^ implemented in the R (version 4.2.1)^11^ microbiome package^12^ (version 1.18.0), was applied to the counts to remove scale invariance and non-negativity. In contrast to the centred log ratio (CLR) transformation,^13^ the RCLR transformation is only applied to non-zeros and does not need addition of pseudo counts. This has the advantage that spurious correlations between variables caused by adding pseudo counts are avoided.

### 2.5 (Meta)proteomics data and pre-processing

Preparation of stool samples, nLC-MS/MS and identification of proteins were performed as described previously.^7^ A short summary of the procedure is provided in the ESI† (supplementary methods – Section S2, ESI†). In this way we obtained 2705 protein groups, of which 2481 were microbial.

Protein groups with sum of intensity Based Absolute Quantitation (iBAQ) values in all samples lower than 3 and contaminants were removed, resulting in 2435 microbial and 207 human protein groups. Subsequently, the microbial and human proteomics data was normalized using robust centred log ratio (RCLR) transformation.

### 2.6 Immune data

Saliva samples were analysed with the Olink® Target 96 Inflammation (v.3023) panel, and normalized protein expression (NPX) values were obtained as described elsewhere.^14^ A short summary of the procedure is provided in the ESI† (supplementary methods – Section S3, ESI†).

For the visit at 12 months, one sample (from an infant with persistent CMA) was missing. Immune factors below the limit of detection for more than 20% of the samples were filtered out, resulting in 58 immune factors.

### 2.7 Metabolomics data

#### 2.7.1 Sample preparation

A description of the sample preparation is provided in the ESI† (supplementary methods – Section S4, ESI†).

#### 2.7.2 Platform for polar to semi-polar metabolites

The analytical method was described previously.^15,16^ The platform for polar to semi-polar metabolites covers multiple classes, including acylcarnitines, amino acids, indoles and derivatives, nucleosides and nucleotide analogues, phenols and benzoic acids. Sample aliquoting, sample measurement with Ultra Performance Liquid Chromatography-high resolution mass spectrometry (UPLC-MS), target filtering and batch correction were carried out as described in the ESI† (supplementary methods – Section S5, ESI†).

#### 2.7.3 Platform for bile acids and fatty acids

For the platform for bile acids and fatty acids, aliquoting, UPLC-ToF, target filtering and batch correction were carried out as described in the ESI† (supplementary methods – Section S6, ESI†).

#### 2.7.4 Pre-processing

Metabolites with >20% missingness were filtered out. The filtered data consisted of 68 and 77 compounds from the platform for polar to semi-polar metabolites in positive and negative mode, respectively, and 22 from the platform for bile acids and fatty acids. Weight normalization by dry sample weight was applied on the filtered data. The data was log 2 transformed and missing values were imputed by quantile regression imputation of left-censored data (QRILC).^17^

### 2.8 Multi-view learning

In this study, we build a machine learning model to classify infants according to outgrowth of allergy status at 12 months, using clinical, 16S rRNA gene sequencing, (meta)proteomics, metabolomics and host immune data of the 39 infants with known allergy status at 12 months.

**Fig. 1:**
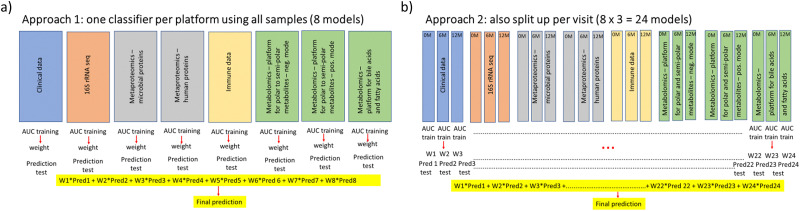
Two approaches for multi-view learning in this study. (a) 8 views are considered: clinical data, 16S rRNA sequencing, (meta)proteomics – microbial proteins, (meta)proteomics – human proteins, immune data, metabolomics – platform for polar to semi-polar metabolites in negative mode, metabolomics – platform for polar to semi-polar metabolites in positive mode and metabolomics – platform for bile acids and fatty acids. (b) Each of the 8 views is split up in 3 views, one for each visit. For both approaches, a machine learning classifier is trained on each view, and the area under the receiver operating characteristic curve (AUC) is calculated for the training set. A weight for each view is calculated based on the AUC. Wi represents the weight for the *i*-th view. Predi represents the predicted probabilities for the *i*-th view. The combined predicted probabilities are obtained by calculating the sum of the products of the weights and predicted probabilities for each view to obtain a final prediction for each class.

As we did not know beforehand whether outgrowth of cow's milk allergy can be predicted by the whole period from diagnosis to 12 months after diagnosis or by individual visits, two approaches were considered (see Fig. 1). In the first approach, 8 views are considered: clinical data, 16S rRNA sequencing data, (meta)proteomics data – microbial proteins, (meta)proteomics data – human proteins, immune data, metabolomics data – platform for polar to semi-polar metabolites in negative mode, metabolomics data – platform for polar to semi-polar metabolites in positive mode and metabolomics data – platform for bile acids and fatty acids. Each view includes the data from the three visits after pre-processing as described in the paragraphs above. In the second approach, each of the 8 views is split up in 3 views, one for each visit, resulting in 24 views in total. For both approaches, the data are split up in training and test set (see Section 2.8.1), a random forests classifier (see Section 2.8.3) is trained on the training set of each view and the area under the receiver operating characteristic curve (AUC) is calculated for the training set. A weight for each view is calculated by dividing the AUC for that view by the sum of AUCs over all views. Predicted probabilities for the samples in the test set are calculated for each class. The combined predicted probabilities are obtained by calculating the sum of the products of the weights and predicted probabilities for each view to obtain a final prediction for each class.

#### 2.8.1 Splitting the data into training and test set

Two third (26) of the subjects were used as training set, while the remaining one third (13) was used as test set.

To preserve the same proportions of samples in each of the two classes (outgrowth of CMA, persistent CMA) in training and test set, stratified splitting was used. As approach 1 includes repeated measurements, samples of the same subject were assigned all to the training set or all to the test set to take into account dependencies between samples from the same subject. In this way, the test set is independent from the training set, and no leakage of information can occur.

To assess the influence of the train-test split on the performance of the classifier, splitting was repeated 5 times.

#### 2.8.2 Cross validation

For each of the 5 train-test splits, 5-fold cross validation (CV) was performed by dividing the training set (26 subjects) into 5 parts (CV folds) using stratified splitting (such that the proportions of samples in each class were preserved). Samples from the same subjects were assigned to the same CV fold to take into account dependencies between samples of the same subject and avoid leakage of information between CV folds.

#### 2.8.3 Random forests classification

In this study, random forests^18^ was used as classification method as it is more suitable for handling data with small sample sizes compared to other methods.^19^ In all models, persistent CMA was defined as the positive class. Calculations were performed using the R (version 4.2.1)^11^ caret package (version 6.0-93).^20^ Models were evaluated based on the AUC, sensitivity and specificity. First, a random forests classifier was trained with default settings, and the influence of oversampling and Synthetic Minority Oversampling TEchnique (SMOTE),^21^ two methods for dealing with class imbalance, was assessed. Next, we studied the effect of removing variables with near zero variance and filtering out highly correlated predictors (using default: >0.9). Subsequently, the following parameters are optimized: mtry (number of variables to be considered at each split), ntree (number of trees) and the decision threshold (threshold for the predicted probability of the positive class, default 0.5). Compared to other methods for dealing with class imbalance, decision threshold moving has the advantage that it uses the original training set.^22^

After optimizing the parameters, the optimal combination of views for each of the two multi-view learning approaches was determined as follows. We first compared the AUC on the test set for each view to the AUC on the test set when combining all views. If there were AUCs for individual views that are larger than the AUC for the combination of all views, the following forward selection procedure was applied. Step 1: determine the individual classifier with the highest AUC on the test (AUC-test) set. Step 2: combine this classifier with the classifier of another view, and calculate the AUC of the test set (AUC-test-new). In case AUC-test-new > AUC-test, we keep this view in the combined classifier. In case AUC-test-new ≤ AUC-test, the view is removed from the combined classifier. Step 2 was repeated for all remaining views.

Forward selection is necessary for dealing with “combinatorial explosion” when having to try all combinations (16 777 470 in total, see Table S3, ESI†), which is in the order of 10^7^.

For the combined classifier with the highest AUC test, variable importance was determined in two ways. First, the mean decrease in node impurity (*i.e.* how well the trees in the random forests split the training data), given by the Gini index, was calculated. This method has several drawbacks. The Gini index overestimates the importance of features with a high number of unique values,^23^ it is specific for random forests and therefore does not allow comparison with other types of models. Furthermore, the Gini index is calculated on the training set, and does not give information on how important the variables are for predicting the class of the samples in the test set. Therefore, also permutation-based variable importance was determined. For each variable, a copy of the test set was created and the values of the selected variable were shuffled. The decrease in performance (AUC-test) caused by shuffling was calculated. These steps were repeated for 100 permutations and the mean decrease in AUC-test was reported, together with the standard deviation. Variables with average decrease in AUC-test >0.01 (1%) were reported as important.

## 3. Results

### 3.1 Comparison of multi-view learning approaches using the default settings

The two approaches (Fig. 1) were compared using the default settings for the parameters (mtry = square root of total number of features, ntree = 500 and decision threshold 0.5) for each view. Tables S4 and S5 (ESI†) show performance statistics (AUC, sensitivity and specificity) for the 5 different test sets, as well as their mean and standard deviation. The values of the sensitivity show that both classifiers fail to classify the infants with persistent CMA (positive class), which is the class with the lowest number of subjects (minority class). In contrast, the values for the specificity show good to excellent classification of the infants who outgrew CMA (negative class, majority class). Overall performance based on AUC is highly dependent on the train-test split and varies from failed classification (AUC < 0.6) to moderate (0.7 ≤ AUC < 0.8) for approach 1 and from failed to good (0.8 ≤ AUC < 0.9) for approach 2. On average, performance is poor (0.6 ≤ AUC < 0.7).

### 3.2 Effect of oversampling and SMOTE

Oversampling and SMOTE did not improve the overall performance of the classifiers. Tables S6 and S7 (ESI†) show that oversampling in general improved the trade-off between sensitivity and specificity, but decreased the overall performance (mean AUC) of the classifiers. For approach 2, not enough samples were available in the minority class to perform SMOTE. For approach 1, SMOTE improved the trade-off between sensitivity and specificity, but decreased the overall performance (mean AUC) (Table S8, ESI†). For these reasons, oversampling and SMOTE were not further considered in this study.

### 3.3 Effect of removing variables with near zero variance and filtering out highly correlated predictors

Tables S9–S12 (ESI†) show that removing variables with near zero variance and filtering out highly correlated predictors does not improve the performance of the classifiers. These options are therefore not further considered in this study

### 3.4 Effect of concatenating highly correlated views in approach 2

As in approach 2 there are many views, we also investigated the effect of concatenating highly correlated views in our revision. We plotted a correlation matrix and checked also the correlations between variables of the different views (Fig. S1, ESI†). Correlations between the three metabolomics views of the same visit were in general higher than correlations between other views. We therefore checked the influence of concatenating the three metabolomics views for each time point. Table S13 (ESI†) shows that this does not improve classification. Also in this case the classifier fails to classify the infants with persistent CMA (positive class).

### 3.5 Fitting of mtry, ntree and decision threshold

The parameters mtry and ntree were fitted on the training set for each view separately and reported in Tables S14 and S15 (ESI†), together with the mean AUC on the training and test set. The sensitivities and specificities on the training set of all models were combined by taking their geometric mean. The decision threshold that resulted in the highest geometric mean was selected. The optimal decision threshold for approach 1 and approach 2 was 0.38 and 0.40 respectively (Tables S16 and S17, ESI†). Tables S18 and S19 (ESI†) show the performance of the combined models with the optimal parameters.

For approach 1, both the overall performance and the trade-off between sensitivity and specificity are improved (compare Table S18 with Table S4 (ESI†)). For approach 2, only the trade-off between sensitivity and specificity is improved (compare Table S19 with Table S5 (ESI†)). When comparing Tables S18, S19 with Tables S14, S15 (ESI†), it appears that several classifiers for individual views have a higher overall performance (AUC-test) than the combined classifier. Therefore, the classifiers combining all views were not further considered, and there was screening for the best combination like described in Section 2.8.3.

### 3.6 Best combination of classifiers

Tables S20, S21 and Fig. S2, S3 (ESI†) show that for approach 1, the best performance as judged by AUC-test is obtained when combining the classifiers of the clinical data, microbial (meta)proteomics, metabolomics with platform for polar to semi-polar metabolites in negative mode and metabolomics with platform for polar to semi-polar metabolites in positive mode. For approach 2, the best classifier was obtained by combining metabolomics with platform for polar to semi-polar metabolites in positive mode at 12 months, clinical data at 6 months, 16S rRNA gene sequencing at 0 months, microbial (meta)proteomics at 0 months, immune data at 6 months, metabolomics with platform for polar to semi-polar metabolites in negative mode at 6 months and metabolomics with platform for polar to semi-polar metabolites in positive mode at 6 months. The performance of the best combined classifier for approach 1 and 2 is presented in Tables 1 and 2. For approach 1, the overall performance varies from failed classification (AUC < 0.6) to good (0.8 ≤ AUC < 0.9), depending on the train-test split. On average, the overall performance is poor (0.6 ≤ AUC < 0.7) (Table 1). Therefore approach 1 was not considered for determining variable importance. For approach 2, performance varied from poor (0.6 ≤ AUC < 0.7) to excellent (AUC > 0.9). On average the overall performance is good (0.8 ≤ AUC < 0.9), and there is also a good trade-off between sensitivity and specificity. However, the trade-off between sensitivity and specificity (at the optimal decision threshold based on the training sets) largely depends on the train-test split (Table 2).

**Table 1:** Performance of the best combined classifier for approach 1 (Fig. 1a). AUC, sensitivity and specificity for the five different test sets, together with the mean and the standard deviation (sd). Persistent CMA = positive class

Statistic	Set 1	Set 2	Set 3	Set 4	Set 5	Mean	sd
AUC	0.633	0.842	0.716	0.752	0.517	0.692	0.123
Sensitivity	0.857	0.867	0.800	0.714	0.667	0.781	0.088
Specificity	0.500	0.667	0.435	0.565	0.250	0.483	0.156

**Table 2:** Performance of the best combined classifier for approach 2 (Fig. 1b). AUC, sensitivity and specificity for the five different test sets, together with the mean and the standard deviation (sd). Persistent CMA = positive class

Statistic	Set 1	Set 2	Set 3	Set 4	Set 5	Mean	sd
AUC	0.667	1.000	0.800	1.000	0.875	0.868	0.141
Sensitivity	0.250	1.000	0.800	1.000	0.800	0.770	0.307
Specificity	0.667	0.875	0.571	0.857	0.500	0.694	0.168

### 3.7 Variable importance

Variable importance measures were calculated for the best model (the best combined classifier for approach 2 described in Section 3.5). In total, 2876 variables were used for training of the seven classifiers included in this model (68 for metabolomics with platform for polar to semi-polar metabolites in positive mode at 12 months, 25 for clinical data at 6 months, 145 for 16S rRNA gene sequencing at 0 months, 2435 for microbial (meta)proteomics at 0 months, 58 for immune data at 6 months, 77 for metabolomics with platform for polar to semi-polar metabolites in negative mode at 6 months, 68 for metabolomics with platform for polar to semi-polar metabolites in positive mode at 6 months).

#### 3.7.1 Mean decrease in node impurity (Gini index) (training sets)

As variables in the top 10 based on mean decrease in node impurity are less important for classifying new samples than those based on permutation-based importance, we have reported the detailed results in the ESI† (supplementary results – Section S1 and Table S22, ESI†).

#### 3.7.2 Permutation-based variable importance (test sets)

Table S23 (ESI†) presents the features with permutation-based variable importance >0.01 for each view per train-test split, and the features with mean permutation-based variable importance >0.01. The results largely differ between the models for the different train-test splits, both in number of features with variable importance >0.01 as in the features themselves. Therefore, the features with mean variable importance >0.01 were considered important for classification of samples on outgrowth of CMA and are summarized in Table 3 in order of importance. One hundred twenty-one important features, originating from multiple data types and visits, were identified as important. At baseline, several microbial genera (*e.g. Klebsiella*, *Haemophilus*, *Gemella*, *Dialister* and *Hungatella*) as well as several microbial protein groups (*e.g.* IMP cyclohydrolase in Clostridiales, *Blautia* spp., *Extibacter muris*, *Merdimonas faecis*, *Anaerostipes hadrus*, *Eisenbergiella* spp., *Enterocloster* spp., *Faecalicatena orotica* and *Ruminococcus bromii*) were important. At visit 6 months, important features included clinical factors (*e.g.* SCORAD (severity of atopic dermatitis), maternal and paternal allergy), human immune factors (*e.g.* 4E-binding protein 1 (4E-BP1), interleukin-1 alpha (IL-1 alpha) and C–X–C motif chemokine 5 (CXCL5)), and metabolites (*e.g.* myo-inositol/galactose/fructose, protocatechuic acid, *N*1-methyl-4-pyridone-3-carboxamide/nudifloramide and citrulline). At visit 12 months, important features included metabolites like citrulline, targinine/homoarginine, ornithine, threonine/homoserine and thymine. See Table 3 for full details.

**Table 3:** Features which presence in the ML model is important for classification of samples on outgrowth of CMA, having a mean permutation-based variable importance >0.01 (average decrease in AUC-test >1% after removal of the feature). Abbreviations: see Table S23 (ESI). The features for each view are presented in order of importance

Visit	Data	Features
Baseline	16S rRNA gene sequencing	*Klebsiella*, *Haemophilus*, *Gemella*, *Dialister*, *Hungatella*, *Lachnoclostridium*, *Bacteroides*, *Clostridium sensu stricto 1*, *Lachnospiraceae unclassified*, *TM7x*, *Streptococcus*, *Collinsella*, *Erysipelatoclostridium*, *Robinsoniella*

Baseline	Microbial (meta)proteomics	Protein groups:
		IMP cyclohydrolase in Clostridiales, *Blautia* spp, *Extibacter muris*, *Merdimonas faecis*, *Anaerostipes hadrus*, *Eisenbergiella* spp., *Enterocloster* spp., *Faecalicatena orotica* and *Ruminococcus bromii*
		DNA-directed RNA polymerase subunit beta in *Bifidobacterium* spp.
		Class II fructose-1,6-bisphosphate aldolase in *Anaerostipes hadrus* and *Lacrimispora amygdalina*
		GGGtGRT protein in Clostridiales, *Blautia* spp., *Ruminococcus flavefaciens* and *Clostridium chromiireducens*
		50S ribosomal protein L5 in Eubacteriales and more specific in *Anaerostipes hadrus*, *Clostridium perfringens*, *Faecalicatena orotica* and *Lachnospira pectinoschiza*
		50S ribosomal protein L16 in Eubacteriales and more specific in *Blautia* spp., *Mediterraneibacter glycyrrhizinilyticus*, *Roseburia* spp., *Enterocloster* spp, *Hungatella* spp., *Clostridium symbiosum*, *Faecalicatena orotica* and *Lachnospira* spp.

6 M	Clinical data	SCORAD, allergy of the father, allergy of the mother, skin prick test outcome wheat flour, number of antibiotics until visit, stool consistency, stool colour, stool frequency, treatment (AAF or AAF-syn), suspected allergy to wheat (yes/no), mode of delivery, age, number of infections until visit, skin prick test outcome soy bean, skin prick test outcome peanut, gas/wind and spitting

6 M	Immune data	4E-BP1, IL-1 alpha, CXCL5, CCL4, MCP-1, IL-12B, TGF-alpha, PD-L1, IL-15RA, LAP TGF-beta-1, STAMBP, EN-RAGE, CASP-8, TRAIL, TNFRSF9, CSF-1, OPG, LIF-R, CCL3, MMP-1, FGF-19, TNF, VEGF-A, CCL28, IL-7, OSM, Flt3L, IL-10RB and CCL19

6 M	Metabolomics platform for polar to semi-polar metabolites negative mode	myo-Inositol/galactose/fructose, protocatechuic acid, pyrocatechol, phenylacetic acid, 3-hydroxybutyric acid, N6-carboxymethyllysine, histidine, syringic acid, *trans*-aconitic acid, phenylacetylglutamine, *N*-acetylneuraminic acid, 2,5-furandicarboxylic acid, FAD, *N*-acetylneuraminic acid, gluconic acid, 2-hydroxyethanesulfonate, pseudouridine and xylulose.

6 M	Metabolomics platform for polar to semi-polar metabolites positive mode	*N*1-Methyl-4-pyridone-3-carboxamide/nudifloramide, citrulline, dodecanoylcarnitine, dihydrouracil, *N*6,*N*6,*N*6-trimethyllysine, guanidoacetic acid, betaine, 5-hydroxytryptophan, feature *m*/*z* 130.086 (unknown polar compound), serotonin, riboflavin, pyridoxal, picolinic acid, aspartic acid, beta-guanidinopropionic acid, 5-aminopentanoic acid, uracil and *N*-acetyltyrosine

12 M	Metabolomics platform for polar to semi-polar metabolites positive mode	Feature *m*/*z* 130.086, citrulline, targinine/homoarginine, ornithine, threonine/homoserine, thymine, 1-methyladenosine/*N*6-methyladenosine/2′-*o*-methyladenosine, ethanolamine, cadaverine, serotonin, sphinganine, pyridoxal, deoxyguanosine, 5-hydroxytryptophan, 5-aminolevulinic acid/4-hydroxyproline, *N*2,*N*2-dimethylguanosine, cytidine and thiamine

### 3.8 Comparison with early integration

Table 4 shows the performance of classification when concatenating all views from approach 1 (Fig. 1a) into a single data set. Overall performance was considerably lower than for our method, based on late integration and forward selection of views (compare Table 4 with Table 1). The overall performance varies from failed (AUC < 0.6) to poor (0.6 ≤ AUC < 0.7). On average, overall performance of the classifier failed (AUC < 0.6).

**Table 4:** Performance of classification when concatenating all views from approach 1 (Fig. 1a) into a single view (early integration). AUC, sensitivity and specificity for the five different test sets, together with the mean and the standard deviation (sd). Persistent CMA = positive class

Statistic	Set 1	Set 2	Set 3	Set 4	Set 5	Mean	sd
AUC	0.531	0.611	0.549	0.689	0.463	0.569	0.086
Sensitivity	0.786	0.733	0.933	0.857	0.533	0.769	0.152
Specificity	0.273	0.375	0.261	0.522	0.500	0.386	0.123

Concatenating all views from approach 2 into a single data set resulted in a much larger variation in performance between train-test splits, as well as a lower overall performance compared to late integration and forward selection of views (compare Table 5 with Table 2). The overall performance varied from failed (AUC < 0.6) to excellent (AUC > 0.9). On average, performance of the classifier is moderate (0.7 ≤ AUC < 0.8).

**Table 5:** Performance of classification when concatenating all views from approach 2 (Fig. 1b) into a single view (early integration). AUC, sensitivity and specificity for the five different test sets, together with the mean and the standard deviation (sd). Persistent CMA = positive class

Statistic	Set 1	Set 2	Set 3	Set 4	Set 5	Mean	sd
AUC	0.542	0.875	0.686	0.964	0.500	0.713	0.203
Sensitivity	0.750	0.800	1.000	1.000	0.600	0.830	0.172
Specificity	0.333	0.750	0.429	0.286	0.500	0.460	0.182

## 4 Discussion

In this study, we build a multi-view machine learning classifier for outgrowth of IgE-mediated CMA, using clinical, microbiome, (meta)proteomics, immune and metabolomics data. To the best of our knowledge, this is the first multi-omics machine learning study combining microbiome data with four other types of data. Considering the data from every visit as a different view for each platform (Fig. 1b) resulted in a better generalization performance than considering each platform as a different view (Fig. 1a). There are several possible reasons for this improvement. First, approach 1 assumes that the same features are the most important at all visits. However, allergic responses likely change over time, and different features might be important at different visits. Table S26 (ESI†) shows that in our study, for each separate-omics platform, the top 10 important variables based on Gini index differs between visits. Moreover, the top 10 for each visit differs from the top 10 when considering all visits as a single view. The differences in variable importance between visits can only be captured by approach 2, where each visit is modelled separately. Differences between visits within each allergy group have also been revealed by statistical analysis in our previous studies on the separate-omics data sets.^7,14,16^

Second, approach 1 can only include variables that are available for all time points/visits. However, for the clinical data, several variables were not available for all visits (*e.g.* the parent reported gastrointestinal outcomes, Table S1, ESI†). In contrast, approach 2 can include all variables that are available for at least one visit.

Furthermore, the results showed that combining all views did not improve the generalization performance of the best classifier for a single view. We therefore started with the best classifier for a single view and used forward selection to select the best combined classifier. The generalization performance for the best combined classifier (mean AUC-test = 0.868) was considerably better than for the best single view classifier (mean AUC-test = 0.690). Generalization performance depends largely on the train-test split (Table 2). Therefore, mean variable importance was considered to determine features important for classification. When comparing Tables S22 and S23 (ESI†), it can be noticed that some of the variables are in the top 10 based on Gini index, but do not reduce the generalization performance with >1%. These variables are less important for classifying new samples based on outgrowth of CMA and will not be further discussed. Several proteins belonging to protein groups important for classification are produced by genera important for classification, in particular by members of the genera *Clostridium sensu stricto 1* and *Hungatella*. These are GGGtGRT protein in *Clostridium chromiireducens*, 50S ribosomal protein L5 in *Clostridium perfringens*, 50S ribosomal protein L16 in *Hungatella* spp. and *Clostridium symbiosum*.

A search in Human Metabolome Database (HMDB)^24^ and Virtual Metabolic Human (VMH)^25^ revealed that the majority of metabolites important for classification are present in the microbes important for classification, or are a carbon source or a fermentation product of these microbes (Table S24, ESI†). According to the VMH database,^25^ phenylacetic acid can be produced by *Bacteroides*, and can be a carbon source for *Klebsiella*. The VMH database also indicates that three other metabolites important for classification are also a carbon source for *Klebsiella*: l-histidine, gluconic acid and l-aspartic acid. Furthermore, the VMH database reports *N*-acetylneuraminic acid is a carbon source for several microbes important for classification: *Haemophilus*, *Lachnoclostridium*, *Bacteroides*, *Clostridium sensu stricto 1*, *Streptococcus* and *Collinsella*.

Table S25 (ESI†) presents pathway information (KEGG^26^) for the microbial protein groups, immune factors and metabolites identified as important for classification. Several of these immune factors are part of pathways reported to be related to protection from allergens:^27^ Cytokine-cytokine receptor interaction (20 immune factors, see Table S25, ESI†), Toll-like receptor signalling pathway (CCL4, IL-12B, CASP-8, CCL3, TNF), Chemokine signalling pathway (CCL3, CCL4, MCP-1, MCP-4, CCL19, CCL28, CXCL5) and JAK-STAT signalling pathway (IL-12B, IL15-RA, LIF-R, IL-7, OSM, IL10-RB). Several other immune factors important for classification (TGF-alpha, IL15-RA, CSF-1, FGF-19, TNF, VEGF-A, Flt3L, IL1-alpha) belong to the MAPK signalling pathway, for which epigenetic changes have been related to food allergy.^28^ The detected variables important for classification also include immune factors belonging to the NF-kappa B signalling pathway, a pathway with an important role in the occurrence of allergic diseases by the release of inflammatory factors.^29^ Also members of two other signalling pathways involved in allergic inflammation, the PI3K-Akt (TGF-alpha, CSF-1, FGF-19, VEGF-A, IL-7, OSM, Flt3L, 4E-BP1) and NOD-like receptor signalling pathway (MCP-1, CASP-8, TNF),^29^ were detected as important for classifying infants based on outgrowth of CMA.

Serotonin, picolinic acid and 5-hydroxytryptophan are part of the tryptophan metabolism. Alterations of this pathway have been related to gut microbiome dysbiosis in CMA,^30^ and our results suggests that this pathway also differs between children who outgrew their allergy and those with persistent allergy. Our results suggest that also alterations of the following other pathways of amino acid metabolism could have a role in outgrowth of CMA: glycine, serine and threonine metabolism; arginine and proline metabolism; lysine degradation. Furthermore, the important variables for classifying infants based on outgrowth of CMA also included metabolites of the nucleotide metabolism (pseudouridine, dihydrouracil, uracil, thymine and cytidine). Members of the nucleotide metabolism, in particular the pyrimidine metabolism, were reported to have higher levels in people with IgE-mediated CMA.^30^

Although our approach was developed on data on CMA, it can also be used for other applications including microbiome and host multi-omics data. As an example, we use our approach to classify a subset of individuals from a study of Sailani *et al.*,^31^ for which the data were publicly available,^32^ into insulin resistant and insulin sensitive. The results are presented in Tables S27–S30 (ESI†) and show that approach 2 also outperforms approach 1 for this application.

Our study has several limitations. First, due to the small sample size, the generalization performance largely depends on the train-test split. We expect differences in generalization performance between train-test splits to be reduced in case of a larger sample size. Second, as all available data were from the same clinical trial, our study was restricted to vertical integration of data (*i.e.* integrating different types of data from the same samples). The availability of studies from other institutes measuring the same variables would give researchers the opportunity to perform horizontal data integration (across studies), which would also improve generalizability of the results.

Because of the limitations mentioned above, our results have to be considered as hypothesis-generating and require validation in larger, multi-center cohorts.

## 5. Conclusions

In summary, our study shows that vertical integration of microbiome data with clinical, immune, (meta)proteomics and metabolomics data could considerably improve classification of samples on outgrowth of CMA, compared to only considering one type of data. Variables identified as important for classification purposes were part of pathways that were related to the development of CMA in earlier studies.

## Author contributions

Diana M. Hendrickx: methodology, software, validation, formal analysis, writing – original draft; Mariyana V. Savova: methodology, resources, writing – review & editing; Pingping Zhu: methodology, resources, writing – review & editing; Ran An: methodology, resources, writing – review & editing; Sjef Boeren: methodology, resources, writing – review & editing; Kelly Klomp: methodology, software, writing – review & editing; Sumanth K. Mutte: methodology, software, writing – review & editing; PRESTO study team: resources; Harm Wopereis: resources, project administration, writing – review & editing; Renate G. van der Molen: resources, writing – review & editing; Amy C. Harms: resources, writing – review & editing; Clara Belzer: conceptualization, methodology, writing – review & editing, supervision, project administration, funding acquisition.

## Conflicts of interest

Harm Wopereis is an employee of Danone Nutricia Research. The project is part of a partnership programme between NWO-TTW and Danone Nutricia Research. The other authors declare that they have no known conflicts of interest.

## Supplementary Material

MO-021-D4MO00245H-s001

## Data Availability

Raw sequencing data are publicly available in the European Nucleotide Archive (ENA) (https://www.ebi.ac.uk/ena) under accession number PRJEB56782. Raw proteomics data and MaxQuant search results are publicly available from ProteomeXchange *via* the PRIDE^33^ partner repository (https://www.ebi.ac.uk/pride/) under accession number PXD037190. Metabolomics data are publicly available from MetaboLights (https://www.ebi.ac.uk/metabolights/) under accession number MTBLS8954. Clinical data are available from Danone Nutricia Research upon reasonable request (contact: Harm Wopereis, Harm.Wopereis@danone.com). Olink immune data are available as ESI,† (Gitlab folder) from another manuscript.^14^ All R code used in this study has been deposited in Gitlab: https://git.wur.nl/afsg-microbiology/publication-supplementary-materials/2024-hendrickx-et-al-earlyfit-presto-machine-learning.
