# Supplementary material for: A multi-omics machine learning classifier for outgrowth of cow's milk allergy in children
Source: Mol Omics. 2025 May 9;21(4):343–52. doi: 10.1039/d4mo00245h (PMC12101220; doi:10.1039/d4mo00245h)
Supplement: MO-021-D4MO00245H-s001 [file MO-021-D4MO00245H-s001.pdf]

Supplementary Information (SI) for Molecular Omics.
This journal is © The Royal Society of Chemistry 2025

Supplementary Material for: A multi-omics machine learning classifier for outgrowth of cow's milk allergy in children

Diana M. Hendrickx,‡[a] Mariyana V. Savova,[b] Pingping Zhu,[b] Ran An,§[a] Sjef Boeren,[c] Kelly Klomp,[a] Sumanth K. Mutte,¶[c] PRESTO study team, Harm Wopereis,[d] Renate G. van der Molen,[e] Amy C. Harms[b] and Clara Belzer*[a]

[a] Laboratory of Microbiology, Wageningen University, Wageningen, The Netherlands.

[b] Metabolomics and Analytics Centre, Leiden Academic Centre for Drug Research, Leiden University, Leiden, The Netherlands.

[c] Laboratory of Biochemistry, Wageningen University, Wageningen, The Netherlands.

[d] Danone Nutricia Research, Utrecht, The Netherlands.

[e] Department of Laboratory Medicine, Laboratory of Medical Immunology, Radboudumc, Nijmegen, The Netherlands.

*To whom correspondence should be addressed. Email: clara.belzer@wur.nl

‡ Current address: Institute for Risk Assessment Sciences (IRAS), Utrecht University, Utrecht, The Netherlands.

§ Current address: Department of Food Science and Technology, School of Agriculture and Biology, Shanghai Jiao Tong University, Shanghai, China.

¶ Current address: MyGen Informatics, 6706JE Wageningen, Netherlands

Supplementary methods

1. 16S rRNA gene amplicon sequencing and pre-processing

The 2 x 300 bp paired-end MiSeq protocol (Illumina) was used to obtain read pairs. Subsequently, read pairs were demultiplexed, low quality sequences were removed by trimming, and the trimmed reads were merged, dereplicated and counted. Next, filtering was applied to remove reads with < 2 reads over all samples, eliminate chimeras, and filter out reads including PhiX and Adapter sequences.

2. (Meta)proteomics data and pre-processing

Proteins were extracted and digested into peptides. Subsequently, samples were analyzed with nLC/MS-MS, and the obtained spectra were analyzed using MaxQuant 2.0.3.0 (Cox and Mann, 2008). The Andromeda search engine (Cox, *et al.*, 2011) was used with an in house microbial proteome database, a human database from Uniprot (Bateman, *et al.*, 2021) and a database of common contaminants (Hendrickx, *et al.*, 2023).

3. Immune data – preprocessing

The Olink® Target 96 Inflammation (v.3023) panel allows the analysis of 92 biomarkers related to inflammation and immune response. Samples were randomly distributed between two plates. Normalized protein expression (NPX) values were obtained as described previously (Hendrickx, *et al.*, 2024). Quality control (QC) of each sample was performed using four internal controls and evaluating the deviation from the median value of the controls. A QC warning was obtained for only four samples (of which two from visit 6 months and two from visit 12 months). Because these samples did not show up as

outliers in a principal components analysis (PCA) plot (Hendrickx, *et al.*, 2024), they were not removed from the data set.

4. Sample preparation - metabolomics

Stool samples were freeze-dried at 4 mbar for 20 hours. For all analytical platforms, a single dry-sample aliquot per sample (20.0 ± 0.3 mg) was used. Internal standards were added to the dry fecal matter prior to extraction. Single liquid-liquid extraction (LLE) with the MTBE method (MTBE/methanol/water, 3.6/2.8/3.5, v/v/v) was performed as described by (Hosseinkhani, *et al.*, 2021), with the volume of extraction solvent doubled based on the reported solvent-feces ratio. After LLE, the extraction layer from each sample was aliquoted for two analytical platforms. Study Quality Control (SQC) pool samples were prepared after extraction using 30 samples with sufficient amount of dry material left.

5. Metabolomics – platform for polar to semi-polar metabolites

Aliquoting of 150 μl aqueous layer was transferred to an Eppendorf tube and dried in a Speedvac. Next, the residue was reconstituted in 50ul of MilliQ water containing 0.1% formic acid. Analysis was carried out with a Shimadzu Nexera Prominence LC System coupled to a high-resolution triple TOF mass spectrometer (AB SCIEX triple TOF 6600). Separation was achieved using a (Ultra Performance Liquid Chromatography) UPLC HSS T3 column (Waters) at a flow rate of 0.4 mL/min with a 15 min gradient. Ion suppression was monitored by adding standards to the LC flow post-column. Data were collected in both positive and negative ion mode (TOF mass range of 60 - 800 Da). Data acquisition was performed using Analyst Software (AB SCIEX, Version 1.7.1). The data was integrated using SciexOS software (AB SCIEX, Version 2.2.0). Background signal was assessed using blank samples. Batch effect correction was carried out using the pooled SQC samples and in-house developed software. Metabolites with relative standard deviation of the SQC above 30% were filtered out.

6. Metabolomics – platform for bile acids and fatty acids

Aliquoting 48.8 μl of aqueous and 28.8 μl of organic layer was performed by transfer to an Eppendorf tube and drying in a Speedvac. Next, the residue was reconstituted in 200 μl of methanol. Analysis was carried out on ExionLC system coupled to a high-resolution TOF mass spectrometer (Zeno 7600 from Sciex). Separation was achieved on ACQUITY UPLC HSS T3 column from Waters (1.8 μm, 2.1 * 100mm) using a flow rate of 0.4 mL/min with a 15 min gradient. Data were collected in negative ion mode (200 – 900 m/z). Data acquisition and evaluation was carried out using SciexOS software (AB SCIEX, Version 2.2.0). Peak-picking was performed on the extracted ion chromatograms, and the relative concentrations for each target (ratio of target to isotopically labelled internal standards spiked before the extraction) were determined. Assessment of background signal, target filtering and batch effect correction were carried out in the same way as for the platform for polar and semi-polar metabolites.

Supplementary results

1. Variable importance based on mean decrease in node impurity (Gini index) (training sets)

Table S21 presents the top 10 for each view per train-test split, and top 10 based on mean variable importance. The results largely differ between the models for the different train-test splits. Only 17 variables occur in the top 10 of their view for all 5 models, and 11 variables occur in the top 10 of their view for 4 of the 5 models. The top 10 based on mean variable importance for the 16S rRNA gene sequencing at baseline consists of the following genera: *Klebsiella*; *Haemophilus*; *Clostridium sensu stricto 1*; *Hungatella*; *Streptococcus*; *Lachnospiraceae unclassified*; *Erysipelatoclostridium*; *Lachnoclostridium*; *TM7x*; *Bacteroides*. The top 10 for the microbial proteomics at baseline consists of the following protein groups: DNA-directed RNA polymerase subunit beta in *Bifidobacterium* spp.; Class II fructose-1,6-bisphosphate aldolase in *Anaerostipes hadrus* and *Lacrimispora amygdalina*; 50S ribosomal protein L27 in *Bifidobacterium* spp.; fumarate reductase/succinate dehydrogenase flavoprotein subunit in *Prevotella* and *Phocaeicola* spp.; IMP cyclohydrolase in Clostridiales, *Blautia* spp., *Extibacter muris*, *Merdimonas faecis*, *Anaerostipes hadrus*, *Eisenbergiella* spp., *Enterocloster* spp., *Faecalicatena orotica* and *Ruminococcus bromii*; 50S ribosomal protein L5 in Eubacteriales and more specific in *Anaerostipes hadrus*, *Clostridium perfringens*, *Faecalicatena orotica* and *Lachnospira pectinoschiza*; IMP cyclohydrolase in Clostridiales and Eubacteriales (more specific: *Lachnoclostridium pacaense*, *Ruminococcus champanellensis*, *Blautia luti*, *Enterocloster* spp.; sirohydrochlorin cobaltochelatase in Clostridiales; phosphoglycerate kinase in Clostridiales, *Blautia* spp., and Clostridium symbiosum; glutamate dehydrogenase in *Enterococcus devriesei*. The top 10 clinical features at 6 months were SCORAD (severity of atopic dermatitis), age, stool consistency, stool colour, number of infections until visit, allergy of the father, stool frequency, skin prick test outcome wheat flour (positive/negative), allergy mother and gas/wind. Top 10 immune factors at 6 months were interleukin-1 alpha (IL-1 alpha), eukaryotic translation initiation factor 4E-binding protein 1 (4E-BP1), C-X-C motif chemokine 5 (CXCL5), C-C motif chemokine 4 (CCL4), programmed cell death 1 ligand 1 (PD-L1), Monocyte chemotactic protein 4 (MCP-4), protein S100-A12 (EN-RAGE), tumor necrosis factor ligand superfamily member 14 (TNFSF14), tumor necrosis factor receptor superfamily member 9 (TNFRSF9) and leukemia inhibitory factor (LIF). Top 10 metabolites from the platform for polar and semi-polar metabolites in negative mode at 6 months are: protocatechuic acid, pyrocatechol, myo-inositol/galactose/fructose, syringic acid, 3-hydroxybutyric acid, phenylacetic acid, trans-aconitic acid, o-acetylserine/glutamic acid, dimethylglycine and gluconic acid. Top 10 metabolites from the platform for polar and semi-polar metabolites in positive mode at 6 months are: n6,n6,n6-trimethyllysine, citrulline, n1-methyl-4-pyridone-3-carboxamide/nudifloramide, guanidoacetic acid, serotonin, feature mz 130.086 (unknown polar compound with mass 130.086), beta-guanidinopropionic acid, dihydrouracil, dodecanoylcarnitine and betaine. Top 10 metabolites from the platform for polar and semi-polar metabolites in positive mode at 12 months are: feature mz 130.086, citrulline, ornithine, threonine/homoserine, serotonin, thymine, quinaldic acid, n-acetylcadaverine, pyridoxal and 1-methyladenosine/n6-nethyladenosine/2'-o-methyladenosine.

Supplementary tables and figures

Table S1. List of clinical variables used in this study

Variable	Explanation	baseline	6 months	12 months
sex	male/female	X		
age	age in months	X	X	X
delivery	mode of delivery (vaginal or ceasarian)	X		
alrgymot	Does the mother have allergy (yes/no)?	X		
alrgyfat	Does the father have allergy (yes/no)?	X		
sibl	Does the infant have a sibling (yes/no)?	X		
SCORADMAN	SCORAD (severity of atopic dermatitis) measured at visit	X	X	X
FOODTRIG2	Suspected allergy to egg (yes/no)	X		
FOODTRIG3	Suspected allergy to soy (yes/no)	X		
FOODTRIG4	Suspected allergy to wheat (yes/no)	X		
FOODTRIG5	Suspected allergy to peanut (yes/no)	X		
FOODTRIG7	Suspected allergy to other food than cow's milk, egg, soy, wheat, peanut, cod (yes/no)	X		
FOODTRIG8	Suspected allergy to other food than cow's milk, egg, soy, wheat, peanut, cod, other than mentioned in FOODTRIG7 (yes/no)	X		
SPTOSB	Skin prick test outcome soy bean (positive/negative)	X		X
SPTOWF	Skin prick test outcome wheat flour (positive/negative)	X		X
SPTOP	Skin prick test outcome peanut (positive/negative)	X		X
VOMITING	Parent reported outcome: vomiting<br>0 = none; 1 = 1-2 times/day; 2 = 3-4 times/day		X	X
SPITTING	Parent reported outcome: spitting<br>0 = none; 1 = spitting up after some feeds; 2 = spitting up after all feeds		X	X
STOOLFREQ	Parent reported outcome: stool frequency<br>0 = none; 1 = few; 2 = several; 3 = a lot		X	X
STOOLCONSIST	Parent reported outcome: stool consistency<br>0 = Severe diarrhoea; 1 = Diarrhoea; 2 = Normal; 3 = Constipation		X	X
STOOLCOLOUR	Parent reported outcome: stool colour<br>0 = green; 1 = yellow; 2 = brown; 3 = Dark brown / Blackish		X	X
GASWIND	Parent reported outcome: gas / wind<br>0 = none; 1 = mild; 2 = moderate; 3 = severe		X	X
num_ab	number of antibiotics until visit		X	X
num_inf	number of infections until visit		X	X
treatment	AAF-syn: amino acid-based formula + synbiotics;<br>AAF: standard amino acid-based formula		X	X

Table S2. Descriptive statistics for each clinical variable in Table S1. Numeric variables are presented as mean ± standard deviation; categorical variable are presented as number (%). P-value determined by a Fisher's exact test for binary variables and a two-sided Mann Whitney U-test for numeric variables. Bold: significant.

Variable	Outgrowth of CMA (n = 24)	Persistent CMA (n = 15)	Total (n = 39)	p-value
sex: female	8 (33%)	3 (20%)	11 (28%)	0.477
male	16 (67%)	12 (80%)	28 (72%)	
age: baseline	8.56 ± 3.04	9.68 ± 2.63	9.00 ± 2.90	0.254
6 months	14.62 ± 3.02	15.59 ± 2.54	14.99 ± 2.85	0.427
12 months	20.84 ± 3.05	21.88 ± 3.01	21.24 ± 3.03	0.411
delivery: vaginal	6 (25%)	7 (47%)	13 (33%)	0.185
Caesarian	18 (75%)	8 (53%)	26 (67%)	
alrgymot: yes	9 (38%)	10 (67%)	19 (49%)	0.105
no	15 (63%)	5 (33%)	20 (51%)	
alrgyfat: yes	6 (25%)	9 (60%)	15 (38%)	**0.044**
no	18 (75%)	6 (40%)	24 (62%)	
sibl: yes	18 (75%)	10 (67%)	28 (72%)	0.718
no	6 (25%)	5 (33%)	11 (28%)	
SCORADMAN: baseline	8.98 ± 14.41	16.27 ± 13.24	11.78 ± 14.25	**0.036**
6 months	5.46 ± 8.32	8.13 ± 9.67	6.49 ± 8.84	0.338
12 months	6.77 ± 8.25	10.37 ± 8.77	8.15 ± 8.52	0.218
FOODTRIG2: yes	9 (38%)	5 (33%)	14 (36%)	1.000
no	15 (63%)	10 (67%)	25 (64%)	
FOODTRIG3: yes	1 (4%)	1 (7%)	2 (5%)	1.000
no	23 (96%)	14 (93%)	37 (95%)	
FOODTRIG4: yes	1 (4%)	4 (27%)	5 (13%)	0.062
no	23 (96%)	11 (73%)	34 (87%)	
FOODTRIG5: yes	1 (4%)	1 (7%)	2 (5%)	1.000
no	23 (96%)	14 (93%)	37 (95%)	
FOODTRIG7: yes	1 (4%)	2 (13%)	3 (8%)	0.547
no	23 (96%)	13 (87%)	36 (92%)	
FOODTRIG8: yes	0 (0%)	1 (7%)	1 (3%)	0.385
no	24 (100%)	14 (93%)	38 (97%)	
SPTOSB				
baseline: positive	1 (4%)	3 (20%)	4 (10%)	0.279
negative	23 (96%)	12 (80%)	35 (90%)	
12 months: positive	1 (4%)	3 (20%)	4 (10%)	0.279
negative	23 (96%)	12 (80%)	35 (90%)	
SPTOWF				
baseline*: positive	2 (9%)	6 (40%)	8 (21%)	**0.039**
negative	21 (91%)	9 (60%)	30 (79%)	
12 months: positive	3 (13%)	6 (40%)	9 (23%)	0.063
negative	21 (88%)	9 (60%)	30 (77%)	

*: one missing in outgrowth class

Table S2. (continued)

Variable	Outgrowth of CMA (n = 24)	Persistent CMA (n = 15)	Total (n = 39)	p-value
SPTOP				
baseline: positive	5 (21%)	5 (33%)	10 (26%)	0.463
negative	19 (79%)	10 (67%)	29 (74%)	
12 months*: positive	8 (35%)	9 (60%)	17 (45%)	0.185
negative	15 (65%)	6 (40%)	21 (55%)	
VOMITING: 6 months	0.13 ± 0.29	0.16 ± 0.52	0.14 ± 0.39	0.854
12 months	0.04 ± 0.20	0.20 ± 0.43	0.10 ± 0.32	0.125
SPITTING: 6 months	0.13 ± 0.27	0.11 ± 0.43	0.12 ± 0.34	0.441
12 months	0.04 ± 0.15	0.00 ± 0.00	0.03 ± 0.12	0.274
STOOLFREQ: 6 months	1.03 ± 0.24	1.02 ± 0.96	1.03 ± 0.35	0.130
12 months	1.11 ± 0.60	0.48 ± 0.17	1.05 ± 0.49	0.636
STOOLCONSIST:				
6 months	1.60 ± 0.61	1.89 ± 0.84[#]	1.69 ± 0.70	0.171
12 months	1.92 ± 0.62[#]	1.97 ± 0.77[#]	1.94 ± 0.67	0.825
STOOLCOLOUR:				
6 months	1.44 ± 0.61	1.06 ± 0.96[#]	1.32 ± 0.92	0.096
12 months	1.30 ± 0.80[#]	1.89 ± 0.78[#]	1.52 ± 0.83	0.144
GASWIND: 6 months	0.79 ± 0.61	0.56 ± 0.63	0.70 ± 0.73	0.504
12 months	0.86 ± 1.61	0.56 ± 0.63	0.74 ± 0.62	0.103
num_ab: 6 months	0.63 ± 2.61	0.53 ± 1.41	0.59 ± 1.21	0.389
12 months	1.25 ± 3.61	2.40 ± 2.64	1.69 ± 2.12	0.212
num_inf: 6 months	1.25 ± 4.61	1.67 ± 1.45	1.41 ± 1.58	0.176
12 months	1.96 ± 5.61	3.00 ± 2.30	2.36 ± 2.31	0.096
treatment: AAF-syn	14 (58%)	9 (60%)	23 (59%)	1.000
AAF	10 (42%)	6 (40%)	16 (41%)	

*: one missing in outgrowth class

[#]: 3 missings

Table S3. Calculation of the number of combinations of views. ${}_nC_k = \frac{n!}{k!(n-k)!}$ where n! = n*(n-1)*…*2*1.

views (n)	number (k)	number of combinations (${}_nC_k$)		views (n)	number (k)	number of combinations (nCk)
8	1	8		24	1	24
8	2	28		24	2	276
8	3	56		24	3	2,024
8	4	70		24	4	10,626
8	5	56		24	5	42,504
8	6	28		24	6	134,596
8	7	8		24	7	346,104
8	8	1		24	8	735,471
	sum	255		24	9	1,307,504
				24	10	1,961,256
				24	11	2,496,144
				24	12	2,704,156
				24	13	2,496,144
				24	14	1,961,256
				24	15	1,307,504
				24	16	735,471
				24	17	346,104
				24	18	134,596
				24	19	42,504
				24	20	10,626
				24	21	2,024
				24	22	276
				24	23	24
				24	24	1
					sum	16,777,215
**total approach 1 + approach 2** = 255 + 16,777,215 **= 16,777,470**						

Table S4. Random forests classification with default settings (mtry = square root of total number of features, ntree = 500 and decision threshold 0.5). AUC, sensitivity and specificity for approach 1 (Figure 1a) for the five different test sets, together with the mean and the standard deviation (sd). Persistent CMA = positive class.

statistic	test set 1	test set 2	test set 3	test set 4	test set 5	mean	sd
**AUC**	0.549	0.722	0.649	0.724	0.458	0.620	0.115
**sensitivity**	0.071	0.000	0.133	0.000	0.067	0.054	0.056
**specificity**	0.818	1.000	0.957	1.000	0.875	0.930	0.081

Table S5. Random forests classification with default settings (mtry = square root of total number of features, ntree = 500 and decision threshold 0.5). AUC, sensitivity and specificity for approach 2 (Figure 1b) for the five different test sets, together with the mean and the standard deviation (sd). Persistent CMA = positive class.

statistic	test set 1	test set 2	test set 3	test set 4	test set 5	mean	sd
**AUC**	0.458	0.775	0.771	0.893	0.550	0.690	0.179
**sensitivity**	0.000	0.000	0.000	0.000	0.000	0.000	0.000
**specificity**	1.000	1.000	1.000	1.000	1.000	1.000	0.000

Table S6. Influence of oversampling on the performance of random forests classification – approach 1 (Figure 1a). Other settings as in Table S4.

statistic	test set 1	test set 2	test set 3	test set 4	test set 5	mean	sd
**AUC**	0.558	0.642	0.655	0.674	0.492	0.604	0.077
**sensitivity**	0.429	0.067	0.200	0.071	0.067	0.167	0.157
**specificity**	0.636	0.917	0.913	0.913	0.750	0.826	0.128

Table S7. Influence of oversampling on the performance of random forests classification – approach 2 (Figure 1b). Other settings as in Table S5.

statistic	test set 1	test set 2	test set 3	test set 4	test set 5	mean	sd
**AUC**	0.500	0.675	0.714	0.893	0.575	0.671	0.150
**sensitivity**	0.000	0.000	0.200	0.750	0.200	0.230	0.307
**specificity**	0.833	1.000	1.000	1.000	0.875	0.942	0.081

Table S8. Influence of SMOTE on the performance of random forests classification – approach 1 (Figure 1a). Other settings as in Table S4.

statistic	test set 1	test set 2	test set 3	test set 4	test set 5	mean	sd
**AUC**	0.581	0.622	0.690	0.711	0.478	0.616	0.093
**sensitivity**	0.571	0.067	0.333	0.214	0.133	0.264	0.199
**specificity**	0.591	0.875	0.783	0.826	0.667	0.748	0.117

Table S9. Influence of removing variables with near zero variance on the performance of random forests classification – approach 1 (Figure 1a). Other settings as in Table S4.

statistic	test set 1	test set 2	test set 3	test set 4	test set 5	mean	sd
**AUC**	0.536	0.714	0.612	0.739	0.472	0.615	0.114
**sensitivity**	0.143	0.000	0.067	0.071	0.067	0.070	0.051
**specificity**	0.818	1.000	0.957	1.000	0.875	0.930	0.081

Table S10. Influence of removing variables with near zero variance on the performance of random forests classification – approach 2 (Figure 1b). Other settings as in Table S5.

statistic	test set 1	test set 2	test set 3	test set 4	test set 5	mean	sd
**AUC**	0.458	0.775	0.714	0.893	0.575	0.683	0.170
**sensitivity**	0.000	0.000	0.000	0.000	0.000	0.000	0.000
**specificity**	1.000	1.000	1.000	1.000	1.000	1.000	0.000

Table S11. Influence of filtering out highly correlated predictors on the performance of random forests classification – approach 1 (Figure 1a). Other settings as in Table S4.

statistic	test set 1	test set 2	test set 3	test set 4	test set 5	mean	sd
**AUC**	0.526	0.703	0.609	0.736	0.492	0.613	0.107
**sensitivity**	0.143	0.000	0.133	0.071	0.067	0.083	0.058
**specificity**	0.818	1.000	0.913	0.957	0.875	0.913	0.071

Table S12. Influence of filtering out highly correlated predictors on the performance of random forests classification – approach 2 (Figure 1b). Other settings as in Table S5.

statistic	test set 1	test set 2	test set 3	test set 4	test set 5	mean	sd
**AUC**	0.458	0.775	0.771	0.893	0.550	0.690	0.179
**sensitivity**	0.000	0.000	0.000	0.000	0.000	0.000	0.000
**specificity**	1.000	1.000	1.000	1.000	1.000	1.000	0.000

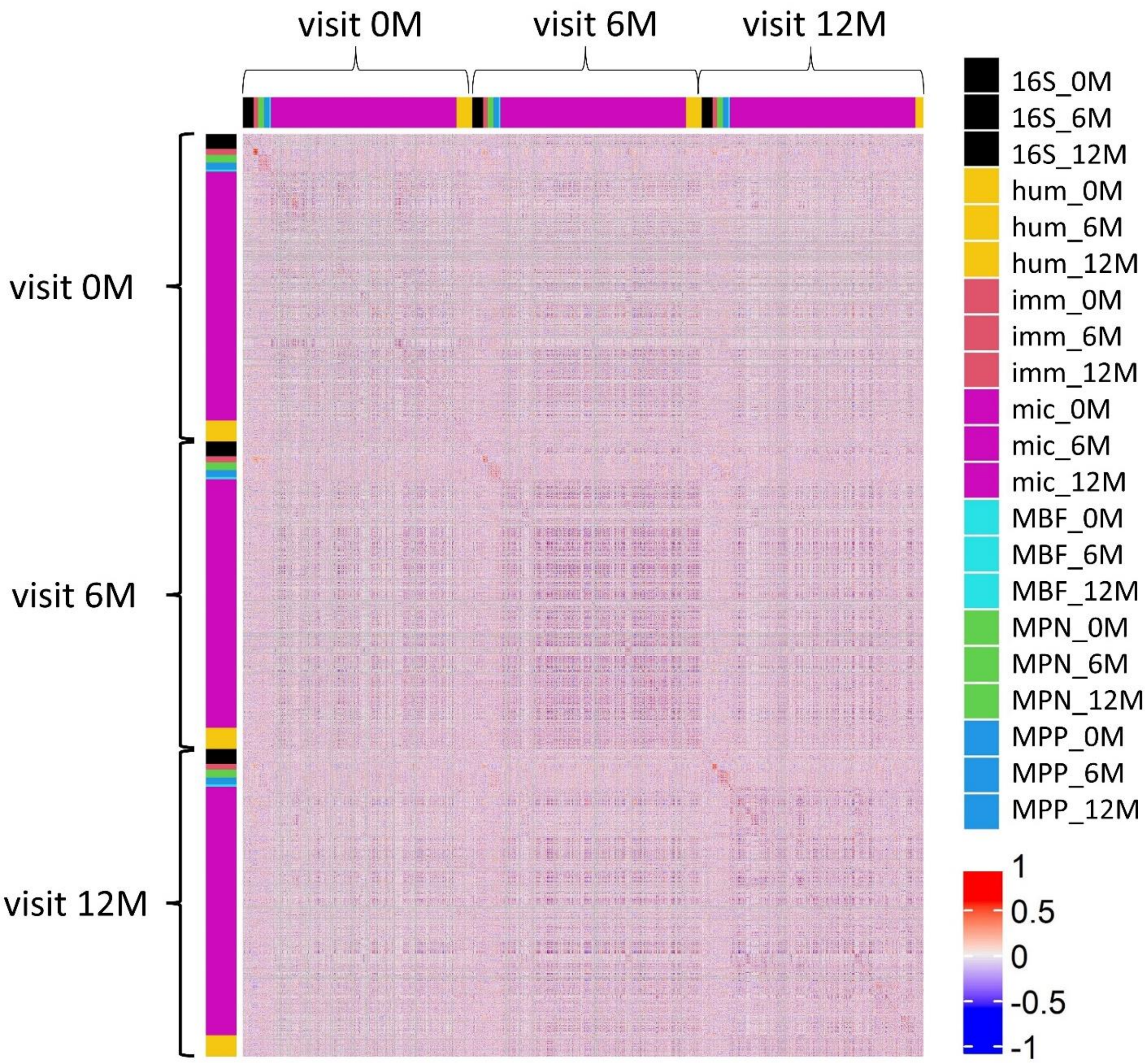

Figure S1. Spearman correlations between the variables from the 24 views in approach 2. Abbreviations: 16S: 16S rRNA gene sequencing; hum: metaproteomics – human proteins; imm: immune data; mic: metaproteomics – microbial proteins; MBF: metabolomics – platform for bile acids and fatty acids; MPN: metabolomics – platform for polar to semi-polar metabolites in negative mode; MPP: metabolomics – platform for polar to semi-polar metabolites in positive mode

Table S13. Influence of concatenating highly correlated views in approach 2 on the performance of random forests classification when using the default settings (see Table S5).

statistic	test set 1	test set 2	test set 3	test set 4	test set 5	mean	sd
**AUC**	0.542	0.800	0.686	0.786	0.575	0.678	0.188
**sensitivity**	0.000	0.000	0.000	0.000	0.000	0.000	0.000
**specificity**	1.000	1.000	1.000	1.000	1.000	1.000	0.000

Table S14. Fitted parameters mtry and ntree for each model in approach 1, together with their AUC on the training and test set.

model	mtry	ntree	AUC train	AUC test
**clinical data**	13	1500	0.612	0.649
**16S rRNA gene sequencing**	11	1000	0.529	0.521
**(meta)proteomics – microbial**	73	1000	0.539	0.591
**proteomics – human**	20	1000	0.563	0.552
**immune data**	14	2000	0.537	0.566
**metabolomics – platform for polar and semi-polar metabolites – negative mode**	12	1000	0.575	0.553
**metabolomics – platform for polar and semi-polar metabolites – positive mode**	4	1000	0.589	0.646
**metabolomics – platform for bile acids and fatty acids**	4	1000	0.555	0.536

Table S15. Fitted parameters mtry and ntree for each model in approach 2, together with their AUC on the training and test set.

model	mtry	ntree	AUC train	AUC test
**clinical data – 0M**	10	1000	0.621	0.620
**clinical data – 6M**	5	1000	0.603	0.665
**clinical data – 12M**	4	2500	0.607	0.595
**16S rRNA gene sequencing – 0M**	16	1500	0.525	0.655
**16S rRNA gene sequencing – 6M**	9	1000	0.467	0.625
**16S rRNA gene sequencing – 12M**	11	1500	0.483	0.515
**(meta)proteomics – microbial – 0M**	49	1000	0.644	0.688
**(meta)proteomics – microbial – 6M**	59	1000	0.487	0.635
**(meta)proteomics – microbial – 12M**	79	1000	0.491	0.545
**(meta)proteomics – human – 0M**	15	1000	0.622	0.543
**(meta)proteomics – human – 6M**	11	1000	0.556	0.570
**(meta)proteomics – human – 12M**	11	1500	0.449	0.625
**immune data – 0M**	9	1500	0.479	0.578
**immune data – 6M**	11	1500	0.502	0.628
**immune data – 12M**	15	2000	0.527	0.525
**metabolomics – platform for polar and semi-polar metabolites – negative mode – 0M**	9	1000	0.514	0.585
**metabolomics – platform for polar and semi-polar metabolites – negative mode – 6M**	13	1500	0.612	0.738
**metabolomics – platform for polar and semi-polar metabolites – negative mode – 12M**	11	2000	0.561	0.665
**metabolomics – platform for polar and semi-polar metabolites – positive mode – 0M**	5	1000	0.491	0.615
**metabolomics – platform for polar and semi-polar metabolites – positive mode – 6M**	3	1500	0.620	0.710
**metabolomics – platform for polar and semi-polar metabolites – positive mode – 12M**	14	2000	0.556	0.745
**metabolomics – platform for bile acids and fatty acids – 0M**	8	2000	0.488	0.650
**metabolomics – platform for bile acids and fatty acids – 6M**	3	1500	0.510	0.645
**metabolomics – platform for bile acids and fatty acids – 12M**	4	1000	0.642	0.639

Table S16. Geometric mean of sensitivity and specificity for decision thresholds between 0.3 and 0.5 when using approach 1 for multi-view learning. Bold = optimal decision threshold.

**threshold**	**geometric mean**
0.3	0.4667
0.31	0.4839
0.32	0.4945
0.33	0.5027
0.34	0.5127
0.35	0.5206
0.36	0.5274
0.37	0.5316
**0.38**	**0.5344**
0.39	0.5334
0.4	0.5315
0.41	0.5270
0.42	0.5226
0.43	0.5191
0.44	0.5107
0.45	0.5019
0.46	0.4914
0.47	0.4771
0.48	0.4647
0.49	0.4510
0.5	0.4350

Table S17. Geometric mean of sensitivity and specificity for decision thresholds between 0.3 and 0.5 when using approach 2 for multi-view learning. Bold = optimal decision threshold.

threshold	geometric mean
0.3	0.367
0.31	0.393
0.32	0.414
0.33	0.438
0.34	0.451
0.35	0.467
0.36	0.480
0.37	0.488
0.38	0.497
0.39	0.505
**0.4**	**0.509**
0.41	0.507
0.42	0.502
0.43	0.496
0.44	0.486
0.45	0.471
0.46	0.450
0.47	0.435
0.48	0.414
0.49	0.389
0.5	0.366

Table S18. Random forests classification with optimal mtry, ntree and decision threshold (See Tables S14 and S16). AUC, sensitivity and specificity for approach 1 (Figure 1a) for the five different test sets, together with the mean and the standard deviation (sd). Persistent CMA = positive class.

statistic	test set 1	test set 2	test set 3	test set 4	test set 5	mean	sd
**AUC**	0.571	0.719	0.649	0.714	0.475	0.626	0.103
**sensitivity**	0.857	0.933	0.933	0.857	0.667	0.850	0.109
**specificity**	0.273	0.417	0.217	0.522	0.333	0.352	0.120

Table S19. Random forests classification with optimal mtry, ntree and decision threshold (See Tables S15 and S17). AUC, sensitivity and specificity for approach 2 (Figure 1b) for the five different test sets, together with the mean and the standard deviation (sd). Persistent CMA = positive class.

statistic	test set 1	test set 2	test set 3	test set 4	test set 5	mean	sd
**AUC**	0.458	0.750	0.771	0.893	0.575	0.690	0.172
**sensitivity**	0.500	0.800	0.800	1.000	0.600	0.740	0.195
**specificity**	0.500	0.500	0.429	0.429	0.500	0.471	0.039

Table S20. Forward selection of best combination of views for approach 1. AUC-test: mean AUC over the 5 test sets. Red = removed because lowers performance. Blue = kept because increases performance. Bold = best classifier.

**Combination**	**AUC test**
Combine all data	0.626
Clinical data (best individual classifier)	0.649
Clinical data + 16S rRNA gene sequencing	0.643 (< 0.649)
Clinical data + microbial (meta)proteomics	0.672
Clinical data + microbial (meta)proteomics + human proteomics	0.654 (< 0.672)
Clinical data + microbial (meta)proteomics + immune data	0.644 (< 0.672)
Clinical data + microbial (meta)proteomics + metabolomics platform for polar and semi-polar metabolites – negative mode	0.684
**Clinical data + microbial (meta)proteomics + metabolomics platform for polar and semi-polar metabolites – negative mode + metabolomics platform for polar and semi-polar metabolites – positive mode**	**0.692**
Clinical data + microbial (meta)proteomics + metabolomics platform for polar and semi-polar metabolites – negative mode + metabolomics platform for polar and semi-polar metabolites – positive mode + metabolomics – platform for bile acids and fatty acids	0.681 (< 0.692)

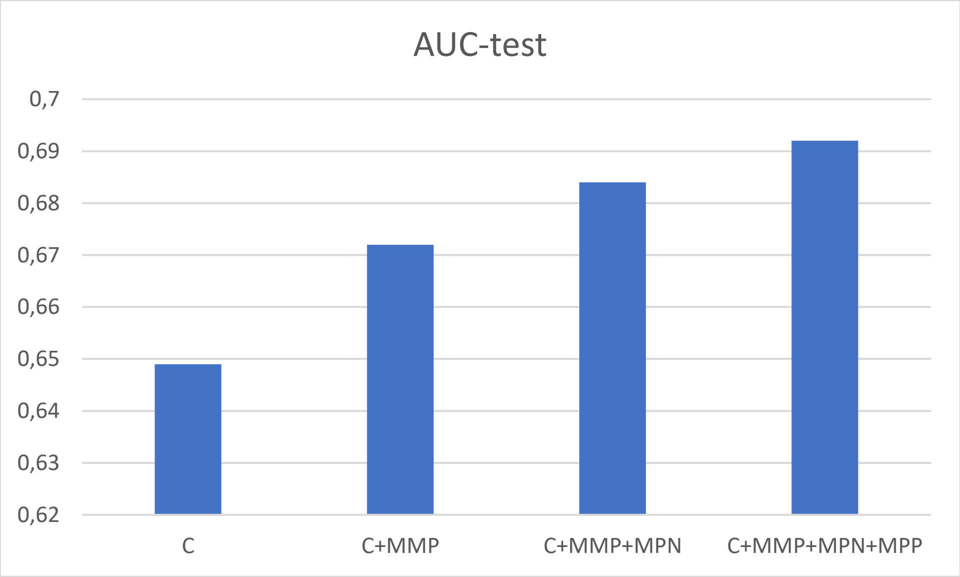

Figure S2. Increase in AUC-test for approach 1 when adding extra classifiers. C: clinical data; MMP: microbial (meta)proteomics; MPN: metabolomics platform for polar and semi-polar metabolites – negative mode; MPP: metabolomics platform for polar and semi-polar metabolites – positive mode.

Table S21. Forward selection of best combination of views for approach 2. AUC-test: mean AUC over the 5 test sets. Red = removed because lowers performance. Blue = kept because increases performance. Bold = best classifier.

**Combination**	**AUC test**
Combine all data	0.690
Metabolomics – platform for polar and semi-polar metabolites – positive mode 12M (best individual classifier)	0.745
Metabolomics – platform for polar and semi-polar metabolites – positive mode 12M + clinical data 0M	0.712 (< 0.745)
Metabolomics – platform for polar and semi-polar metabolites – positive mode 12M + clinical data 6M	0.767
Metabolomics – platform for polar and semi-polar metabolites – positive mode 12M + clinical data 6M + clinical data 12M	0.700 (< 0.767)
Metabolomics – platform for polar and semi-polar metabolites – positive mode 12M + clinical data 6M + 16S rRNA gene sequencing 0M	0.794
Metabolomics – platform for polar and semi-polar metabolites – positive mode 12M + clinical data 6M + 16S rRNA gene sequencing 0M + 16S rRNA gene sequencing 6M	0.773 (< 0.794)
Metabolomics – platform for polar and semi-polar metabolites – positive mode 12M + clinical data 6M + 16S rRNA gene sequencing 0M + 16S rRNA gene sequencing 12M	0.781 (< 0.794)
Metabolomics – platform for polar and semi-polar metabolites – positive mode 12M + clinical data 6M + 16S rRNA gene sequencing 0M + microbial proteomics 0M	0.838
Metabolomics – platform for polar and semi-polar metabolites – positive mode 12M + clinical data 6M + 16S rRNA gene sequencing 0M + microbial proteomics 0M + microbial proteomics 6M	0.808 (< 0.838)
Metabolomics – platform for polar and semi-polar metabolites – positive mode 12M + clinical data 6M + 16S rRNA gene sequencing 0M + microbial proteomics 0M + microbial proteomics 12M	0.823 (< 0.838)
Metabolomics – platform for polar and semi-polar metabolites – positive mode 12M + clinical data 6M + 16S rRNA gene sequencing 0M + microbial proteomics 0M + human proteomics 0M	0.819 (< 0.838)
Metabolomics – platform for polar and semi-polar metabolites – positive mode 12M + clinical data 6M + 16S rRNA gene sequencing 0M + microbial proteomics 0M + human proteomics 6M	0.808 (< 0.838)
Metabolomics – platform for polar and semi-polar metabolites – positive mode 12M + clinical data 6M + 16S rRNA gene sequencing 0M + microbial proteomics 0M + human proteomics 12M	0.798 (< 0.838)
Metabolomics – platform for polar and semi-polar metabolites – positive mode 12M + clinical data 6M + 16S rRNA gene sequencing 0M + microbial proteomics 0M + immune data 0M	0.783 (< 0.838)
Metabolomics – platform for polar and semi-polar metabolites – positive mode 12M + clinical data 6M + 16S rRNA gene sequencing 0M + microbial proteomics 0M + immune data 6M	0.853
Metabolomics – platform for polar and semi-polar metabolites – positive mode 12M + clinical data 6M + 16S rRNA gene sequencing 0M + microbial proteomics 0M + immune data 6M + immune data 12M	0.828 (< 0.853)

Table S21. (continued)

Combination	AUC test
Metabolomics – platform for polar and semi-polar metabolites – positive mode 12M + clinical data 6M + 16S rRNA gene sequencing 0M + microbial proteomics 0M + immune data 6M + metabolomics – platform for polar and semi-polar metabolites – negative mode 0M	0.806 (< 0.853)
Metabolomics – platform for polar and semi-polar metabolites – positive mode 12M + clinical data 6M + 16S rRNA gene sequencing 0M + microbial proteomics 0M + immune data 6M + metabolomics – platform for polar and semi-polar metabolites – negative mode 6M	0.868 (0.8676)
Metabolomics – platform for polar and semi-polar metabolites – positive mode 12M + clinical data 6M + 16S rRNA gene sequencing 0M + microbial proteomics 0M + immune data 6M + metabolomics – platform for polar and semi-polar metabolites – negative mode 6M + metabolomics – platform for polar and semi-polar metabolites – negative mode 12M	0.849
Metabolomics – platform for polar and semi-polar metabolites – positive mode 12M + clinical data 6M + 16S rRNA gene sequencing 0M + microbial proteomics 0M + immune data 6M + metabolomics – platform for polar and semi-polar metabolites – negative mode 6M + metabolomics – platform for polar and semi-polar metabolites – positive mode 0M	0.823
**Metabolomics – platform for polar and semi-polar metabolites – positive mode 12M + clinical data 6M + 16S rRNA gene sequencing 0M + microbial proteomics 0M + immune data 6M + metabolomics – platform for polar and semi-polar metabolites – negative mode 6M + metabolomics – platform for polar and semi-polar metabolites – positive mode 6M**	**0.868 (0.8683)**
Metabolomics – platform for polar and semi-polar metabolites – positive mode 12M + clinical data 6M + 16S rRNA gene sequencing 0M + microbial proteomics 0M + immune data 6M + metabolomics – platform for polar and semi-polar metabolites – negative mode 6M + metabolomics – platform for polar and semi-polar metabolites – positive mode 6M + metabolomics – platform for bile acids and fatty acids 0M	0.825 (< 0.868)
Metabolomics – platform for polar and semi-polar metabolites – positive mode 12M + clinical data 6M + 16S rRNA gene sequencing 0M + microbial proteomics 0M + immune data 6M + metabolomics – platform for polar and semi-polar metabolites – negative mode 6M + metabolomics – platform for polar and semi-polar metabolites – positive mode 6M + metabolomics – platform for bile acids and fatty acids 6M	0.830 (< 0.868)
Metabolomics – platform for polar and semi-polar metabolites – positive mode 12M + clinical data 6M + 16S rRNA gene sequencing 0M + microbial proteomics 0M + immune data 6M + metabolomics – platform for polar and semi-polar metabolites – negative mode 6M + metabolomics – platform for polar and semi-polar metabolites – positive mode 6M + metabolomics – platform for bile acids and fatty acids 12M	0.842 (< 0.868)

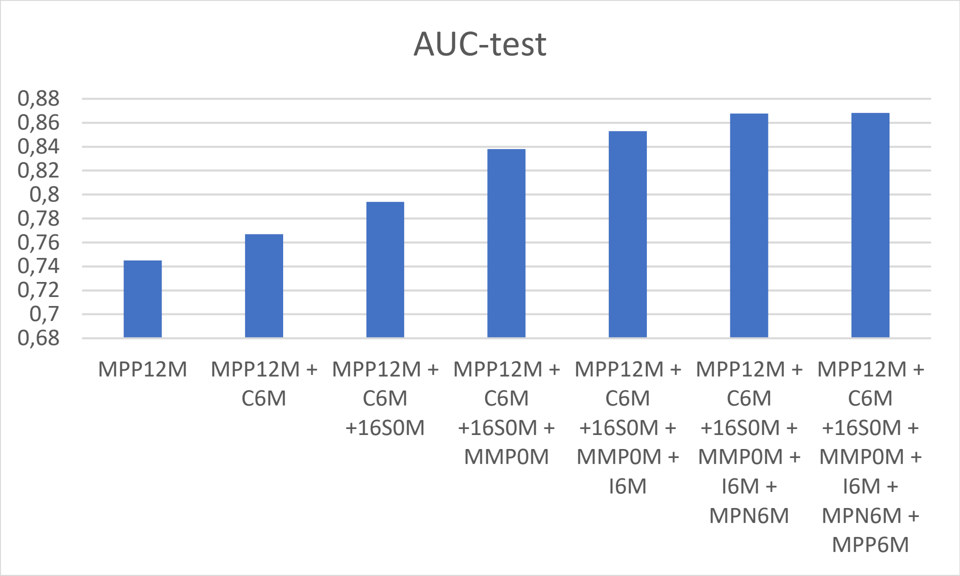

Figure S3. Increase in AUC-test for approach 2 when adding extra classifiers. MPP12M: metabolomics – platform for polar and semi-polar metabolites – positive mode 12M; C6M: clinical data 6M; 16S0M: 16S rRNA gene sequencing 0M; MMP0M: microbial (meta)proteomics 0M; I6M: immune data 6M; MPN6M: metabolomics – platform for polar and semi-polar metabolites – negative mode 6M; MPP6M: metabolomics – platform for polar and semi-polar metabolites – positive mode 6M.

Table S22. Variable importance based on mean decrease in node impurity (Gini index) (training sets). Top 10 for each view in the best model (Table S20) per train-test split, and top 10 based on mean variable importance.

Table S22A. 16S rRNA gene sequencing 0M

set 1	set 2	set 3	set 4	set 5	mean
Klebsiella	*Hungatella*	*Klebsiella*	*Erysipelatoclostridium*	*Klebsiella*	*Klebsiella*
Haemophilus	*Lachnospiraceae_unclassified*	*Haemophilus*	*Incertae_Sedis*	*Clostridium_sensu_stricto_1*	*Haemophilus*
Clostridium_sensu_stricto_1	*Anaerostipes*	*Streptococcus*	*Haemophilus*	*Haemophilus*	*Clostridium_sensu_stricto_1*
TM7x	*Gemella*	*Veillonella*	*Actinomyces*	*Enterobacter*	*Hungatella*
Enterococcus	*Clostridium_sensu_stricto_1*	*TM7x*	*Hungatella*	*Streptococcus*	*Streptococcus*
Akkermansia	*Lachnoclostridium*	*Lachnospiraceae_unclassified*	*Klebsiella*	*Bacteroides*	*Lachnospiraceae_unclassified*
Anaerostipes	*Klebsiella*	*Clostridium_sensu_stricto_1*	*Bacteroides*	*TM7x*	*Erysipelatoclostridium*
Blautia	*Dialister*	*Hungatella*	*Erysipelotrichaceae_ge*	*Lachnoclostridium*	*Lachnoclostridium*
Enterobacter	*Streptococcus*	*Dialister*	*Dialister*	*Eisenbergiella*	*TM7x*
Hungatella	*Haemophilus*	*Granulicatella*	*Veillonella*	*Erysipelatoclostridium*	*Bacteroides*

(yellow)	in top 10 for all 5 models
(light green)	in top 10 for 4 models

Table S22B. microbial proteomics 0M.

In the first table below, Fasta Maxquant for each protein group is abbreviated. For more details (full Fasta Maxquant, Protein Ids and protein names, see the next tables.

set 1	set 2	set 3	set 4	set 5	mean
tr.WP_015525727.1.	tr.A0A5B3GKY1.	tr.D1PG79.	tr.WP_008702995.1.	tr.A0A0A1GRX3.	tr.A0A0A1GRX3.
tr.A0A0A1GRX3.	tr.A0A0A1GRX3.	tr.A0A0A1GRX3.	tr.A0A2Y9BLV7.	tr.A0A173R7M3.	tr.A0A5B3GKY1.
tr.WP_195360800.1.	tr.A0A1V8Q6G3.	tr.WP_054704175.1.	tr.WP_008705693.1.	tr.S0GNC3.	tr.A0A1V8Q6G3.
tr.WP_008705693.1.	tr.WP_022380221.1.	tr.A0A3E4U7M2.	tr.WP_025577282.1.	tr.A0A369M4T5.	tr.D1PG79.
tr.A0A5B3GKY1.	tr.D4MV01.	tr.WP_173767105.1.	tr.WP_173773355.1.	tr.WP_173773355.1.	tr.WP_008705693.1.
tr.A0A1L8SSH0.	tr.D4BP81.	tr.A0A1V8Q6G3.	tr.D4BRG5.	tr.WP_008703889.1.	tr.A0A173R7M3.
tr.A0A173R7M3.	tr.WP_195360800.1.	tr.A0A1V8PLT8.	tr.A6KXV7.	tr.A0A1V8Q6G3.	tr.WP_173773355.1.
tr.A5ZUS4.	tr.A0A087DNV3.	tr.A0A5B3GKY1.	tr.A0A4P8KHU0.	tr.WP_119239543.1.	tr.WP_008702995.1.
tr.D4BNB9.	tr.D1PG79.	tr.WP_008705693.1.	tr.A0A3E3EA02.	tr.A0A133LTJ9.	tr.WP_025577282.1.
tr.A0A1V8Q6G3.	tr.A0A369M4T5.	tr.WP_119239543.1.	tr.A0A564W8K3.	tr.A0A1L8SSH0.	tr.A0A1L8SSH0.

(light green)	in top 10 for 4 models

Abbreviation	Fasta Maxquant
tr.WP_015525727.1.	tr.WP_015525727.1.WP_015525727.1_NCBI.MULTISPECIES..GGGtGRT.protein..Clostridiales..tr.A5ZUI6. A5ZUI6_9FIRM.Uncharacterized.protein.OS.Blautia.obeum.ATCC.29174.OX.411459.GN.RUMOBE_02671. PE.4.SV.1.tr.A0A564W402.A0A564W402_9FIRM.Uncharacterized.protein.OS.Bla
tr.A0A0A1GRX3	tr.A0A0A1GRX3.A0A0A1GRX3_BIFLN.DNA.directed.RNA.polymerase.subunit.beta.OS.Bifidobacterium. longum.OX.216816.GN.rpoB.PE.3.SV.1.tr.A0A1S2VY79.A0A1S2VY79_BIFLI.DNA.directed.RNA.polymerase. subunit.beta.OS.Bifidobacterium.longum.subsp..infantis.OX.1682.GN.rpoB
tr.WP_195360800.1.	tr.WP_195360800.1.WP_195360800.1_NCBI.DUF5458.family.protein..Phocaeicola.massiliensis.
tr.WP_008705693.1.	tr.WP_008705693.1.WP_008705693.1_NCBI.MULTISPECIES..IMP.cyclohydrolase..Clostridiales..tr. WP_173726912.1.WP_173726912.1_NCBI.IMP.cyclohydrolase..Blautia.glucerasea..tr.WP_117854094.1. WP_117854094.1_NCBI.MULTISPECIES..IMP.cyclohydrolase..Clostridiales..tr.A
tr.A0A5B3GKY1.	tr.A0A5B3GKY1.A0A5B3GKY1_ANAHA.Class.II.fructose.1.6.bisphosphate.aldolase.OS.Anaerostipes.hadrus. OX.649756.GN.fba.PE.4.SV.1.tr.WP_144365668.1.WP_144365668.1_NCBI.class.II.fructose.1.6.bisphosphate. aldolase..Lacrimispora.amygdalina.
tr.A0A1L8SSH0.	tr.A0A1L8SSH0.A0A1L8SSH0_9ENTE.Glutamate.dehydrogenase.OS.Enterococcus.devriesei.OX.319970.GN. RV00_GL003076.PE.3.SV.1
tr.A0A173R7M3.	tr.A0A173R7M3.A0A173R7M3_ANAHA.50S.ribosomal.protein.L5.OS.Anaerostipes.hadrus.OX.649756.GN.rplE. PE.3.SV.1
tr.A5ZUS4.	tr.A5ZUS4.A5ZUS4_9FIRM.Putative.carbamoyltransferase.YgeW.OS.Blautia.obeum.ATCC.29174.OX.411459.GN. ygeW.PE.3.SV.1
tr.D4BNB9.	tr.D4BNB9.D4BNB9_BIFBR.Carbamoyl.phosphate.synthase.large.chain.OS.Bifidobacterium.breve.DSM.20213... JCM.1192.OX.518634.GN.carB.PE.3.SV.1
tr.A0A1V8Q6G3.	tr.A0A1V8Q6G3.A0A1V8Q6G3_9BIFI.50S.ribosomal.protein.L27.OS.Bifidobacterium.dentium.OX.1689.GN.rpmA. PE.3.SV.1.tr.A0A1V8PSH8.A0A1V8PSH8_9BIFI.50S.ribosomal.protein.L27.OS.Bifidobacterium.catenulatum.OX. 1686.GN.rpmA.PE.3.SV.1.tr.A0A087DLM1.A0A087DLM1_BIFAD.5
tr.WP_022380221.1.	tr.WP_022380221.1.WP_022380221.1_NCBI.MULTISPECIES..carbamoyl.phosphate.synthase.large.subunit.. Clostridiales..tr.WP_173765213.1.WP_173765213.1_NCBI.MULTISPECIES..carbamoyl.phosphate.synthase.large. subunit..Clostridiales..tr.WP_173727273.1.WP_173727273.1_N
tr.D4MV01.	tr.D4MV01.D4MV01_ANAHA.NADH.peroxidase.OS.Anaerostipes.hadrus.OX.649756.GN.rbr3A.PE.4.SV.1
tr.D4BP81.	tr.D4BP81.D4BP81_BIFBR.Chorismate.synthase.OS.Bifidobacterium.breve.DSM.20213...JCM.1192.OX.518634. GN.aroC.PE.3.SV.1.tr.A0A0M3T6K3.A0A0M3T6K3_BIFLI.Chorismate.synthase.OS.Bifidobacterium.longum.subsp.. infantis.OX.1682.GN.aroC.PE.3.SV.1.tr.A0A0A1GRJ0.A0A0A1
tr.A0A087DNV3.	tr.A0A087DNV3.A0A087DNV3_BIFAD.ABC.transporter.substrate.binding.protein.OS.Bifidobacterium.stercoris. JCM.15918.OX.1437612.GN.BSTER_1210.PE.4.SV.1.tr.A0A1V8PRM4.A0A1V8PRM4_9BIFI.ABC.transporter. substrate.binding.protein.OS.Bifidobacterium.catenulatum.OX.16
tr.D1PG79.	tr.D1PG79.D1PG79_9BACT.Fumarate.reductase.succinate.dehydrogenase.flavoprotein.subunit.OS.Prevotella.copri. DSM.18205.OX.537011.GN.sdhA.PE.4.SV.1
tr.A0A369M4T5.	tr.A0A369M4T5.A0A369M4T5_EGGLN.Serine.threonine.protein.kinase.OS.Eggerthella.lenta.OX.84112.GN. C1853_16350.PE.3.SV.1
tr.WP_054704175.1.	tr.WP_054704175.1.WP_054704175.1_NCBI.pyruvate.ferredoxin..flavodoxin..oxidoreductase..Mediterraneibacter. glycyrrhizinilyticus..tr.WP_195609671.1.WP_195609671.1_NCBI.pyruvate.ferredoxin..flavodoxin..oxidoreductase.. Mediterraneibacter.glycyrrhizinilyticus..
tr.A0A3E4U7M2.	tr.A0A3E4U7M2.A0A3E4U7M2_9CLOT.L.fucose.isomerase.OS.Hungatella.hathewayi.OX.154046.GN.fucI.PE.3.SV.1.tr. WP_130789564.1.WP_130789564.1_NCBI.L.fucose.isomerase..Lachnoclostridium.pacaense..tr.A0A6P1Z546. A0A6P1Z546_9FIRM.L.fucose.isomerase.OS.Blautia.product
tr.WP_173767105.1.	tr.WP_173767105.1.WP_173767105.1_NCBI.MULTISPECIES..transcription.elongation.factor.GreA..Clostridiales..tr. WP_148461413.1.WP_148461413.1_NCBI.MULTISPECIES..transcription.elongation.factor.GreA..Clostridiales.
tr.A0A1V8PLT8.	tr.A0A1V8PLT8.A0A1V8PLT8_9BIFI.DNA.directed.RNA.polymerase.subunit.beta.OS.Bifidobacterium.catenulatum.OX. 1686.GN.rpoC.PE.3.SV.1.tr.A0A6L4V360.A0A6L4V360_9BIFI.DNA.directed.RNA.polymerase.subunit.beta.OS. Bifidobacterium.catenulatum.OX.1686.GN.rpoC.PE.3.S
tr.WP_119239543.1.	tr.WP_119239543.1.WP_119239543.1_NCBI.MULTISPECIES..chaperonin.GroEL..Clostridiales..tr.A0A564W1T1. A0A564W1T1_9FIRM.60.kDa.chaperonin.OS.Blautia.luti.OX.89014.GN.groL.PE.3.SV.1.tr.WP_173773732.1. WP_173773732.1_NCBI.chaperonin.GroEL..partial..Blautia.schink
tr.WP_008702995.1.	tr.WP_008702995.1.WP_008702995.1_NCBI.MULTISPECIES..sirohydrochlorin.cobaltochelatase..Clostridiales.
tr.A0A2Y9BLV7.	tr.A0A2Y9BLV7.A0A2Y9BLV7_9FIRM.Elongation.factor.Tu.OS.Faecalicatena.orotica.OX.1544.GN.tuf.PE.3.SV.1
tr.WP_025577282.1.	tr.WP_025577282.1.WP_025577282.1_NCBI.MULTISPECIES..phosphoglycerate.kinase..Clostridiales..tr. WP_173766836.1.WP_173766836.1_NCBI.phosphoglycerate.kinase..Blautia.glucerasea..tr.A0A4Q1RGS8. A0A4Q1RGS8_9FIRM.Phosphoglycerate.kinase.OS.Blautia.faecicola.OX.25
tr.WP_173773355.1.	tr.WP_173773355.1.WP_173773355.1_NCBI.MULTISPECIES..IMP.cyclohydrolase..Clostridiales..tr.WP_173718261.1. WP_173718261.1_NCBI.MULTISPECIES..IMP.cyclohydrolase..Clostridiales..tr.WP_148462774.1. WP_148462774.1_NCBI.MULTISPECIES..IMP.cyclohydrolase..Clostridia
tr.D4BRG5.	tr.D4BRG5.D4BRG5_BIFBR.ATPase.family.associated.with.various.cellular.activities..AAA..OS.Bifidobacterium.breve. DSM.20213...JCM.1192.OX.518634.GN.BIFBRE_04700.PE.3.SV.1.tr.A0A0M4MH70.A0A0M4MH70_BIFLI.ATP. dependent.Clp.protease.ATP.binding.subunit.OS.Bifido

Table S22. (continued)

Table S22B. (continued)

Abbreviation	Fasta Maxquant
tr.A6KXV7.	tr.A6KXV7.A6KXV7_BACV8.Putative.outer.membrane.protein..probably.involved.in.nutrient.binding.OS.Bacteroides.vulgatus..strain.ATCC.8482...DSM.1447...JCM.5826...NBRC.14291...NCTC.11154..OX.435590.GN.BVU_0563.PE.3.SV.1.tr.A0A7I0RSV7.A0A7I0RSV7_9BACT.Membrane
tr.A0A4P8KHU0.	tr.A0A4P8KHU0.A0A4P8KHU0_ENTAV.Glucose.6.phosphate.isomerase.OS.Enterococcus.avium.OX.33945.GN.pgi.PE.3.SV.1.tr.A0A2N8PV00.A0A2N8PV00_ENTAV.Glucose.6.phosphate.isomerase.OS.Enterococcus.avium.OX.33945.GN.pgi.PE.3.SV.1.tr.A0A1L8WTT4.A0A1L8WTT4_9ENTE.Glucose
tr.A0A3E3EA02.	tr.A0A3E3EA02.A0A3E3EA02_9FIRM.Elongation.factor.Tu.OS.Erysipelatoclostridium.ramosum.OX.1547.GN.tuf.PE.3.SV.1.tr.A0A3E3AC32.A0A3E3AC32_9FIRM.Elongation.factor.Tu.OS.Erysipelatoclostridium.ramosum.OX.1547.GN.tuf.PE.3.SV.1
tr.A0A564W8K3.	tr.A0A564W8K3.A0A564W8K3_9FIRM.Phosphoenolpyruvate.carboxykinase..ATP..OS.Blautia.luti.OX.89014.GN.pckA.PE.3.SV.1
tr.S0GNC3.	tr.S0GNC3.S0GNC3_9BACT.SusC.RagA.family.TonB.linked.outer.membrane.protein.OS.Parabacteroides.goldsteinii.dnLKV18.OX.1235789.GN.C803_04804.PE.3.SV.1
tr.WP_008703889.1.	tr.WP_008703889.1.WP_008703889.1_NCBI.MULTISPECIES..anaerobic.carbon.monoxide.dehydrogenase.catalytic.subunit..Clostridiales.
tr.A0A133LTJ9.	tr.A0A133LTJ9.A0A133LTJ9_BIFLN.Aspartate..tRNA.Asp.Asn..ligase.OS.Bifidobacterium.longum.OX.216816.GN.aspS.PE.3.SV.1.tr.A0A0M3T675.A0A0M3T675_BIFLI.Aspartate..tRNA.Asp.Asn..ligase.OS.Bifidobacterium.longum.subsp..infantis.OX.1682.GN.aspS.PE.3.SV.1.tr.E5XVW

Abbreviation	Proteins Ids
tr.WP_015525727.1.	WP_015525727.1;A5ZUI6;A0A564W402;WP_008707497.1;A0A1K1PRP2;A0A1H6I7Y0;A0A6P1ZBB0;A0A4Q1RH04;A0A1V4ID03
tr.A0A0A1GRX3	A0A0A1GRX3;A0A1S2VY79;A0A0M3T5H1;A0A126ST81;A0A315S068;KFI89168.1;WP_051912151.1;AUR34142.1
tr.WP_195360800.1.	WP_195360800.1
tr.WP_008705693.1.	WP_008705693.1;WP_173726912.1;WP_117854094.1;A0A4R4FB20;WP_070087401.1;D4MZD9;A0A6N7WM25;A5ZQR8;A0A413FLR6;A0A3E3I955;A0A2Y9CAV0;A0A1I0AVI8;A0A173XPM4;A0A6P1Z5F2;A0A1E3AC12;A0A413QC06;WP_173752049.1
tr.A0A5B3GKY1.	A0A5B3GKY1;WP_144365668.1
tr.A0A1L8SSH0.	A0A1L8SSH0
tr.A0A173R7M3.	A0A173R7M3;WP_070087926.1;Q0TMQ8;A0A2Y9BM50;A0A174LMQ2
tr.A5ZUS4.	A5ZUS4
tr.D4BNB9.	D4BNB9
tr.A0A1V8Q6G3.	A0A1V8Q6G3;A0A1V8PSH8;A0A087DLM1
tr.WP_022380221.1.	WP_022380221.1;WP_173765213.1;WP_173727273.1;WP_173755727.1;WP_173738584.1;A0A564W6C1
tr.D4MV01.	D4MV01
tr.D4BP81.	D4BP81;A0A0M3T6K3;A0A0A1GRJ0;WP_193531265.1;WP_033510160.1;KFI86509.1
tr.A0A087DNV3.	A0A087DNV3;A0A1V8PRM4;A0A126SV63;A0A6L9SKZ2;A0A1V8Q519
tr.D1PG79.	D1PG79;B3JG78;A0A413T419;E6K4X0;B5CU80;A0A137SYZ7
tr.A0A369M4T5.	A0A369M4T5
tr.WP_054704175.1.	WP_054704175.1;WP_195609671.1;A0A2Y9BFR0
tr.A0A3E4U7M2.	A0A3E4U7M2;WP_130789564.1;A0A6P1Z546;A0A3E2V1A8;A0A1I0H2P2;A0A174AWN2;A0A3T2UYP8;P69923;J7Q9D7;A0A174GZZ8;A0A6N7WCC6;A0A3E3I2Y0;A0A1E3AIP8;WP_117551297.1;WP_009267973.1;A0A2Y9BDS1
tr.WP_173767105.1.	WP_173767105.1;WP_148461413.1
tr.A0A1V8PLT8.	A0A1V8PLT8;A0A6L4V360;A0A1V8Q5E9;WP_051915779.1;WP_051912149.1;KFI89167.1;A0A4V1WJX7;A0A315S4P8;ABA19190.1
tr.WP_119239543.1.	WP_119239543.1;A0A564W1T1;WP_173773732.1;A5ZQR5;WP_006941416.1;G0VPW5;EFQ04625.1
tr.WP_008702995.1.	WP_008702995.1
tr.A0A2Y9BLV7.	A0A2Y9BLV7
tr.WP_025577282.1.	WP_025577282.1;WP_173766836.1;A0A4Q1RGS8;A0A6P1Z6X8;A0A5M8BN79;A0A413JMQ1
tr.WP_173773355.1.	WP_173773355.1;WP_173718261.1;WP_148462774.1;WP_130790115.1;D4LE66;A0A564W263;A0A413VNK4;A0A3E2WKL7;A0A3E2VGF2
tr.D4BRG5.	D4BRG5;A0A0M4MH70;A0A6L9SL86;A0A1V8Q3F6;A0A087DMK3;A0A126STI8
tr.A6KXV7.	A6KXV7;A0A7I0RSV7;A0A1Y3ZEA3;A6KXW2;A0A076IXI8;S0GLH8;S0GU45
tr.A0A4P8KHU0.	A0A4P8KHU0;A0A2N8PV00;A0A1L8WTT4
tr.A0A3E3EA02.	A0A3E3EA02;A0A3E3AC32
tr.A0A564W8K3.	A0A564W8K3
tr.S0GNC3.	S0GNC3

Table S22. (continued)

Table S22B. (continued)

Abbreviation	Proteins Ids
tr.WP_008703889.1.	WP_008703889.1
tr.A0A133LTJ9.	A0A133LTJ9;A0A0M3T675;E5XVW9;D4BRH4;A0A4S5BCB6;WP_033506522.1

Abbreviation	Protein names
tr.WP_015525727.1.	GGGtGRT protein [Clostridiales]; Uncharacterized protein - *Blautia obeum*; GGGtGRT protein - *Blautia Luti*; hypothetical protein [Clostridiales]; GGGtGRT protein - *Ruminococcus flavefaciens*; GGGtGRT protein - *Ruminococcus flavefaciens*; GGGtGRT protein - *Blautia producta*; GGGtGRT protein - *Blautia faecicola*; GGGtGRT protein - *Clostridium chromiireducens*
tr.A0A0A1GRX3	DNA-directed RNA polymerase subunit beta - *Bifidobacterium longum*; DNA-directed RNA polymerase subunit beta - *Bifidobacterium longum subsp. Infantis*; DNA-directed RNA polymerase subunit beta - *Bifidobacterium longum subsp. Infantis*; DNA-directed RNA polymerase subunit beta - *Bifidobacterium angulatum*; DNA-directed RNA polymerase subunit beta - *Bifidobacterium animalis subsp. lactis* (*Bifidobacterium lactis*); DNA-directed RNA polymerase subunit beta - *Bifidobacterium pullorum subsp. saeculare*; DNA-directed RNA polymerase subunit beta - *Bifidobacterium pullorum*; DNA-directed RNA polymerase subunit beta, partial - *Bifidobacterium pullorum subsp. saeculare*
tr.WP_195360800.1.	DUF5458 family protein [*Phocaeicola massiliensis*]
tr.WP_008705693.1.	IMP cyclohydrolase [Clostridiales]; IMP cyclohydrolase [*Blautia glucerasea*]; IMP cyclohydrolase [Clostridiales]; IMP cyclohydrolase - *Extibacter muris*; IMP cyclohydrolase [*Merdimonas faecis*]; IMP cyclohydrolase-like protein - *Anaerostipes hadrus*; IMP cyclohydrolase - *Eisenbergiella porci*; IMP cyclohydrolase-like protein - *Blautia obeum*; IMP cyclohydrolase - *Enterocloster asparagiformis*; IMP cyclohydrolase - *Eisenbergiella massiliensis*; IMP cyclohydrolase-like protein - *Faecalicatena orotica*; IMP cyclohydrolase - *Enterocloster lavalensis*; IMP cyclohydrolase - *Anaerostipes hadrus*; IMP cyclohydrolase - *Blautia producta*; IMP cyclohydrolase - *Eisenbergiella tayi*; IMP cyclohydrolase - *Ruminococcus bromii*; IMP cyclohydrolase [*Blautia*]
tr.A0A5B3GKY1.	Class II fructose-1,6-bisphosphate aldolase - *Anaerostipes hadrus*; Class II fructose-1,6-bisphosphate aldolase [*Lacrimispora amygdalina*]
tr.A0A1L8SSH0.	Glutamate dehydrogenase - *Enterococcus devriesei*
tr.A0A173R7M3.	50S ribosomal protein L5 - *Anaerostipes hadrus*; 50S ribosomal protein L5 [*Eubacteriales*]; 50S ribosomal protein L5 - *Clostridium perfringens*; 50S ribosomal protein L5 - *Faecalicatena orotica*; 50S ribosomal protein L5 - *Lachnospira pectinoschiza*
tr.A5ZUS4.	Putative carbamoyltransferase YgeW - *Blautia obeum*
tr.D4BNB9.	Carbamoyl-phosphate synthase large chain - *Bifidobacterium breve*
tr.A0A1V8Q6G3.	50S ribosomal protein L27 - *Bifidobacterium dentium*; 50S ribosomal protein L27 - *Bifidobacterium catenulatum*; 50S ribosomal protein L27 - *Bifidobacterium adolescentis*
tr.WP_022380221.1.	carbamoyl-phosphate synthase large subunit [Clostridiales]; carbamoyl-phosphate synthase large subunit [Clostridiales]; carbamoyl-phosphate synthase large subunit [*Blautia glucerasea*]; carbamoyl-phosphate synthase large subunit [*Blautia schinkii*]; carbamoyl-phosphate synthase large subunit [*Blautia schinkii*]; carbamoyl-phosphate synthase large chain - *Blautia luti*
tr.D4MV01.	NADH peroxidase - *Anaerostipes hadrus*
tr.D4BP81.	Chorismate synthase - *Bifidobacterium breve*; Chorismate synthase - *Bifidobacterium longum subsp. Infantis*; Chorismate synthase - *Bifidobacterium longum*; Chorismate synthase [*Bifidobacterium pullorum*]; Chorismate synthase [*Bifidobacterium pullorum*]; Chorismate synthase [*Bifidobacterium pullorum subsp. saeculare*]
tr.A0A087DNV3.	ABC transporter substrate-binding protein - *Bifidobacterium stercoris*; ABC transporter substrate-binding protein - *Bifidobacterium catenulatum*; ABC transporter substrate-binding protein - *Bifidobacterium angulatum*; ABC transporter substrate-binding protein - *Bifidobacterium dentium*; ABC transporter substrate-binding protein - *Bifidobacterium dentium*
tr.D1PG79.	Fumarate reductase/succinate dehydrogenase flavoprotein subunit - *Prevotella copri*; Succinate dehydrogenase or fumarate reductase, flavoprotein subunit - *Phocaeicola coprocola*; Fumarate reductase/succinate dehydrogenase flavoprotein subunit - *Phocaeicola coprophilus*; Succinate dehydrogenase or fumarate reductase, flavoprotein subunit - *Prevotella buccae*; Succinate dehydrogenase or fumarate reductase, flavoprotein subunit - *Phocaeicola plebeius*; Succinate dehydrogenase or fumarate reductase, flavoprotein subunit - *Prevotella bivia*
tr.A0A369M4T5.	Serine/threonine protein kinase - *Eggerthella lenta*
tr.WP_054704175.1.	pyruvate:ferredoxin (flavodoxin) oxidoreductase [*Mediterraneibacter glycyrrhizinilyticus*]; pyruvate:ferredoxin (flavodoxin) oxidoreductase [*Mediterraneibacter glycyrrhizinilyticus*]; pyruvate:ferredoxin oxidoreductase - *Faecalicatena orotica*

Table S22. (continued)

Table S22B. (continued)

Abbreviation	Protein names
tr.A0A3E4U7M2.	L-fucose isomerase - *Hungatella hathewayi*; L-fucose isomerase [*Lachnoclostridium pacaense*]; L-fucose isomerase - *Blautia producta*; L-fucose isomerase - *Enterocloster citroniae*; L-fucose isomerase - *Enterocloster lavalensis*; L-fucose isomerase - *Enterocloster clostridioformis*; L-fucose isomerase - *Shigella flexneri*; L-fucose isomerase - *Shigella flexneri*; L-fucose isomerase - *Escherichia coli*; L-fucose isomerase - *Clostridium disporicum*; L-fucose isomerase - *Eisenbergiella porci*; L-fucose isomerase - *Eisenbergiella massiliensis*; L-fucose isomerase - *Eisenbergiella tayi*; L-fucose isomerase [*Mediterraneibacter glycyrrhizinilyticus*]; L-fucose isomerase [*Mediterraneibacter glycyrrhizinilyticus*]; L-fucose isomerase - *Faecalicatena orotica*
tr.WP_173767105.1.	transcription elongation factor GreA [Clostridiales]; transcription elongation factor GreA [Clostridiales]
tr.A0A1V8PLT8.	DNA-directed RNA polymerase subunit beta - *Bifidobacterium catenulatum*; DNA-directed RNA polymerase subunit beta - *Bifidobacterium catenulatum*; DNA-directed RNA polymerase subunit beta - *Bifidobacterium dentium*; DNA-directed RNA polymerase subunit beta [*Bifidobacterium pullorum*]; DNA-directed RNA polymerase subunit beta [*Bifidobacterium pullorum*]; DNA-directed RNA polymerase subunit beta [*Bifidobacterium pullorum subsp. saeculare*]; DNA-directed RNA polymerase subunit beta - *Bifidobacterium animalis subsp. lactis* (*Bifidobacterium lactis*); DNA-directed RNA polymerase subunit beta - *Bifidobacterium animalis subsp. lactis* (*Bifidobacterium lactis*); DNA-directed RNA polymerase subunit beta, partial [*Bifidobacterium pullorum subsp. saeculare*
tr.WP_119239543.1.	chaperonin GroEL [Clostridiales]; 60 kDa chaperonin - *Blautia luti* ; chaperonin GroEL, partial [*Blautia schinkii*]; Chaperonin GroEL - *Blautia obeum*; chaperonin GroEL [*Megasphaera micronuciformis*]; Chaperonin GroEL - *Megasphaera elsdenii*; chaperonin GroEL [*Megasphaera micronuciformis*]
tr.WP_008702995.1.	sirohydrochlorin cobaltochelatase [Clostridiales]
tr.A0A2Y9BLV7.	Elongation factor Tu - *Faecalicatena orotica*
tr.WP_025577282.1.	phosphoglycerate kinase [Clostridiales]; phosphoglycerate kinase [*Blautia glucerasea*]; Phosphoglycerate kinase - *Blautia faecicola*; Phosphoglycerate kinase - *Blautia producta*; Phosphoglycerate kinase - *Clostridium symbiosum* (*Bacteroides symbiosus*); Phosphoglycerate kinase - *Clostridium symbiosum* (*Bacteroides symbiosus*)
tr.WP_173773355.1.	IMP cyclohydrolase [Clostridiales]; IMP cyclohydrolase [Clostridiales]; IMP cyclohydrolase [Eubacteriales]; IMP cyclohydrolase [*Lachnoclostridium pacaense*]; IMP cyclohydrolase-like protein - *Ruminococcus champanellensis*; IMP cyclohydrolase - *Blautia luti*; IMP cyclohydrolase - *Enterocloster aldenensis*; IMP cyclohydrolase - *Enterocloster aldenensis*; IMP cyclohydrolase-like protein - *Enterocloster citroniae*
tr.D4BRG5.	ATPase family associated with various cellular activities (AAA) - *Bifidobacterium breve*; ATP-dependent Clp protease ATP-binding subunit - *Bifidobacterium longum subsp. Infantis*; ATP-dependent Clp protease ATP-binding subunit - *Bifidobacterium dentium*; ATP-dependent Clp protease ATP-binding subunit - *Bifidobacterium dentium*; ATP-dependent Clp protease ATP-binding subunit - *Bifidobacterium adolescentis*; ATP-dependent Clp protease ATP-binding subunit ClpC - *Bifidobacterium angulatum*
tr.A6KXV7.	Putative outer membrane protein, probably involved in nutrient binding - *Bacteroides vulgatus*; Membrane protein - *Phocaeicola dorei*; SusC/RagA family TonB-linked outer membrane protein - *Phocaeicola dorei*; Putative outer membrane protein, probably involved in nutrient binding - *Phocaeicola vulgatus*; SusC/RagA family TonB-linked outer membrane protein - *Phocaeicola dorei*; SusC/RagA family TonB-linked outer membrane protein - *Parabacteroides goldsteinii*; SusC/RagA family TonB-linked outer membrane protein - *Parabacteroides goldsteinii*
tr.A0A4P8KHU0.	Glucose-6-phosphate isomerase - *Enterococcus avium*; Glucose-6-phosphate isomerase - *Enterococcus avium*; Glucose-6-phosphate isomerase - *Enterococcus raffinosus*
tr.A0A3E3EA02.	Elongation factor Tu - *Erysipelatoclostridium ramosum*; Elongation factor Tu - *Erysipelatoclostridium ramosum*
tr.A0A564W8K3.	Phosphoenolpyruvate carboxykinase (ATP) - *Blautia luti*
tr.S0GNC3.	SusC/RagA family TonB-linked outer membrane protein - *Parabacteroides goldsteinii*
tr.WP_008703889.1.	anaerobic carbon-monoxide dehydrogenase catalytic subunit [Clostridiales]
tr.A0A133LTJ9.	Aspartate--tRNA(Asp/Asn) ligase - *Bifidobacterium longum*; Aspartate--tRNA(Asp/Asn) ligase *Bifidobacterium longum subsp. Infantis*; Aspartate--tRNA(Asp/Asn) ligase - *Bifidobacterium longum*; Aspartate--tRNA(Asp/Asn) ligase - *Bifidobacterium breve*; Aspartate--tRNA(Asp/Asn) ligase - *Bifidobacterium longum subsp. infantis*; aspartate--tRNA ligase [*Bifidobacterium*]

Table S22. (continued)

Table S22C. clinical data 6M

set 1	set 2	set 3	set 4	set 5	mean
SCORADMAN	SCORADMAN	SCORADMAN	SCORADMAN	age	SCORADMAN
STOOLCONSIST	age	age	STOOLCOLOUR	num_inf	age
age	STOOLCOLOUR	STOOLCONSIST	age	SCORADMAN	STOOLCONSIST
num_inf	alrgyfat	STOOLFREQ	STOOLCONSIST	STOOLCONSIST	STOOLCOLOUR
alrgyfat	STOOLCONSIST	num_inf	num_inf	STOOLCOLOUR	num_inf
STOOLCOLOUR	SPTOWF	STOOLCOLOUR	alrgyfat	SPTOWF	alrgyfat
alrgymot	num_inf	delivery	alrgymot	sibl	STOOLFREQ
delivery	GASWIND	SPTOWF	GASWIND	STOOLFREQ	SPTOWF
SPTOSB	STOOLFREQ	GASWIND	STOOLFREQ	GASWIND	alrgymot
num_ab	treatment	alrgyfat	SPTOSB	alrgyfat	GASWIND

in top 10 for all 5 models

in top 10 for 4 models

Table S22D. immune data 6M

The abbreviations in the first table below are explained in the next table

set 1	set 2	set 3	set 4	set 5	mean
IL.1.alpha	X4E.BP1	CCL4	IL.1.alpha	X4E.BP1	IL.1.alpha
CXCL5	IL.1.alpha	IL.10RB	X4E.BP1	IL.1.alpha	X4E.BP1
PD.L1	TNFSF14	TNFSF14	LIF	CXCL5	CXCL5
IL.20RA	CCL20	MCP.4	MCP.4	LIF.R	CCL4
X4E.BP1	IL.15RA	CXCL5	CXCL5	PD.L1	PD.L1
EN.RAGE	CXCL5	IL.1.alpha	TGF.alpha	IL.12B	MCP.4
CCL4	TNFRSF9	EN.RAGE	OPG	CASP.8	EN.RAGE
CCL19	IL8	PD.L1	TNFRSF9	FGF.19	TNFSF14
TNFSF14	MMP.10	CCL3	CCL28	STAMBP	TNFRSF9
IL.15RA	CXCL10	X4E.BP1	CCL4	VEGFA	LIF

in top 10 for all 5 models

Abbreviations

variable	uniprot ID	name
IL.1.alpha	P01583	Interleukin-1 alpha (IL-1 alpha)
CXCL5	P42830	C-X-C motif chemokine 5 (CXCL5)
PD.L1	Q9NZQ7	Programmed cell death 1 ligand 1 (PD-L1)
IL.20RA	Q9UHF4	Interleukin-20 receptor subunit alpha (IL-20RA)
X4E.BP1	Q13541	Eukaryotic translation initiation factor 4E-binding protein 1 (4E-BP1)

Table S22. (continued)

Table S22D. (continued)

variable	uniprot ID	Name
EN.RAGE	P80511	Protein S100-A12 (EN-RAGE)
CCL4	P13236	C-C motif chemokine 4 (CCL4)
CCL19	Q99731	C-C motif chemokine 19 (CCL19)
TNFSF14	O43557	Tumor necrosis factor ligand superfamily member 14 (TNFSF14)
IL.15RA	Q13261	Interleukin-15 receptor subunit alpha (IL-15RA)
CCL20	P78556	C-C motif chemokine 20 (CCL20)
TNFRSF9	Q07011	Tumor necrosis factor receptor superfamily member 9 (TNFRSF9)
IL8	P10145	Interleukin-8 (IL-8)
MMP.10	P09238	Matrix metalloproteinase-10 (MMP-10)
CXCL10	P02778	C-X-C motif chemokine 10 (CXCL10)
IL.10RB	Q08334	Interleukin-10 receptor subunit beta (IL-10RB)
MCP.4	Q99616	Monocyte chemotactic protein 4 (MCP-4)
CCL3	P10147	C-C motif chemokine 3 (CCL3)
LIF	P15018	Leukemia inhibitory factor (LIF)
TGF.alpha	P01135	Transforming growth factor alpha (TGF-alpha)
OPG	O00300	Osteoprotegerin (OPG)
CCL28	Q9NRJ3	C-C motif chemokine 28 (CCL28)
LIF.R	P42702	Leukemia inhibitory factor receptor (LIF-R)
IL.12B	P29460	Interleukin-12 subunit beta (IL-12B)
CASP.8	Q14790	Caspase-8 (CASP-8)
FGF.19	O95750	Fibroblast growth factor 19 (FGF-19)
STAMBP	O95630	STAM-binding protein (STAMBP)
VEGFA	P15692	Vascular endothelial growth factor A (VEGF-A)

Table S22E. metabolomics – platform for polar and semi-polar metabolites – negative mode 6M

set 1	set 2	set 3	set 4	set 5	mean
Protocatechuic acid	Protocatechuic acid	Pyrocatechol	Pyrocatechol	Protocatechuic acid	Protocatechuic acid
Pyrocatechol	Pyrocatechol	Protocatechuic acid	Protocatechuic acid	Pyrocatechol	Pyrocatechol
myo-Inositol/ Galactose/ Fructose	trans-Aconitic acid	O-Acetylserine/ Glutamic acid	Syringic acid	Syringic acid	myo-Inositol/ Galactose/ Fructose
Phenylacetic acid	myo-Inositol/ Galactose/ Fructose	p-Hydroxyphenylacetic acid	3-Hydroxybutyric acid	myo-Inositol/ Galactose/ Fructose	Syringic acid
Gluconic acid	Argininosuccinic acid	myo-Inositol/ Galactose/ Fructose	2,5-Furandicarboxylic acid	4-Hydroxycinnamic acid	3-Hydroxybutyric acid
3-Methylxanthine/1-Methylxanthine/ 7-Methylxanthine	3-Methylxanthine/1-Methylxanthine/ 7-Methylxanthine	Mandelic acid	Xylulose	O-Acetylserine/ Glutamic acid	Phenylacetic acid
3-Hydroxybutyric acid	3-Hydroxybenzoic acid	Dimethylglycine	Argininosuccinic acid	Dimethylglycine	trans-Aconitic acid

Table S22. (continued)

Table S22E. (continued)

set 1	set 2	set 3	set 4	set 5	mean
N6-Carboxymethyllysine	FAD	Pyruvic acid	myo-Inositol/ Galactose/ Fructose	Gluconic acid	O-Acetylserine/ Glutamic acid
Syringic acid	Taurine	4-Hydroxybenzoic acid	Malic acid	3-Hydroxybutyric acid	Dimethylglycine
Oxoglutaric acid	1-Methyluric acid	Phenylacetic acid	N6-Carboxymethyllysine	4-Hydroxybenzoic acid	Gluconic acid

[yellow] in top 10 for all 5 models

Compound_name_reported	Compound_name_HMDB
Protocatechuic acid	Protocatechuic acid
Pyrocatechol	Pyrocatechol
myo-Inositol/ Galactose/ Fructose	myo-Inositol/ D-Galactose/ D-Fructose
Phenylacetic acid	Phenylacetic acid
Gluconic acid	Gluconic acid
3-Methylxanthine/1-Methylxanthine/ 7-Methylxanthine	3-Methylxanthine/1-Methylxanthine/ 7-Methylxanthine
3-Hydroxybutyric acid	3-Hydroxybutyric acid
N6-Carboxymethyllysine	N6-Carboxymethyllysine
Syringic acid	Syringic acid
Oxoglutaric acid	Oxoglutaric acid
trans-Aconitic acid	trans-Aconitic acid
Argininosuccinic acid	Argininosuccinic acid
3-Hydroxybenzoic acid	3-Hydroxybenzoic acid
FAD	FAD
Taurine	Taurine
1-Methyluric acid	1-Methyluric acid
O-Acetylserine/ Glutamic acid	O-Acetylserine/ Glutamic acid
p-Hydroxyphenylacetic acid	p-Hydroxyphenylacetic acid
Mandelic acid	Mandelic acid
Dimethylglycine	Dimethylglycine
Pyruvic acid	Pyruvic acid
4-Hydroxybenzoic acid	4-Hydroxybenzoic acid
2,5-Furandicarboxylic acid	2,5-Furandicarboxylic acid
Xylulose	D-Xylulose
Malic acid	Malic acid
4-Hydroxycinnamic acid	4-Hydroxycinnamic acid

Table S22. (continued)

Table S22F. metabolomics – platform for polar and semi-polar metabolites – positive mode 6M

set 1	set 2	set 3	set 4	set 5	mean
Guanidoacetic acid	N6,N6,N6-Trimethyllysine	N6,N6,N6-Trimethyllysine	N6,N6,N6-Trimethyllysine	N6,N6,N6-Trimethyllysine	N6,N6,N6-Trimethyllysine
Feature_mz_130.086	N1-Methyl-4-pyridone-3-carboxamide/Nudifloramide	Serotonin	Betaine	Beta-Guanidinopropionic acid	Citrulline
Citrulline	Aminoadipic acid	Citrulline	N1-Methyl-4-pyridone-3-carboxamide/Nudifloramide	Citrulline	N1-Methyl-4-pyridone-3-carboxamide/Nudifloramide
Beta-Guanidinopropionic acid	Citrulline	5-Hydroxytryptophan	Pyridoxal	Guanidoacetic acid	Guanidoacetic acid
5-Hydroxytryptophan	Dihydrouracil	Picolinic acid	Citrulline	N-Acetyltyrosine	Serotonin
Dodecanoylcarnitine	Betaine	Dihydrouracil	Dihydrouracil	Creatine	Feature_mz_130.086
N1-Methyl-4-pyridone-3-carboxamide/Nudifloramide	Dodecanoylcarnitine	Threonine/Homoserine	Guanidoacetic acid	Cytidine	Beta-Guanidinopropionic acid
Dihydrouracil	Feature_mz_130.086	Feature_mz_130.086	Dodecanoylcarnitine	N1-Methyl-4-pyridone-3-carboxamide/Nudifloramide	Dihydrouracil
Urocanic acid	Kynurenic acid	Beta-Guanidinopropionic acid	Picolinic acid	Dodecanoylcarnitine	Dodecanoylcarnitine
Cytidine	Serotonin	Dodecanoylcarnitine	5-Aminopentanoic acid	5-Hydroxytryptophan	Betaine

(yellow)	in top 10 for all 5 models
(light green)	in top 10 for 4 models

Compound_name_reported	Compound_name_HMDB
Guanidoacetic acid	Guanidoacetic acid
Feature_mz_130.086	unknown
Citrulline	Citrulline
Beta-Guanidinopropionic acid	Beta-Guanidinopropionic acid
5-Hydroxytryptophan	5-Hydroxy-L-tryptophan
Dodecanoylcarnitine	Dodecanoylcarnitine
N1-Methyl-4-pyridone-3-carboxamide/Nudifloramide	N1-Methyl-4-pyridone-3-carboxamide/Nudifloramide
Dihydrouracil	Dihydrouracil
Urocanic acid	Urocanic acid
Cytidine	Cytidine
N6,N6,N6-Trimethyllysine	N6,N6,N6-Trimethyl-L-lysine
Aminoadipic acid	Aminoadipic acid
Betaine	Betaine

Table S22. (continued)

Table S22F. (continued)

Compound_name_reported	Compound_name_HMDB
Kynurenic acid	Kynurenic acid
Serotonin	Serotonin
Picolinic acid	Picolinic acid
Threonine/Homoserine	L-Threonine/ L-Homoserine
Pyridoxal	Pyridoxal
5-Aminopentanoic acid	5-Aminopentanoic acid
N-Acetyltyrosine	N-Acetyl-L-tyrosine
Creatine	Creatine

Table S22G. metabolomics – platform for polar and semi-polar metabolites – positive mode 12M

set 1	set 2	set 3	set 4	set 5	mean
Cytidine	Ornithine	Citrulline	Serotonin	Feature_mz_130.086	Feature_mz_130.086
Threonine/Homoserine	Thymine	Feature_mz_130.0 86	Threonine/Homose rine	Citrulline	Citrulline
Sphinganine	Serotonin	N-Acetylcadaverine	Ethanolamine	Quinaldic acid	Ornithine
Feature_mz_130.086	Feature_mz_130.086	Serotonin	Pyridoxal	Ornithine	Threonine/Homoserin e
1-Methyladenosine/N6-Methyladenosine/2'-O-Methyladenosine	Citrulline	Cadaverine	Aspartic acid	Thymine	Serotonin
Deoxyguanosine	N-Acetylcadaverine	Targinine/Homoarg inine	Thymine	Threonine/Homoseri ne	Thymine
N6,N6,N6-Trimethyllysine	Pyridoxal	Threonine/Homose rine	Sphinganine	Targinine/Homoargin ine	Quinaldic acid
Citrulline	Cytosine	Ornithine	1-Methyladenosine/ N6-Methyladenosine/ 2'-O-Methyladenosine	Pipecolic acid	N-Acetylcadaverine
Carnitine	Ethanolamine	1-Methyladenosine/ N6-Methyladenosine/ 2'-O-Methyladenosine	Feature_mz_130.0 86	Cadaverine	Pyridoxal
Thiamine	Deoxyguanosine	5-Aminolevulinic acid/4-Hydroxyproline	Citrulline	1-Methyladenosine/N6 -Methyladenosine/2'-O-Methyladenosine	1-Methyladenosine/N6-Methyladenosine/2'-O-Methyladenosine

(yellow)	in top 10 for all 5 models
(light green)	in top 10 for 4 models

Table S22. (continued)

Table S22G. (continued)

**Compound_name_reported**	**Compound_name_HMDB**
Cytidine	Cytidine
Threonine/Homoserine	L-Threonine/ L-Homoserine
Sphinganine	Sphinganine
Feature_mz_130.086	unknown
1-Methyladenosine/N6-Methyladenosine/2'-O-Methyladenosine	1-Methyladenosine/N6-Methyladenosine/2'-O-Methyladenosine
Deoxyguanosine	Deoxyguanosine
N6,N6,N6-Trimethyllysine	N6,N6,N6-Trimethyl-L-lysine
Citrulline	Citrulline
Carnitine	L-Carnitine
Thiamine	Thiamine
Ornithine	Ornithine
Thymine	Thymine
Serotonin	Serotonin
N-Acetylcadaverine	N-Acetylcadaverine
Pyridoxal	Pyridoxal
Cytosine	Cytosine
Ethanolamine	Ethanolamine
Cadaverine	Cadaverine
Targinine/Homoarginine	L-Targinine/Homo-L-arginine
5-Aminolevulinic acid/4-Hydroxyproline	5-Aminolevulinic acid/4-Hydroxyproline
Aspartic acid	L-Aspartic acid
Quinaldic acid	Quinaldic acid
Pipecolic acid	Pipecolic acid

Table S23. Permutation-based variable importance (test sets). Features with permutation-based variable importance ≥ 0.01 for each view in the best model (Table S20) per train-test split, and the features with mean permutation-based variable importance ≥ 0.01.

Table S23A. 16S rRNA gene sequencing 0M

**set 1**	**set 2**	**set 3**	**set 4**	**set 5**	**mean**
Klebsiella	*Haemophilus*	*Klebsiella*	*Klebsiella*	*Klebsiella*	*Klebsiella*
Haemophilus	*Dialister*	*Haemophilus*	*Haemophilus*	*Haemophilus*	*Haemophilus*
Dialister	*Hungatella*	*Lachnoclostridium*	*Gemella*	*Lachnoclostridium*	*Gemella*
Hungatella	*Lachnoclostridium*	*Bacteroides*	*Dialister*	*Streptococcus*	*Dialister*
Lachnospiraceae_unclassified	*Bacteroides*	*Clostridium_sensu_stricto_1*	*Hungatella*	*Collinsella*	*Hungatella*
Erysipelatoclostridium	*TM7x*	*Lachnospiraceae_unclassified*	*Lachnoclostridium*		*Lachnoclostridium*
Robinsoniella	*Streptococcus*	*UBA1819*	*Bacteroides*		*Bacteroides*
	Erysipelatoclostridium		*Clostridium_sensu_stricto_1*		*Clostridium_sensu_stricto_1*
	Eisenbergiella		*Lachnospiraceae_unclassified*		*Lachnospiraceae_unclassified*
	Enterococcus		*TM7x*		*TM7x*
	Enterobacter		*Streptococcus*		*Streptococcus*
	Incertae_Sedis		*Eisenbergiella*		*Collinsella*
	Bifidobacterium		*Enterococcus*		*Erysipelatoclostridium*
			Enterobacteriaceae_unclassified		*Robinsoniella*
			Veillonella		
			Anaerostipes		
			Clostridioides		
			Dysgonomonas		
			Lachnospira		
			Citrobacter		
			Staphylococcus		
			Oscillospirales_ge		
			Terrisporobacter		
			Escherichia.Shigella		
			Clostridiaceae_unclassified		
			Blautia		
			Akkermansia		
			Fusicatenibacter		
			Subdoligranulum		
			Phascolarctobacterium		
			Eubacterium		

(yellow)	variable importance >= 0.01 for all 5 models
(light green)	variable importance >= 0.01 for 4 models

Table S23. (continued)

<u>Table S23B.</u> microbial proteomics 0M.

In the first table below, Fasta Maxquant for each protein group is abbreviated. For more details (full Fasta Maxquant, Protein Ids and protein names, see the next tables.

**set 1**	**set 2**	**set 3**	**set 4**	**set 5**	**mean**
tr.WP_008705693.1.	no features	tr.WP_008705693.1.	no features	tr.A0A087DM86.	tr.WP_008705693.1.
tr.A0A0A1GRX3.		tr.A0A0A1GRX3.		tr.WP_173755788.1.	tr.A0A0A1GRX3.
tr.A0A5B3GKY1.		tr.A0A5B3GKY1.		tr.A0A1V8PQF9.	tr.A0A5B3GKY1.
tr.WP_015525727.1.		tr.A0A173R7M3.			tr.WP_015525727.1.
tr.A0A173R7M3.		tr.A0A4V1NS38.			tr.A0A173R7M3.
tr.A0A4V1NS38.		tr.WP_025577282.1.			tr.A0A4V1NS38.
tr.WP_022380971.1.		tr.A0A3E3AD12.			
tr.WP_025577282.1.		tr.E5XX70.			
tr.A0A087DLM8.		tr.A0A133LTJ9.			
tr.A0A087DSI3.		tr.A0A173Z2F2.			
tr.WP_020993932.1.		tr.A0A1L8SSH0.			
tr.A0A1L8SSH0.		tr.A0A126SU96.			
tr.A0A0H2PPI2.		tr.A0A1V8Q6G3.			
tr.WP_008704844.1.		tr.WP_025579304.1.			
tr.A0A4R4FHH5.		tr.A5ZUS4.			
tr.WP_070089092.1.		tr.A0A1G5BPU3.			
tr.A0A6M4KU41.		tr.A0A4R4FHH5.			
tr.A0A174AKF1.		tr.WP_025578097.1.			
tr.D4BNB9.		tr.A0A0M4LTL3.			
tr.D4MZ58.		tr.A0A174US18.			
tr.WP_025578671.1.		tr.A6L1G5.			
tr.A0A6L4V7V3.		tr.A0A0M6WWS6.			
tr.A0A6P1YYM0.		tr.WP_097005984.1.			
tr.WP_081703234.1.		tr.A0A3E3ADB3.			
tr.WP_119239543.1.		tr.WP_009268670.1.			
tr.A0A2Y9BEA5.		tr.F3PHE4.			
tr.WP_147598065.1.		tr.A0A4S5B9L3.			
tr.WP_022380910.1.		tr.A7AIR2.			
tr.A0A0M6WQN5.		tr.WP_008703889.1.			
tr.WP_022380542.1.		tr.WP_195360800.1.			
tr.A0A0M6WNV3.		tr.D1PG79.			
tr.WP_022067013.1.		tr.A0A126SV93.			
tr.A0A0H2PPC5.		tr.A0A2Y9BGR4.			
tr.D4BS21.		tr.A0A0A1GQP2.			
tr.WP_025576924.1.		tr.WP_028254790.1.			
tr.WP_173755785.1.		tr.C7GD05.			
tr.WP_070087934.1.		tr.WP_156902361.1.			
		tr.WP_025577065.1.			
		tr.D4MV01.			
		tr.D4MZ60.			
		tr.A0A174D9I7.			
		tr.A6KXV7.			
		tr.A0A399IH89.			
		tr.A0A6P1YTK1.			
		tr.D4MUZ9.			
		tr.D4MXD7.			

Table S23. (continued)

Table S23B. (continued)

set 1	set 2	set 3	set 4	set 5	Mean
		tr.A0A087DMJ5.			
		tr.D4N113.			
		tr.D4BQK2.			
		tr.A0A1V8Q531.			
		tr.WP_025580805.1.			
		tr.D4MUG4.			
		tr.A0A133PIY4.			
		tr.WP_097006856.1.			
		tr.D4MYG8.			
		tr.C7G6S2.			
		tr.A0A2V2FBJ8			
		tr.WP_173767105.1.			
		tr.WP_173773355.1.			
		tr.WP_020993443.1.			
		tr.A0A6P1Z4H2.			
		tr.A0A6P1Z6F1.			
		tr.A0A4V2X185.			
		tr.D4MYK4.			
		tr.WP_147600180.1.			
		tr.A0A2Y9B7L8.			
		tr.A0A6P1Z1I4.			
		tr.WP_101723696.1.			
		tr.D4MZK6.			
		tr.A0A1V8PLT8.			
		tr.WP_027431932.1.			
		tr.D4MUH3.			
		tr.A6L1X3.			
		tr.D4MW58.			
		tr.A0A0M3T6V1.			
		tr.A0A0M4HKR2.			
		sp.P69780.			
		tr.A0A679HBI1.			
		tr.WP_033508353.1.			
		tr.A6L1G6.			
		tr.A0A0M4LFR2.			
		tr.A0A0H2PWI8.			
		tr.D4MW30.			
		tr.D4BLH5.			
		tr.A0A2U0C039.			
		tr.WP_195609585.1.			
		tr.A0A329TW61.			
		tr.A0A415MLA0.			
		tr.WP_119239650.1.			
		tr.A0A6P1YWQ6.			
		tr.A6KWT3.			
		tr.D4N0D1.			
		tr.WP_148461930.1.			
		tr.A0A1V8Q6B2.			
		tr.D4LD40.			
		tr.WP_005427721.1.			

Table S23. (continued)

Table S23B. (continued)

set 1	set 2	set 3	set 4	set 5	Mean
		tr.A6L1G4.			
		tr.E5XXC7.			
		tr.A0A0M6WFJ8.			
		tr.WP_142690962.1.			
		tr.D4MYK3.			
		tr.A6L7K3.			
		tr.WP_025577186.1.			
		tr.WP_148461524.1.			
		tr.WP_187565966.1.			
		tr.A0A2N0SUC3.			
		tr.A0A0H2P4J2.			
		tr.A0A151C3V8.			
		tr.A0A6N7WKN0.			
		tr.D4MZD8.			
		tr.D4N105.			
		tr.WP_070088499.1.			
		tr.WP_187565034.1.			
		tr.A0A0A1GN56.			
		tr.F0FPW5.			
		tr.D4BPL1			

Abbreviation	Fasta Maxquant
tr.WP_008705693.1.	tr.WP_008705693.1.WP_008705693.1_NCBI.MULTISPECIES..IMP.cyclohydrolase..Clostridiales..tr.WP_173726912.1.WP_173726912.1_NCBI.IMP.cyclohydrolase..Blautia.glucerasea..tr.WP_117854094.1.WP_117854094.1_NCBI.MULTISPECIES..IMP.cyclohydrolase..Clostridiales..tr.A
tr.A0A0A1GRX3.	tr.A0A0A1GRX3.A0A0A1GRX3_BIFLN.DNA.directed.RNA.polymerase.subunit.beta.OS.Bifidobacterium.longum.OX.216816.GN.rpoB.PE.3.SV.1.tr.A0A1S2VY79.A0A1S2VY79_BIFLI.DNA.directed.RNA.polymerase.subunit.beta.OS.Bifidobacterium.longum.subsp..infantis.OX.1682.GN.rpoB
tr.A0A5B3GKY1.	tr.A0A5B3GKY1.A0A5B3GKY1_ANAHA.Class.II.fructose.1.6.bisphosphate.aldolase.OS.Anaerostipes.hadrus.OX.649756.GN.fba.PE.4.SV.1.tr.WP_144365668.1.WP_144365668.1_NCBI.class.II.fructose.1.6.bisphosphate.aldolase..Lacrimispora.amygdalina.
tr.WP_015525727.1.	tr.WP_015525727.1.WP_015525727.1_NCBI.MULTISPECIES..GGGtGRT.protein..Clostridiales..tr.A5ZUI6.A5ZUI6_9FIRM.Uncharacterized.protein.OS.Blautia.obeum.ATCC.29174.OX.411459.GN.RUMOBE_02671.PE.4.SV.1.tr.A0A564W402.A0A564W402_9FIRM.Uncharacterized.protein.OS.Bla
tr.A0A173R7M3.	tr.A0A173R7M3.A0A173R7M3_ANAHA.50S.ribosomal.protein.L5.OS.Anaerostipes.hadrus.OX.649756.GN.rplE.PE.3.SV.1
tr.A0A4V1NS38.	tr.A0A4V1NS38.A0A4V1NS38_9FIRM.50S.ribosomal.protein.L16.OS.Blautia.faecicola.OX.2509240.GN.rplP.PE.3.SV.1.tr.A0A564W4Q7.A0A564W4Q7_9FIRM.50S.ribosomal.protein.L16.OS.Blautia.luti.OX.89014.GN.rplP.PE.3.SV.1.tr.A0A6P1Z5F6.A0A6P1Z5F6_9FIRM.50S.ribosomal.prot
tr.WP_022380971.1.	tr.WP_022380971.1.WP_022380971.1_NCBI.MULTISPECIES..glycine..tRNA.ligase..Clostridiales..tr.WP_173719039.1.WP_173719039.1_NCBI.MULTISPECIES..glycine..tRNA.ligase..Clostridiales..tr.WP_173735377.1.WP_173735377.1_NCBI.MULTISPECIES..glycine..tRNA.ligase..Clos
tr.WP_025577282.1.	tr.WP_025577282.1.WP_025577282.1_NCBI.MULTISPECIES..phosphoglycerate.kinase..Clostridiales..tr.WP_173766836.1.WP_173766836.1_NCBI.phosphoglycerate.kinase..Blautia.glucerasea..tr.A0A4Q1RGS8.A0A4Q1RGS8_9FIRM.Phosphoglycerate.kinase.OS.Blautia.faecicola.OX.25
tr.A0A087DLM8.	tr.A0A087DLM8.A0A087DLM8_BIFAD.50S.ribosomal.protein.L1.OS.Bifidobacterium.stercoris.JCM.15918.OX.1437612.GN.rplA.PE.3.SV.1.tr.A0A1V8Q5W3.A0A1V8Q5W3_9BIFI.50S.ribosomal.protein.L1.OS.Bifidobacterium.dentium.OX.1689.GN.rplA.PE.3.SV.1
tr.A0A087DSI3.	tr.A0A087DSI3.A0A087DSI3_BIFAD.Isocitrate.dehydrogenase..NADP..OS.Bifidobacterium.stercoris.JCM.15918.OX.1437612.GN.BSTER_1070.PE.3.SV.1.tr.A0A6L9SNF1.A0A6L9SNF1_9BIFI.Isocitrate.dehydrogenase..NADP..OS.Bifidobacterium.dentium.OX.1689.GN.F6S84_07505.PE.3.S
tr.WP_020993932.1.	tr.WP_020993932.1.WP_020993932.1_NCBI.MULTISPECIES..formate.C.acetyltransferase..Clostridiales.
tr.A0A1L8SSH0.	tr.A0A1L8SSH0.A0A1L8SSH0_9ENTE.Glutamate.dehydrogenase.OS.Enterococcus.devriesei.OX.319970.GN.RV00_GL003076.PE.3.SV.1

Table S23. (continued)

Table S23B. (continued)

Abbreviation	Fasta Maxquant
tr.A0A0H2PPI2.	tr.A0A0H2PPI2.A0A0H2PPI2_BIFBI.Calcium.transporting.ATPase.OS.Bifidobacterium.bifidum.OX.1681.GN.LMG11583_0518.PE.4.SV.1.tr.A0A0M5KVQ9.A0A0M5KVQ9_BIFBI.Cation.transporting.ATPase.PacL.OS.Bifidobacterium.bifidum.OX.1681.GN.RY70_19.PE.4.SV.1
tr.WP_008704844.1	tr.WP_008704844.1.WP_008704844.1_NCBI.MULTISPECIES..ketol.acid.reductoisomerase..Clostridiales.
tr.A0A4R4FHH5.	tr.A0A4R4FHH5.A0A4R4FHH5_9FIRM.Elongation.factor.G.OS.Extibacter.muris.OX.1796622.GN.E1963_00025.PE.3.SV.1.tr.A0A2Y9BND9.A0A2Y9BND9_9FIRM.Translation.elongation.factor.2..EF.2.EF.G..OS.Faecalicatena.orotica.OX.1544.GN.A8806_12244.PE.3.SV.1
tr.WP_070089092.1	tr.WP_070089092.1.WP_070089092.1_NCBI.type.I.glyceraldehyde.3.phosphate.dehydrogenase..Merdimonas.faecis.
tr.A0A6M4KU41.	tr.A0A6M4KU41.A0A6M4KU41_9BACT.Uncharacterized.protein.OS.Phocaeicola.dorei.OX.357276.GN.GN306_00415.PE.4.SV.1
tr.A0A174AKF1.	tr.A0A174AKF1.A0A174AKF1_9ACTN.Glyceraldehyde.3.phosphate.dehydrogenase.OS.Collinsella.aerofaciens.OX.74426.GN.gap.PE.3.SV.1.tr.WP_075844067.1.WP_075844067.1_NCBI.type.I.glyceraldehyde.3.phosphate.dehydrogenase..Collinsella.bouchesdurhonensis..tr.WP_040219
tr.D4BNB9.	tr.D4BNB9.D4BNB9_BIFBR.Carbamoyl.phosphate.synthase.large.chain.OS.Bifidobacterium.breve.DSM.20213...JCM.1192.OX.518634.GN.carB.PE.3.SV.1
tr.D4MZ58.	tr.D4MZ58.D4MZ58_ANAHA.Dihydropyrimidine.dehydrogenase.OS.Anaerostipes.hadrus.OX.649756.GN.gltD_2.PE.4.SV.1
tr.WP_025578671.1	tr.WP_025578671.1.WP_025578671.1_NCBI.MULTISPECIES..pyruvate.ferredoxin..flavodoxin..oxidoreductase..Clostridiales.
tr.A0A6L4V7V3.	tr.A0A6L4V7V3.A0A6L4V7V3_9BIFI.Alpha.1.4.glucan.phosphorylase.OS.Bifidobacterium.catenulatum.OX.1686.GN.GBA80_02370.PE.3.SV.1.tr.A0A1V8PT76.A0A1V8PT76_9BIFI.Alpha.1.4.glucan.phosphorylase.OS.Bifidobacterium.catenulatum.OX.1686.GN.B5782_0059.PE.3.SV.1.tr.A0
tr.A0A6P1YYM0.	tr.A0A6P1YYM0.A0A6P1YYM0_9FIRM.Class.II.fructose.1.6.bisphosphate.aldolase.OS.Blautia.producta.ATCC.27340...DSM.2950.OX.1121114.GN.fba.PE.4.SV.1
tr.WP_081703234.1	tr.WP_081703234.1.WP_081703234.1_NCBI.diaminopimelate.epimerase..Blautia.wexlerae.
tr.WP_119239543.1	tr.WP_119239543.1.WP_119239543.1_NCBI.MULTISPECIES..chaperonin.GroEL..Clostridiales..tr.A0A564W1T1.A0A564W1T1_9FIRM.60.kDa.chaperonin.OS.Blautia.luti.OX.89014.GN.groL.PE.3.SV.1.tr.WP_173773732.1.WP_173773732.1_NCBI.chaperonin.GroEL..partial..Blautia.schink
tr.A0A2Y9BEA5.	tr.A0A2Y9BEA5.A0A2Y9BEA5_9FIRM.Propionaldehyde.dehydrogenase.OS.Faecalicatena.orotica.OX.1544.GN.A8806_106101.PE.4.SV.1
tr.WP_147598065.1	tr.WP_147598065.1.WP_147598065.1_NCBI.phosphoenolpyruvate.carboxykinase..ATP...Blautia.caecimuris.
tr.WP_022380910.1	tr.WP_022380910.1.WP_022380910.1_NCBI.MULTISPECIES..adenylosuccinate.synthase..Clostridiales..tr.A0A173S6X6.A0A173S6X6_ANAHA.Adenylosuccinate.synthetase.OS.Anaerostipes.hadrus.OX.649756.GN.purA.PE.3.SV.1.tr.A0A6P1Z512.A0A6P1Z512_9FIRM.Adenylosuccinate.synt
tr.A0A0M6WQN5.	tr.A0A0M6WQN5.A0A0M6WQN5_9FIRM.Uncharacterized.protein.OS.Roseburia.faecis.OX.301302.GN.M72_30591.PE.4.SV.1
tr.WP_022380542.1	tr.WP_022380542.1.WP_022380542.1_NCBI.MULTISPECIES..single.stranded.DNA.binding.protein..Clostridiales..tr.WP_118583680.1.WP_118583680.1_NCBI.MULTISPECIES..single.stranded.DNA.binding.protein..Clostridiales..tr.WP_147600151.1.WP_147600151.1_NCBI.single.str
tr.A0A0M6WNV3.	tr.A0A0M6WNV3.A0A0M6WNV3_9FIRM.BIG2.domain.containing.protein.OS.Roseburia.faecis.OX.301302.GN.M72_28851.PE.4.SV.1
tr.WP_022067013.1	tr.WP_022067013.1.WP_022067013.1_NCBI.MULTISPECIES..glucosamine.6.phosphate.deaminase..Clostridiales..tr.A0A564VZ92.A0A564VZ92_9FIRM.Glucosamine.6.phosphate.deaminase.OS.Blautia.luti.OX.89014.GN.nagB_3.PE.3.SV.1.tr.WP_173736772.1.WP_173736772.1_NCBI.MULTIS
tr.A0A0H2PPC5.	tr.A0A0H2PPC5.A0A0H2PPC5_BIFBI.30S.ribosomal.protein.S1.OS.Bifidobacterium.bifidum.OX.1681.GN.B0085_1470.PE.4.SV.1.tr.A0A1V8Q3J5.A0A1V8Q3J5_9BIFI.30S.ribosomal.protein.S1.OS.Bifidobacterium.dentium.OX.1689.GN.B5790_1455.PE.4.SV.1.tr.A0A087DKB8.A0A087DKB8_B
tr.D4BS21.	tr.D4BS21.D4BS21_BIFBR.Glyceraldehyde.3.phosphate.dehydrogenase..type.I.OS.Bifidobacterium.breve.DSM.20213...JCM.1192.OX.518634.GN.gap.PE.3.SV.1.tr.A0A0M4N3H8.A0A0M4N3H8_BIFLN.Glyceraldehyde.3.phosphate.dehydrogenase.C.OS.Bifidobacterium.longum.OX.216816.G
tr.WP_025576924.1	tr.WP_025576924.1.WP_025576924.1_NCBI.phosphate.acetyltransferase..Blautia.wexlerae.
tr.WP_173755785.1	tr.WP_173755785.1.WP_173755785.1_NCBI.oxaloacetate.decarboxylase.subunit.alpha..Blautia.schinkii..tr.WP_
tr.WP_070087934.1	tr.WP_070087934.1.WP_070087934.1_NCBI.50S.ribosomal.protein.L2..Merdimonas.faecis..tr.WP_009267281.1.WP_009267281.1_NCBI.50S.ribosomal.protein.L2..Mediterraneibacter.glycyrrhizinilyticus..tr.A0A2Y9BH55.A0A2Y9BH55_9FIRM.50S.ribosomal.protein.L2.OS.Faecalica
tr.A0A3E3AD12.	tr.A0A3E3AD12.A0A3E3AD12_9FIRM.Carbohydrate.ABC.transporter.substrate.binding.protein.OS.Erysipelatoclostridium.ramosum.OX.1547.GN.DW242_12635.PE.4.SV.1.tr.A0A3E3EBV7.A0A3E3EBV7_9FIRM.Carbohydrate.ABC.transporter.substrate.binding.protein.OS.Erysipelatoclo

Table S23. (continued)

Table S23B. (continued)

Abbreviation	Fasta Maxquant
tr.E5XX70.	tr.E5XX70.E5XX70_BIFLN.UDP.glucose.4.epimerase.OS.Bifidobacterium.longum.OX.216816.GN.HMPREF0177_ 00573.PE.3.SV.1.tr.A0A0M5KUT4.A0A0M5KUT4_BIFLI.UDP.glucose.4.epimerase.OS.Bifidobacterium.longum. subsp..infantis.OX.1682.GN.RY67_265.PE.3.SV.1.tr.A0A0M4N4L2.A0
tr.A0A133LTJ9.	tr.A0A133LTJ9.A0A133LTJ9_BIFLN.Aspartate..tRNA.Asp.Asn..ligase.OS.Bifidobacterium.longum.OX.216816.GN. aspS.PE.3.SV.1.tr.A0A0M3T675.A0A0M3T675_BIFLI.Aspartate..tRNA.Asp.Asn..ligase.OS.Bifidobacterium.longum. subsp..infantis.OX.1682.GN.aspS.PE.3.SV.1.tr.E5XVW
tr.A0A173Z2F2.	tr.A0A173Z2F2.A0A173Z2F2_ANAHA.Elongation.factor.G.OS.Anaerostipes.hadrus.OX.649756.GN.fusA_1.PE.3. SV.1
tr.A0A126SU96.	tr.A0A126SU96.A0A126SU96_9BIFI.Ketol.acid.reductoisomerase..NADP.....OS.Bifidobacterium.angulatum.OX. 1683.GN.ilvC.PE.3.SV.1.tr.A0A1V8Q7P6.A0A1V8Q7P6_9BIFI.Ketol.acid.reductoisomerase..NADP.....OS. Bifidobacterium.dentium.OX.1689.GN.ilvC.PE.3.SV.1
tr.A0A1V8Q6G3.	tr.A0A1V8Q6G3.A0A1V8Q6G3_9BIFI.50S.ribosomal.protein.L27.OS.Bifidobacterium.dentium.OX.1689.GN.rpmA. PE.3.SV.1.tr.A0A1V8PSH8.A0A1V8PSH8_9BIFI.50S.ribosomal.protein.L27.OS.Bifidobacterium.catenulatum.OX. 1686.GN.rpmA.PE.3.SV.1.tr.A0A087DLM1.A0A087DLM1_BIFAD.5
tr.WP_025579304.1	tr.WP_025579304.1.WP_025579304.1_NCBI.MULTISPECIES..alcohol.dehydrogenase.catalytic.domain.containing. protein..Clostridiales..tr.A0A173SJ33.A0A173SJ33_9FIRM.Sorbitol.dehydrogenase.OS.Roseburia.faecis.OX.301302. GN.gutB.PE.3.SV.1.tr.A0A0M6WCQ4.A0A0M6WCQ4_9FI
tr.A5ZUS4	tr.A5ZUS4.A5ZUS4_9FIRM.Putative.carbamoyltransferase.YgeW.OS.Blautia.obeum.ATCC.29174.OX.411459.GN. ygeW.PE.3.SV.1
tr.A0A1G5BPU3.	tr.A0A1G5BPU3.A0A1G5BPU3_9FIRM.Ketol.acid.reductoisomerase..NADP.....OS.Ruminococcus.bromii.OX.40518. GN.ilvC.PE.3.SV.1
tr.WP_025578097.1	tr.WP_025578097.1.WP_025578097.1_NCBI.DUF4981.domain.containing.protein..Blautia.wexlerae.
tr.A0A0M4LTL3.	tr.A0A0M4LTL3.A0A0M4LTL3_BIFLI.Putative.ferredoxin.ferredoxin.NADP.reductase.OS.Bifidobacterium.longum. subsp..infantis.OX.1682.GN.RY67_776.PE.4.SV.1.tr.A0A1D7MR69.A0A1D7MR69_BIFLN.Ferredoxin..NADP. reductase.OS.Bifidobacterium.longum.OX.216816.GN.APC1462_00
tr.A0A174US18.	tr.A0A174US18.A0A174US18_9BACT.OmpA.family.protein.OS.Parabacteroides.distasonis.OX.823.GN.D7V78_ 07835.PE.4.SV.1
tr.A6L1G5.	tr.A6L1G5.A6L1G5_BACV8.Putative.outer.membrane.protein..probably.involved.in.nutrient.binding.OS. Bacteroides.vulgatus..strain.ATCC.8482...DSM.1447...JCM.5826...NBRC.14291...NCTC.11154..OX.435590.GN.BVU_ 1854.PE.3.SV.1.tr.A0A7I0QFN2.A0A7I0QFN2_9BACT.TonB.dep
tr.A0A0M6WWS6.	tr.A0A0M6WWS6.A0A0M6WWS6_9FIRM.Phosphoglycerate.kinase.OS.Roseburia.faecis.OX.301302.GN.pgk.PE.3. SV.1.tr.WP_144363565.1.WP_144363565.1_NCBI.phosphoglycerate.kinase..Lacrimispora.amygdalina..tr.G2T184. G2T184_ROSHA.Phosphoglycerate.kinase.OS.Roseburia.homini
tr.WP_097005984.1	tr.WP_097005984.1.WP_097005984.1_NCBI.elongation.factor.Ts..Lacrimispora.amygdalina..tr.A0A5M8BY86. A0A5M8BY86_CLOSY.Elongation.factor.Ts.OS.Clostridium.symbiosum.OX.1512.GN.tsf.PE.3.SV.1.tr.A0A174MDW4. A0A174MDW4_CLOSY.Elongation.factor.Ts.OS.Clostridium.sy
tr.A0A3E3ADB3.	tr.A0A3E3ADB3.A0A3E3ADB3_9FIRM.Phosphoenolpyruvate.protein.phosphotransferase.OS. Erysipelatoclostridium.ramosum.OX.1547.GN.ptsP.PE.3.SV.1
tr.WP_009268670.1	tr.WP_009268670.1.WP_009268670.1_NCBI.30S.ribosomal.protein.S2..Mediterraneibacter.glycyrrhizinilyticus..tr. WP_117551661.1.WP_117551661.1_NCBI.30S.ribosomal.protein.S2..Mediterraneibacter.glycyrrhizinilyticus..tr. C0FQI1.C0FQI1_9FIRM.30S.ribosomal.protein.S
tr.F3PHE4.	tr.F3PHE4.F3PHE4_9BACE.10.kDa.chaperonin.OS.Bacteroides.clarus.YIT.12056.OX.762984.GN.groS.PE.3.SV.1.tr. B0NQ83.B0NQ83_BACSE.10.kDa.chaperonin.OS.Bacteroides.stercoris.ATCC.43183.OX.449673.GN.groS.PE.3.SV.1. tr.A0A3E5GG32.A0A3E5GG32_9BACE.10.kDa.chaperonin.O
tr.A0A4S5B9L3.	tr.A0A4S5B9L3.A0A4S5B9L3_BIFLI.Extracellular.solute.binding.protein.OS.Bifidobacterium.longum.subsp..infantis. OX.1682.GN.E6L38_10710.PE.4.SV.1.tr.A0A087DNL3.A0A087DNL3_BIFAD.Family.1.extracellular.solute.binding. protein.OS.Bifidobacterium.stercoris.JCM.159
tr.A7AIR2.	tr.A7AIR2.A7AIR2_9BACT.Uncharacterized.protein.OS.Parabacteroides.merdae.ATCC.43184.OX.411477.GN. PARMER_03319.PE.4.SV.1.tr.A0A415PLT9.A0A415PLT9_9BACT.Uncharacterized.protein.OS.Parabacteroides. merdae.OX.46503.GN.DWZ81_01975.PE.4.SV.1
tr.WP_008703889.1	tr.WP_008703889.1.WP_008703889.1_NCBI.MULTISPECIES..anaerobic.carbon.monoxide.dehydrogenase.catalytic. subunit..Clostridiales.
tr.WP_195360800.1	tr.WP_195360800.1.WP_195360800.1_NCBI.DUF5458.family.protein..Phocaeicola.massiliensis.
tr.D1PG79.	tr.D1PG79.D1PG79_9BACT.Fumarate.reductase.succinate.dehydrogenase.flavoprotein.subunit.OS.Prevotella.copri. DSM.18205.OX.537011.GN.sdhA.PE.4.SV.1
tr.A0A126SV93.	tr.A0A126SV93.A0A126SV93_9BIFI.Antigen.84.OS.Bifidobacterium.angulatum.OX.1683.GN.Bang102_004950.PE.3. SV.1

Table S23. (continued)

Table S23B. (continued)

Abbreviation	Fasta Maxquant
tr.A0A2Y9BGR4.	tr.A0A2Y9BGR4.A0A2Y9BGR4_9FIRM.50S.ribosomal.protein.L19.OS.Faecalicatena.orotica.OX.1544.GN.rplS.PE.3.SV.1.tr.WP_158420463.1.WP_158420463.1_NCBI.50S.ribosomal.protein.L19..Blautia.glucerasea..tr.A0A4R4FHT1.A0A4R4FHT1_9FIRM.50S.ribosomal.protein.L19.OS.Ext
tr.A0A0A1GQP2.	tr.A0A0A1GQP2.A0A0A1GQP2_BIFLN.Glutamate.dehydrogenase.OS.Bifidobacterium.longum.OX.216816.GN.APC1462_0011.PE.3.SV.1.tr.A0A4S5BDT8.A0A4S5BDT8_BIFLI.Glutamate.dehydrogenase.OS.Bifidobacterium.longum.subsp..infantis.OX.1682.GN.E6L38_01430.PE.3.SV.1.tr.A0A0M4
tr.WP_028254790.1	tr.WP_028254790.1.WP_028254790.1_NCBI.methylmalonyl.CoA.mutase.family.protein..Veillonella.magna.
tr.C7GD05.	tr.C7GD05.C7GD05_9FIRM.50S.ribosomal.protein.L3.OS.Roseburia.intestinalis.L1.82.OX.536231.GN.rplC.PE.3.SV.1.tr.C0FTI0.C0FTI0_9FIRM.50S.ribosomal.protein.L3.OS.Roseburia.inulinivorans.DSM.16841.OX.622312.GN.rplC.PE3.SV.1.tr.WP_158421025.1.WP_158421025.1_NC
tr.WP_156902361.1	tr.WP_156902361.1.WP_156902361.1_NCBI.formate.C.acetyltransferase..Ruminococcus.callidus..tr.WP_117733070.1.WP_117733070.1_NCBI.MULTISPECIES..formate.C.acetyltransferase..Ruminococcus..tr.ERJ93286.1.ERJ93286.1_NCBI.formate.C.acetyltransferase..Ruminococcus
tr.WP_025577065.1	tr.WP_025577065.1.WP_025577065.1_NCBI.MULTISPECIES..acetyl.CoA.decarbonylase.synthase.complex.subunit.gamma..Clostridiales..tr.WP_021926204.1.WP_021926204.1_NCBI.MULTISPECIES..acetyl.CoA.decarbonylase.synthase.complex.subunit.gamma..Clostridiales.
tr.D4MV01.	tr.D4MV01.D4MV01_ANAHA.NADH.peroxidase.OS.Anaerostipes.hadrus.OX.649756.GN.rbr3A.PE.4.SV.1
tr.D4MZ60.	tr.D4MZ60.D4MZ60_ANAHA.Inosine.5.monophosphate.dehydrogenase.OS.Anaerostipes.hadrus.OX.649756.GN.guaB.PE.3.SV.1.tr.G2T2F1.G2T2F1_ROSHA.Inosine.5.monophosphate.dehydrogenase.OS.Roseburia.hominis..strain.DSM.16839...JCM.17582...NCIMB.14029...A2.183..OX.585
tr.A0A174D9I7.	tr.A0A174D9I7.A0A174D9I7_ANAHA.Arginine.binding.extracellular.protein.ArtP.OS.Anaerostipes.hadrus.OX.649756.GN.artP_2.PE.4.SV.1
tr.A6KXV7.	tr.A6KXV7.A6KXV7_BACV8.Putative.outer.membrane.protein..probably.involved.in.nutrient.binding.OS.Bacteroides.vulgatus..strain.ATCC.8482...DSM.1447...JCM.5826...NBRC.14291...NCTC.11154..OX.435590.GN.BVU_0563.PE.3.SV.1.tr.A0A7I0RSV7.A0A7I0RSV7_9BACT.Membrane
tr.A0A399IH89.	tr.A0A399IH89.A0A399IH89_9CLOT.Probable.transaldolase.OS.Clostridium.chromiireducens.OX.225345.GN.fsa.PE.3.SV.1.tr.A0A1V4IW50.A0A1V4IW50_9CLOT.Probable.transaldolase.OS.Clostridium.chromiireducens.OX.225345.GN.tal_1.PE.3.SV.1.tr.A0A1V4IEV4.A0A1V4IEV4_9CLOT
tr.A0A6P1YTK1.	tr.A0A6P1YTK1.A0A6P1YTK1_9FIRM.Acetyl.CoA.decarbonylase.synthase.complex.subunit.delta.OS.Blautia.producta.ATCC.27340...DSM.2950.OX.1121114.GN.GXM18_01135.PE.4.SV.1.tr.A0A4Q1RH58.A0A4Q1RH58_9FIRM.Acetyl.CoA.decarbonylase.synthase.complex.subunit.delta.OS.B
tr.D4MUZ9.	tr.D4MUZ9.D4MUZ9_ANAHA.FAD.FMN.containing.dehydrogenases.OS.Anaerostipes.hadrus.OX.649756.GN.CL2_23560.PE.4.SV.1.tr.A0A5B3G355.A0A5B3G355_ANAHA.FAD.binding.oxidoreductase.OS.Anaerostipes.hadrus.OX.649756.GN.F2Y14_13780.PE.4.SV.1
tr.D4MXD7.	tr.D4MXD7.D4MXD7_ANAHA.Glutamate.dehydrogenase.OS.Anaerostipes.hadrus.OX.649756.GN.gdhA.PE.3.SV.1
tr.A0A087DMJ5.	tr.A0A087DMJ5.A0A087DMJ5_BIFAD.60.kDa.chaperonin.OS.Bifidobacterium.stercoris.JCM.15918.OX.1437612.GN.groL.PE.3.SV.1
tr.D4N113.	tr.D4N113.D4N113_ANAHA.Inosose.isomerase.OS.Anaerostipes.hadrus.OX.649756.GN.iolI.PE.4.SV.1.tr.A0A174NB50.A0A174NB50_ANAHA.Inosose.isomerase.OS.Anaerostipes.hadrus.OX.649756.GN.iolI.PE.4.SV.1.tr.WP_173755379.1.WP_173755379.1_NCBI.MULTISPECIES..TIM.barrel.p
tr.D4BQK2.	tr.D4BQK2.D4BQK2_BIFBR.Phosphate.binding.protein.PstS.OS.Bifidobacterium.breve.DSM.20213...JCM.1192.OX.518634.GN.pstS.PE.3.SV.1
tr.A0A1V8Q531.	tr.A0A1V8Q531.A0A1V8Q531_9BIFI.50S.ribosomal.protein.L2.OS.Bifidobacterium.dentium.OX.1689.GN.rplB.PE.3.SV.1.tr.A0A087DLV3.A0A087DLV3_BIFAD.50S.ribosomal.protein.L2.OS.Bifidobacterium.stercoris.JCM.15918.OX.1437612.GN.rplB.PE.3.SV.1.tr.WP_033505254.1.WP_03
tr.WP_025580805.1	tr.WP_025580805.1.WP_025580805.1_NCBI.ATP.dependent.chaperone.ClpB..Blautia.wexlerae.
tr.D4MUG4.	tr.D4MUG4.D4MUG4_ANAHA.50S.ribosomal.protein.L7.L12.OS.Anaerostipes.hadrus.OX.649756.GN.rplL.PE.3.SV.1
tr.A0A133PIY4.	tr.A0A133PIY4.A0A133PIY4_LACGS.30S.ribosomal.protein.S2.OS.Lactobacillus.gasseri.OX.1596.GN.rpsB.PE.3.SV.1
tr.WP_097006856.1	tr.WP_097006856.1.WP_097006856.1_NCBI.sn.glycerol.3.phosphate.ABC.transporter.ATP.binding.protein.UgpC..Lacrimispora.amygdalina..tr.A0A174KBQ7.A0A174KBQ7_9FIRM.ABC.transporter.OS.Enterocloster.clostridioformis.OX.1531.GN.ugpC_3.PE.4.SV.1.tr.WP_027431919.1.
tr.D4MYG8.	tr.D4MYG8.D4MYG8_ANAHA.50S.ribosomal.protein.L2.OS.Anaerostipes.hadrus.OX.649756.GN.rplB.PE.3.SV.1
tr.C7G6S2.	tr.C7G6S2.C7G6S2_9FIRM.50S.ribosomal.protein.L7.L12.OS.Roseburia.intestinalis.L1.82.OX.536231.GN.rplL.PE.3.SV.1
tr.A0A2V2FBJ8.	tr.A0A2V2FBJ8.A0A2V2FBJ8_9FIRM.Phosphoglycerate.kinase.OS.Subdoligranulum.variabile.OX.214851.GN.pgk.PE.3.SV.1.tr.A0A1T4X822.A0A1T4X822_9FIRM.Phosphoglycerate.kinase.OS.Gemmiger.formicilis.OX.745368.GN.pgk.PE.3.SV.1
tr.WP_173767105.1	tr.WP_173767105.1.WP_173767105.1_NCBI.MULTISPECIES..transcription.elongation.factor.GreA..Clostridiales..tr.WP_148461413.1.WP_148461413.1_NCBI.MULTISPECIES..transcription.elongation.factor.GreA..Clostridiales.

Table S23. (continued)

Table S23B. (continued)

Abbreviation	Fasta Maxquant
tr.WP_173773355.1	tr.WP_173773355.1.WP_173773355.1_NCBI.MULTISPECIES..IMP.cyclohydrolase..Clostridiales..tr.WP_173718261.1.WP_173718261.1_NCBI.MULTISPECIES..IMP.cyclohydrolase..Clostridiales..tr.WP_148462774.1.WP_148462774.1_NCBI.MULTISPECIES..IMP.cyclohydrolase..Clostridia
tr.WP_020993443.1	tr.WP_020993443.1.WP_020993443.1_NCBI.MULTISPECIES..30S.ribosomal.protein.S6..Clostridiales..tr.WP_158420085.1.WP_158420085.1_NCBI.30S.ribosomal.protein.S6..Blautia.glucerasea..tr.WP_117851485.1.WP_117851485.1_NCBI.MULTISPECIES..30S.ribosomal.protein.S6..C
tr.A0A6P1Z4H2	tr.A0A6P1Z4H2.A0A6P1Z4H2_9FIRM.50S.ribosomal.protein.L5.OS.Blautia.producta.ATCC.27340...DSM.2950.OX.1121114.GN.rplE.PE.3.SV.1.tr.A0A174GFP3.A0A174GFP3_CLOSY.50S.ribosomal.protein.L5.OS.Clostridium.symbiosum.OX.1512.GN.rplE.PE.3.SV.1.tr.A0A0M6X1M0.A0A0M6X1
tr.A0A6P1Z6F1	tr.A0A6P1Z6F1.A0A6P1Z6F1_9FIRM.Pyruvate..phosphate.dikinase.OS.Blautia.producta.ATCC.27340...DSM.2950.OX.1121114.GN.ppdK.PE.3.SV.1
tr.A0A4V2X185	tr.A0A4V2X185.A0A4V2X185_9BACT.SusC.RagA.family.TonB.linked.outer.membrane.protein.OS.Phocaeicola.dorei.OX.357276.GN.EL88_18780.PE.3.SV.1.tr.A6L1G3.A6L1G3_BACV8.Putative.outer.membrane.protein..probably.involved.in.nutrient.binding.OS.Bacteroides.vulgatus
tr.D4MYK4	tr.D4MYK4.D4MYK4_ANAHA.4Fe.4S.dicluster.domain.containing.protein.OS.Anaerostipes.hadrus.OX.649756.GN.CL2_06520.PE.4.SV.1
tr.WP_147600180.1	tr.WP_147600180.1.WP_147600180.1_NCBI.pyruvate..phosphate.dikinase..Blautia.caecimuris.
tr.A0A2Y9B7L8	tr.A0A2Y9B7L8.A0A2Y9B7L8_9FIRM.Nitrogen.fixation.NifU.like.protein.OS.Faecalicatena.orotica.OX.1544.GN.A8806_10120.PE.4.SV.1.tr.D4MZ07.D4MZ07_ANAHA.Fe.S.cluster.assembly.scaffold.protein.NifU.OS.Anaerostipes.hadrus.OX.649756.GN.nifU.PE.4.SV.1.tr.A5ZPB7.A5Z
tr.A0A6P1Z1I4	tr.A0A6P1Z1I4.A0A6P1Z1I4_9FIRM.sn.glycerol.3.phosphate.ABC.transporter.ATP.binding.protein.UgpC.OS.Blautia.producta.ATCC.27340...DSM.2950.OX.1121114.GN.ugpC.PE.4.SV.1
tr.WP_101723696.1	tr.WP_101723696.1.WP_101723696.1_NCBI.IMP.dehydrogenase..Eggerthella.timonensis..tr.A0A369N8X2.A0A369N8X2_EGGLN.IMP.dehydrogenase.OS.Eggerthella.lenta.OX.84112.GN.C1853_03090.PE.3.SV.1
tr.D4MZK6	tr.D4MZK6.D4MZK6_ANAHA.Enoyl.CoA.hydratase.OS.Anaerostipes.hadrus.OX.649756.GN.echA8_1.PE.3.SV.1
tr.A0A1V8PLT8	tr.A0A1V8PLT8.A0A1V8PLT8_9BIFI.DNA.directed.RNA.polymerase.subunit.beta.OS.Bifidobacterium.catenulatum.OX.1686.GN.rpoC.PE.3.SV.1.tr.A0A6L4V360.A0A6L4V360_9BIFI.DNA.directed.RNA.polymerase.subunit.beta.OS.Bifidobacterium.catenulatum.OX.1686.GN.rpoC.PE.3.S
tr.WP_027431932.1	tr.WP_027431932.1.WP_027431932.1_NCBI.MULTISPECIES..class.II.fructose.1.6.bisphosphate.aldolase..Lachnospira..tr.A0A1G9SX47.A0A1G9SX47_9FIRM.Fructose.bisphosphate.aldolase..class.II.OS.Lachnospira.pectinoschiza.OX.28052.GN.SAMN05216544_0108.PE.4.SV.1
tr.D4MUH3	tr.D4MUH3.D4MUH3_ANAHA.DNA.directed.RNA.polymerase.subunit.beta.OS.Anaerostipes.hadrus.OX.649756.GN.rpoB.PE.3.SV.1.tr.A0A5B3GDA7.A0A5B3GDA7_ANAHA.DNA.directed.RNA.polymerase.subunit.beta.OS.Anaerostipes.hadrus.OX.649756.GN.rpoB.PE.3.SV.1
tr.A6L1X3	tr.A6L1X3.A6L1X3_BACV8.Putative.outer.membrane.protein..probably.involved.in.nutrient.binding.OS.Bacteroides.vulgatus..strain.ATCC.8482...DSM.1447...JCM.5826...NBRC.14291...NCTC.11154..OX.435590.GN.BVU_2020.PE.3.SV.1.tr.A0A6M4L9D4.A0A6M4L9D4_9BACT.SusC.Rag
tr.D4MW58	tr.D4MW58.D4MW58_ANAHA.Phosphate.ABC.transporter.substrate.binding.protein.OS.Anaerostipes.hadrus.OX.649756.GN.pstS.PE.4.SV.1.tr.A0A5B3GIV9.A0A5B3GIV9_ANAHA.Phosphate.ABC.transporter.substrate.binding.protein.OS.Anaerostipes.hadrus.OX.649756.GN.F2Y14_03560
tr.A0A0M3T6V1	tr.A0A0M3T6V1.A0A0M3T6V1_BIFLI.1.4.alpha.glucan.branching.enzyme.GlgB.OS.Bifidobacterium.longum.subsp..infantis.OX.1682.GN.glgB.PE.3.SV.1.tr.A0A4S5BHN7.A0A4S5BHN7_BIFLI.1.4.alpha.glucan.branching.enzyme.GlgB.OS.Bifidobacterium.longum.subsp..infantis.OX.168
tr.A0A0M4HKR2	tr.A0A0M4HKR2.A0A0M4HKR2_STRTR.Enolase.OS.Streptococcus.thermophilus.OX.1308.GN.eno.PE.3.SV.1.tr.V5QQV9.V5QQV9_STRSL.Phosphopyruvate.hydratase..Fragment..OS.Streptococcus.salivarius.OX.1304.GN.eno.PE.3.SV.1.tr.F8DIT1.F8DIT1_STREP.Enolase.OS.Streptococcus.p
sp.P69780	sp.P69780.LPP_SHIFL.Major.outer.membrane.lipoprotein.Lpp.OS.Shigella.flexneri.OX.623.GN.lpp.PE.3.SV.1.tr.C3T887.C3T887_ECOLX.Major.outer.membrane.lipoprotein.Lpp.OS.Escherichia.coli.OX.562.GN.lpp.PE.3.SV.1.tr.B7LQ99.B7LQ99_ESCF3.Major.outer.membrane.lipopr
tr.A0A679HBI1	tr.A0A679HBI1.A0A679HBI1_BACT4.Superoxide.dismutase.OS.Bacteroides.thetaiotaomicron.OX.818.GN.BatF92_23820.PE.3.SV.1.tr.D6D5K9.D6D5K9_9BACE.Superoxide.dismutase.OS.Bacteroides.xylanisolvens.XB1A.OX.657309.GN.BXY_02250.PE.3.SV.1.tr.A0A0K6BVG6.A0A0K6BVG6_BAC
tr.WP_033508353.1	tr.WP_033508353.1.WP_033508353.1_NCBI.MULTISPECIES..30S.ribosomal.protein.S10..Bifidobacterium..tr.WP_003827292.1.WP_003827292.1_NCBI.MULTISPECIES..30S.ribosomal.protein.S10..Terrabacteria.group..tr.KFI88792.1.KFI88792.1_NCBI.30S.ribosomal.protein.S10..Bif
tr.A6L1G6	tr.A6L1G6.A6L1G6_BACV8.Putative.outer.membrane.protein..probably.involved.in.nutrient.binding.OS.Bacteroides.vulgatus..strain.ATCC.8482...DSM.1447...JCM.5826...NBRC.14291...NCTC.11154..OX.435590.GN.BVU_1855.PE.3.SV.1.tr.A0A076IY48.A0A076IY48_9BACT.RagB.Sus

Table S23. (continued)

Table S23B. (continued)

Abbreviation	Fasta Maxquant
tr.A0A0M4LFR2	tr.A0A0M4LFR2.A0A0M4LFR2_BIFLI.30S.ribosomal.protein.S13.OS.Bifidobacterium.longum.subsp..infantis.OX.1682.GN.rpsM.PE.3.SV.1.tr.A0A0A1GPP2.A0A0A1GPP2_BIFLN.30S.ribosomal.protein.S13.OS.Bifidobacterium.longum.OX.216816.GN.rpsM.PE.3.SV.1.tr.D4BQT5.D4BQT5_BIF
tr.A0A0H2PWI8	tr.A0A0H2PWI8.A0A0H2PWI8_BIFBI.Trigger.factor.OS.Bifidobacterium.bifidum.OX.1681.GN.tig.PE.3.SV.1.tr.A0A0M4LZ74.A0A0M4LZ74_BIFBI.Trigger.factor.OS.Bifidobacterium.bifidum.OX.1681.GN.tig.PE.3.SV.1
tr.D4MW30	tr.D4MW30.D4MW30_ANAHA.Bacterial.Ig.like.domain..Group.2..OS.Anaerostipes.hadrus.OX.649756.GN.CL2_27890.PE.4.SV.1.tr.A0A1Q2C8D3.A0A1Q2C8D3_ANAHA.Uncharacterized.protein.OS.Anaerostipes.hadrus.OX.649756.GN.DO83_10630.PE.4.SV.1
tr.D4BLH5	tr.D4BLH5.D4BLH5_BIFBR.Alkyl.hydroperoxide.reductase.C.OS.Bifidobacterium.breve.DSM.20213...JCM.1192.OX.518634.GN.ahpC.PE.3.SV.1.tr.A0A1V8PLA3.A0A1V8PLA3_9BIFI.Alkyl.hydroperoxide.reductase.C.OS.Bifidobacterium.catenulatum.OX.1686.GN.ahpC.PE.3.SV.1
tr.A0A2U0C039	tr.A0A2U0C039.A0A2U0C039_BIFBI.30S.ribosomal.protein.S3.OS.Bifidobacterium.bifidum.OX.1681.GN.rpsC.PE.3.SV.1
tr.WP_195609585.1	tr.WP_195609585.1.WP_195609585.1_NCBI.glutamine.synthetase.III..Mediterraneibacter.glycyrrhizinilyticus..tr.A0A2Y9CAG1.A0A2Y9CAG1_9FIRM.Glutamine.synthetase.OS.Faecalicatena.orotica.OX.1544.GN.A8806_11218.PE.3.SV.1
tr.A0A329TW61	tr.A0A329TW61.A0A329TW61_9FIRM.Uronate.isomerase.OS.Faecalibacterium.prausnitzii.OX.853.GN.uxaC.PE.3.SV.1
tr.A0A415MLA0	tr.A0A415MLA0.A0A415MLA0_9BACT.Uncharacterized.protein.OS.Parabacteroides.distasonis.OX.823.GN.DW002_11805.PE.4.SV.1
tr.WP_119239650.1	tr.WP_119239650.1.WP_119239650.1_NCBI.MULTISPECIES..2.3.bisphosphoglycerate.independent.phosphoglycerate.mutase..Clostridiales..tr.A0A564W6U6.A0A564W6U6_9FIRM.2.3.bisphosphoglycerate.independent.phosphoglycerate.mutase.OS.Blautia.luti.OX.89014.GN.gpmI.PE.3
tr.A0A6P1YWQ6	tr.A0A6P1YWQ6.A0A6P1YWQ6_9FIRM.Phosphoenolpyruvate.carboxykinase..ATP..OS.Blautia.producta.ATCC.27340...DSM.2950.OX.1121114.GN.GXM18_05225.PE.3.SV.1.tr.A0A2Y9BK62.A0A2Y9BK62_9FIRM.Phosphoenolpyruvate.carboxykinase..ATP..OS.Faecalicatena.orotica.OX.1544.GN.
tr.A6KWT3	tr.A6KWT3.A6KWT3_BACV8.Putative.outer.membrane.protein..probably.involved.in.nutrient.binding.OS.Bacteroides.vulgatus..strain.ATCC.8482...DSM.1447...JCM.5826...NBRC.14291...NCTC.11154..OX.435590.GN.BVU_0169.PE.4.SV.1
tr.D4N0D1	tr.D4N0D1.D4N0D1_ANAHA.5.methyltetrahydropteroyltriglutamate..homocysteine.S.methyltransferase.OS.Anaerostipes.hadrus.OX.649756.GN.CL2_13700.PE.4.SV.1.tr.A0A412UNN9.A0A412UNN9_9FIRM.5.methyltetrahydropteroyltriglutamate..homocysteine.S.methyltransferase.OS
tr.WP_148461930.1	tr.WP_148461930.1.WP_148461930.1_NCBI.MULTISPECIES..phosphoenolpyruvate.carboxykinase..ATP...Clostridiales.
tr.A0A1V8Q6B2	tr.A0A1V8Q6B2.A0A1V8Q6B2_9BIFI.30S.ribosomal.protein.S15.OS.Bifidobacterium.dentium.OX.1689.GN.rpsO.PE.3.SV.1.tr.A0A1V8PS80.A0A1V8PS80_9BIFI.30S.ribosomal.protein.S15.OS.Bifidobacterium.catenulatum.OX.1686.GN.rpsO.PE.3.SV.1.tr.A0A087DLN9.A0A087DLN9_BIFAD.3
tr.D4LD40	tr.D4LD40.D4LD40_RUMC1.Sulfide.dehydrogenase..Flavoprotein..subunit.SudA.OS.Ruminococcus.champanellensis..strain.DSM.18848...JCM.17042...KCTC.15320...18P13..OX.213810.GN.RUM_14260.PE.4.SV.1.tr.WP_027431803.1.WP_027431803.1_NCBI.MULTISPECIES..NADPH.dependen
tr.WP_005427721.1	tr.WP_005427721.1.WP_005427721.1_NCBI.MULTISPECIES..BMC.domain.containing.protein..Clostridiales..tr.A5ZM72.A5ZM72_9FIRM.BMC.domain.protein.OS.Blautia.obeum.ATCC.29174.OX.411459.GN.RUMOBE_00089.PE.4.SV.1.tr.A0A564VDD8.A0A564VDD8_9FIRM.Propanediol.utilizati
tr.A6L1G4	tr.A6L1G4.A6L1G4_BACV8.Putative.outer.membrane.protein..probably.involved.in.nutrient.binding.OS.Bacteroides.vulgatus..strain.ATCC.8482...DSM.1447...JCM.5826...NBRC.14291...NCTC.11154..OX.435590.GN.BVU_1853.PE.3.SV.1.tr.A0A6M4L8X6.A0A6M4L8X6_9BACT.RagB.Sus
tr.E5XXC7	tr.E5XXC7.E5XXC7_BIFLN.Alanine..tRNA.ligase.OS.Bifidobacterium.longum.OX.216816.GN.alaS.PE.3.SV.1.tr.A0A0A1GR05.A0A0A1GR05_BIFLN.Alanine..tRNA.ligase.OS.Bifidobacterium.longum.OX.216816.GN.alaS.PE.3.SV.1
tr.A0A0M6WFJ8	tr.A0A0M6WFJ8.A0A0M6WFJ8_9FIRM.3.hydroxybutyryl.CoA.dehydrogenase.OS.Roseburia.faecis.OX.301302.GN.paaH.PE.4.SV.1
tr.WP_142690962.1	tr.WP_142690962.1.WP_142690962.1_NCBI.MULTISPECIES..NADP.specific.glutamate.dehydrogenase..Clostridium..tr.WP_008677981.1.WP_008677981.1_NCBI.MULTISPECIES..NADP.specific.glutamate.dehydrogenase..Clostridium..tr.A0A174CC76.A0A174CC76_9CLOT.Glutamate.dehydro
tr.D4MYK3	tr.D4MYK3.D4MYK3_ANAHA.Methionine.synthase.II..Cobalamin.independent..OS.Anaerostipes.hadrus.OX.649756.GN.CL2_06510.PE.4.SV.1.tr.A0A5B3GKY4.A0A5B3GKY4_ANAHA.5.methyltetrahydropteroyltriglutamate..homocysteine.S.methyltransferase.OS.Anaerostipes.hadrus.OX.6
tr.A6L7K3	tr.A6L7K3.A6L7K3_BACV8.Major.outer.membrane.protein.OmpA.OS.Bacteroides.vulgatus..strain.ATCC.8482...DSM.1447...JCM.5826...NBRC.14291...NCTC.11154..OX.435590.GN.BVU_4065.PE.4.SV.1
tr.WP_025577186.1	tr.WP_025577186.1.WP_025577186.1_NCBI.MULTISPECIES..polyribonucleotide.nucleotidyltransferase..Clostridiales.
tr.WP_148461524.1	tr.WP_148461524.1.WP_148461524.1_NCBI.MULTISPECIES..ABC.transporter.substrate.binding.protein..Clostridiales..tr.WP_118577606.1.WP_118577606.1_NCBI.MULTISPECIES..ABC.transporter.substrate.binding.protein..Clostridiales..tr.WP_117851081.1.WP_117851081.1_NCB

Table S23. (continued)

Table S23B. (continued)

Abbreviation	Fasta Maxquant
tr.WP_187565966.1	tr.WP_187565966.1.WP_187565966.1_NCBI.knotted.carbamoyltransferase.YgeW..Blautia.faecis..tr.WP_118583376.1.WP_118583376.1_NCBI.MULTISPECIES..knotted.carbamoyltransferase.YgeW..Clostridiales.
tr.A0A2N0SUC3	tr.A0A2N0SUC3.A0A2N0SUC3_BIFLN.Sugar.binding.protein.OS.Bifidobacterium.longum.OX.216816.GN.DPC6316_1257.PE.4.SV.1.tr.A0A0M4LVR1.A0A0M4LVR1_BIFLI.ABC.superfamily.ATP.binding.cassette.transporter..solute.binding.protein.OS.Bifidobacterium.longum.subsp..infa
tr.A0A0H2P4J2	tr.A0A0H2P4J2.A0A0H2P4J2_BIFBI.Elongation.factor.Ts.OS.Bifidobacterium.bifidum.OX.1681.GN.tsf.PE.3.SV.1
tr.A0A151C3V8	tr.A0A151C3V8.A0A151C3V8_BIFLN.ABC.transporter.substrate.binding.protein.OS.Bifidobacterium.longum.OX.216816.GN.APS65_07205.PE.4.SV.1.tr.D4BS49.D4BS49_BIFBR.ABC.transporter..substrate.binding.protein..family.5.OS.Bifidobacterium.breve.DSM.20213...JCM.1192
tr.A0A6N7WKN0	tr.A0A6N7WKN0.A0A6N7WKN0_9FIRM.Electron.transfer.flavoprotein.subunit.alpha.FixB.family.protein.OS.Eisenbergiella.tayi.OX.1432052.GN.FYJ45_23290.PE.4.SV.1.tr.A0A3E3I2C7.A0A3E3I2C7_9FIRM.Electron.transfer.flavoprotein.subunit.alpha.FixB.family.protein.OS.Ei
tr.D4MZD8	tr.D4MZD8.D4MZD8_ANAHA.LL.diaminopimelate.aminotransferase.OS.Anaerostipes.hadrus.OX.649756.GN.dapL.PE.3.SV.1.tr.WP_187565694.1.WP_187565694.1_NCBI.LL.diaminopimelate.aminotransferase..Blautia.faecis..tr.WP_173770055.1.WP_173770055.1_NCBI.MULTISPECIES..LL.
tr.D4N105	tr.D4N105.D4N105_ANAHA.Inositol.2.dehydrogenase.OS.Anaerostipes.hadrus.OX.649756.GN.iolG.PE.4.SV.1
tr.WP_070088499.1	tr.WP_070088499.1.WP_070088499.1_NCBI.elongation.factor.G..Merdimonas.faecis..tr.A0A2Y9BKF8.A0A2Y9BKF8_9FIRM.Elongation.factor.G.OS.Faecalicatena.orotica.OX.1544.GN.fusA.PE.3.SV.1
tr.WP_187565034.1	tr.WP_187565034.1.WP_187565034.1_NCBI.substrate.binding.domain.containing.protein..Blautia.faecis..tr.WP_173717939.1.WP_173717939.1_NCBI.MULTISPECIES..substrate.binding.domain.containing.protein..Clostridiales..tr.WP_148461195.1.WP_148461195.1_NCBI.MULTISP
tr.A0A0A1GN56	tr.A0A0A1GN56.A0A0A1GN56_BIFLN.ANTAR.domain.containing.protein.OS.Bifidobacterium.longum.OX.216816.GN.APC1462_0649.PE.4.SV.1.tr.A0A2N0SW01.A0A2N0SW01_BIFLN.Response.regulator.with.RNA.binding.domain.OS.Bifidobacterium.longum.OX.216816.GN.DPC6316_0707.PE.4
tr.F0FPW5	tr.F0FPW5.F0FPW5_STRSA.Glyceraldehyde.3.phosphate.dehydrogenase.OS.Streptococcus.sanguinis.SK678.OX.888819.GN.gap.PE.3.SV.1.tr.A0A2I1Z9P0.A0A2I1Z9P0_STRSL.Glyceraldehyde.3.phosphate.dehydrogenase.OS.Streptococcus.salivarius.OX.1304.GN.gap.PE.3.SV.1.tr.A0A0
tr.D4BPL1	tr.D4BPL1.D4BPL1_BIFBR.WYL.domain.containing.protein.OS.Bifidobacterium.breve.DSM.20213...JCM.1192.OX.518634.GN.BIFBRE_04026.PE.4.SV.1.tr.A0A2N0SV86.A0A2N0SV86_BIFLN.WYL.domain.containing.protein.OS.Bifidobacterium.longum.OX.216816.GN.DPC6316_0991.PE.4.SV.
tr.A0A087DM86	tr.A0A087DM86.A0A087DM86_BIFAD.Proline..tRNA.ligase.OS.Bifidobacterium.stercoris.JCM.15918.OX.1437612.GN.proS.PE.3.SV.1.tr.A0A087D7J0.A0A087D7J0_9BIFI.Proline..tRNA.ligase.OS.Bifidobacterium.scardovii.OX.158787.GN.proS.PE.3.SV.1.tr.E5XXY8.E5XXY8_BIFLN.Prol
tr.WP_173755788.1	tr.WP_173755788.1.WP_173755788.1_NCBI.MULTISPECIES..carboxyl.transferase..Clostridiales..tr.A0A564VMA6.A0A564VMA6_9FIRM.Methylmalonyl.CoA.carboxyltransferase.12S.subunit.OS.Blautia.luti.OX.89014.GN.RSSSTS7063_02609.PE.4.SV.1.tr.WP_173737800.1.WP_173737800.
tr.A0A1V8PQF9	tr.A0A1V8PQF9.A0A1V8PQF9_9BIFI.Transketolase.OS.Bifidobacterium.catenulatum.OX.1686.GN.B5782_0915.PE.3.SV.1

Abbreviation	Proteins Ids
tr.WP_008705693.1.	WP_008705693.1;WP_173726912.1;WP_117854094.1;A0A4R4FB20;WP_070087401.1;D4MZD9;A0A6N7WM25;A5ZQR8;A0A413FLR6;A0A3E3I955;A0A2Y9CAV0;A0A1I0AVI8;A0A173XPM4;A0A6P1Z5F2;A0A1E3AC12;A0A413QC06;WP_173752049.1
tr.A0A0A1GRX3.	A0A0A1GRX3;A0A1S2VY79;A0A0M3T5H1;A0A126ST81;A0A315S068;KFI89168.1;WP_051912151.1;AUR34142.1
tr.A0A5B3GKY1.	A0A5B3GKY1;WP_144365668.1
tr.WP_015525727.1	WP_015525727.1;A5ZUI6;A0A564W402;WP_008707497.1;A0A1K1PRP2;A0A1H6I7Y0;A0A6P1ZBB0;A0A4Q1RH04;A0A1V4ID03
tr.A0A173R7M3.	A0A173R7M3;WP_070087926.1;Q0TMQ8;A0A2Y9BM50;A0A174LMQ2
tr.A0A4V1NS38.	A0A4V1NS38;A0A564W4Q7;A0A6P1Z5F6;WP_008707261.1;WP_118062095.1;WP_158421032.1;WP_117852531.1;WP_015553837.1;WP_009267277.1;C0FTI7;A0A413F9M6;A0A3E2WBN9;A0A2V3YPY2;A0A1I0HXR3;A0A174GGM7;WP_097002538.1;A0A0M6WYV2;A0A174ADQ2;A0A2S6HH86;WP_022067329.1;A5ZW85;A0A2Y9BG59;A0A1G9XH09;A0A174LQZ1;WP_070087931.1;WP_173771785.1;WP_027430844.1

Table S23. (continued)

Table S23B. (continued)

Abbreviation	Proteins Ids
tr.WP_022380971.1.	WP_022380971.1;WP_173719039.1;WP_173735377.1;WP_118578644.1;WP_117853454.1;WP_022067624.1;A5ZQ54;A0A564W8Q0;WP_173738689.1;WP_015525811.1;A0A4R4FE92;C7GAH5;A0A6P1Z6C4;A0A4Q1RIX2;G2T1E6;A0A2Y9BN18;C0FVN1;A0A3E2WUK9;A0A1E3AD06;A0A6N7WD69;WP_027430390.1;A0A413WJH6;A0A3E3I9S6;A0A174GH96;A0A1G9U6J6;A0A0M6WS73;A0A173TCU9;A0A174F7D3;A0A1I0H993;A0A2S6HUR7;A0A2V3Y1G6;A0A413FAV0;WP_097005542.1;A0A1V4J006;WP_158422029.1;A0A173ZXD9;A0A0N8VX13;WP_009267853.1;A0A174BAJ2;WP_022151344.1;WP_070090232.1;A0A1S8N435;A0A386PFA1;A0A0H2YNR2;A0A173Z9Q1;A0A1E3A2X2;A0A174E692;A0A174K819;WP_097034472.1;WP_008681675.1;WP_099346342.1;A0A3E2VQ31;A0A1T4X1Z3
tr.WP_025577282.1.	WP_025577282.1;WP_173766836.1;A0A4Q1RGS8;A0A6P1Z6X8;A0A5M8BN79;A0A413JMQ1
tr.A0A087DLM8.	A0A087DLM8;A0A1V8Q5W3;A0A315RZ06
tr.A0A087DSI3.	A0A087DSI3;A0A6L9SNF1;A0A1V8Q4C1;A0A1V8PQK8
tr.WP_020993932.1.	WP_020993932.1;E6KAQ5;A0A6G1VSS5;D1PAP8
tr.A0A1L8SSH0.	A0A1L8SSH0
tr.A0A0H2PPI2.	A0A0H2PPI2;A0A0M5KVQ9;WP_193531426.1;WP_033508992.1;KFI85655.1;A0A087D5E0
tr.WP_008704844.1.	WP_008704844.1;WP_173773688.1;A0A174F5E9
tr.A0A4R4FHH5.	A0A4R4FHH5;A0A2Y9BND9;WP_195609348.1;WP_054705823.1;WP_070088226.1
tr.WP_070089092.1.	WP_070089092.1
tr.A0A6M4KU41.	A0A6M4KU41
tr.A0A174AKF1.	A0A174AKF1;WP_075844067.1;WP_040219392.1;G1WFC2
tr.D4BNB9.	D4BNB9
tr.D4MZ58.	D4MZ58
tr.WP_025578671.1.	WP_025578671.1
tr.A0A6L4V7V3.	A0A6L4V7V3;A0A1V8PT76;A0A6L9SIC9;A0A1V8Q7R8;A0A126SVY1;D4BP20
tr.A0A6P1YYM0.	A0A6P1YYM0
tr.WP_081703234.1.	WP_081703234.1
tr.WP_119239543.1.	WP_119239543.1;A0A564W1T1;WP_173773732.1;A5ZQR5;WP_006941416.1;G0VPW5;EFQ04625.1; chaperonin GroEL, partial [Blautia schinkii]; chaperonin GroEL - Blautia obeum; chaperonin GroEL [Megasphaera micronuciformis]; chaperonin GroEL - Megasphaera elsdenii; chaperonin GroEL [Megasphaera micronuciformis]
tr.A0A2Y9BEA5.	A0A2Y9BEA5
tr.WP_147598065.1.	WP_147598065.1
tr.WP_022380910.1.	WP_022380910.1;A0A173S6X6;A0A6P1Z512;C7G7Z6;A0A0M6WUT8;G2SX55;WP_173728909.1;C0FNR4;WP_119240432.1;A0A4R4FC99;A0A4Q1RGE9;A0A564W7S1;A0A174KYC5;A0A1E3A3J5;WP_009266377.1;WP_097005888.1;WP_144364715.1;A0A2S6HMQ4;E6UIZ7;A0A1G9ZJZ1;WP_027431862.1;A0A174E0K1;A0A2V3XXX8;A0A413WEV5;A0A3E2WNA8;A0A1G5FPR2;A0A3E2VFR5;A0A6I7GEB4;A0A6I2RCJ5;A0A2X2U9Z0;A0A174JFZ8;A0A174H2X0;A0A3E4TQE3
tr.A0A0M6WQN5.	A0A0M6WQN5
tr.WP_022380542.1.	WP_022380542.1;WP_118583680.1;WP_147600151.1;A0A564VFU8;A5ZPS6;WP_119239069.1;WP_158420086.1;WP_021926810.1;WP_173718557.1;A0A174IDD4;WP_070087739.1;WP_173736193.1;A0A4Q1REW2;A0A5B3GIA3;D4MTT0;WP_173771030.1;WP_173773934.1
tr.A0A0M6WNV3.	A0A0M6WNV3
tr.WP_022067013.1.	WP_022067013.1;A0A564VZ92;WP_173736772.1;WP_025577868.1
tr.A0A0H2PPC5.	A0A0H2PPC5;A0A1V8Q3J5;A0A087DKB8;A0A4S5BK52;A0A1V8RHM5;A0A0M4LTC3;A0A0A1GQM7;A0A126SWF8;A0A087DHU5;A0A1V8PQ07;WP_033509287.1;WP_033506196.1;KFI87696.1;A0A315RVD4
tr.D4BS21.	D4BS21;A0A0M4N3H8;A0A0M4LVT6
tr.WP_025576924.1.	WP_025576924.1
tr.WP_173755785.1.	WP_173755785.1;WP_173737803.1;A0A564VNR8;A0A6P1YW95;WP_009266107.1
tr.WP_070087934.1.	WP_070087934.1;WP_009267281.1;A0A2Y9BH55;A0A174GAG4;WP_097002542.1;A0A2S6HH99;A0A4V6P6Q9
tr.A0A3E3AD12.	A0A3E3AD12;A0A3E3EBV7
tr.E5XX70.	E5XX70;A0A0M5KUT4;A0A0M4N4L2;A0A4S5B8F3
tr.A0A133LTJ9.	A0A133LTJ9;A0A0M3T675;E5XVW9;D4BRH4;A0A4S5BCB6;WP_033506522.1
tr.A0A173Z2F2.	A0A173Z2F2;WP_173753429.1;A0A3C1IN92;A0A0W7TSE2;ERJ96518.1;WP_021682584.1;E6UJ30;WP_044505176.1;A0A2U0U309;A0A5B5VNZ8;D4LCK8;G5H543;A0A137SYR6;I3YM14;G6AY02;E6K3W8;D1PFZ9;A0A5B3GPW3;WP_026089666.1;A0A0B0BVA2;A0A1H4C5U0;A0A4Y1XT24;C7GG87;A0A4Y1WS45;A0A1Q6F5P2;A0A1Y3QV15
tr.A0A126SU96.	A0A126SU96;A0A1V8Q7P6
tr.A0A1V8Q6G3.	A0A1V8Q6G3;A0A1V8PSH8;A0A087DLM1
tr.WP_025579304.1	WP_025579304.1;A0A173SJ33;A0A0M6WCQ4;A5ZY79;A0A564VYC3;A0A3E4U4D3;WP_173738803.1
tr.A5ZUS4.	A5ZUS4
tr.A0A1G5BPU3.	A0A1G5BPU3

Table S23. (continued)

Table S23B. (continued)

Abbreviation	Proteins Ids
tr.WP_025578097.1.	WP_025578097.1;A0A0M6WJL5;A5ZTK3;A0A564W042;WP_173756557.1;WP_173736794.1;WP_187565031.1;WP_173773159.1;WP_173717941.1;WP_173767271.1
tr.A0A0M4LTL3.	A0A0M4LTL3;A0A1D7MR69;E5Y117;A0A4S5BLR2;D4BLN5;A0A087DDR3;KFI85589.1;A0A126SVW0;A0A0H2Q6L9;WP_033508912.1;A0A0M4LK22;A0A087DL34;A0A1V8PSZ3;A0A1V8Q855;A0A6L4V736;A0A6L9SKI1;A0A6L8ZVW8
tr.A0A174US18.	A0A174US18
tr.A6L1G5.	A6L1G5;A0A7I0QFN2;A0A4R4HE07
tr.A0A0M6WWS6.	A0A0M6WWS6;WP_144363565.1;G2T184
tr.WP_097005984.1.	WP_097005984.1;A0A5M8BY86;A0A174MDW4;A0A2S6HVW3
tr.A0A3E3ADB3.	A0A3E3ADB3
tr.WP_009268670.1.	WP_009268670.1;WP_117551661.1;C0FQI1;G2T3G7;WP_070089461.1;A0A2Y9BGS1;A0A4R4F9Z3;A0A2V3Y4V4;WP_021904111.1;C7GBS5;A0A1G5AXA6;WP_044960678.1;EFQ04148.1;A0A413WDP0;A0A1V4IZZ8;WP_008678397.1;A0A413FLW2;A0A3E2WKN0;A0A0M6WP42;G0VRY5;WP_173757334.1;A0A1Y4FNF4;A0A564VWV8;A0A174TQH9;WP_097006003.1;Q0TPQ3;A0A1Q6R444;A0A4Q1RIJ0;E6UI98;A0A3G9H664;R6I895;A0A2V2F9M2;A0A386PF17
tr.F3PHE4.	F3PHE4;B0NQ83;A0A3E5GG32;A0A380YPU5;A0A174XA23;A0A139KHW3;A0A0P0GN40;A0A0P0EP49;C6IRM3;B5D0E5;K9DRM2;A0A0K6BWV0;A0A081UKI1
tr.A0A4S5B9L3.	A0A4S5B9L3;A0A087DNL3
tr.A7AIR2.	A7AIR2;A0A415PLT9
tr.WP_008703889.1	WP_008703889.1
tr.WP_195360800.1.	WP_195360800.1
tr.D1PG79.	D1PG79;B3JG78;A0A413T419;E6K4X0;B5CU80;A0A137SYZ7
tr.A0A126SV93.	A0A126SV93
tr.A0A2Y9BGR4.	A0A2Y9BGR4;WP_158420463.1;A0A4R4FHT1;A0A174JK04;C7GEV1
tr.A0A0A1GQP2.	A0A0A1GQP2;A0A4S5BDT8;A0A0M4LH57
tr.WP_028254790.1.	WP_028254790.1
tr.C7GD05.	C7GD05;C0FTI0;WP_158421025.1;A0A4Q1RJL2;WP_070087937.1;A0A4R4FGE8;WP_009267284.1;G2T5N3;A0A2Y9BKK5
tr.WP_156902361.1.	WP_156902361.1;WP_117733070.1;ERJ93286.1
tr.WP_025577065.1.	WP_025577065.1;WP_021926204.1;WP_173727389.1;WP_118578437.1;WP_119240559.1;A5ZYD6;A0A564W7F1
tr.D4MV01.	D4MV01
tr.D4MZ60.	D4MZ60;G2T2F1;A0A3E3I9J7;A0A174XWC3
tr.A0A174D9I7.	A0A174D9I7
tr.A6KXV7.	A6KXV7;A0A7I0RSV7;A0A1Y3ZEA3;A6KXW2;A0A076IXI8;S0GLH8;S0GU45
tr.A0A399IH89.	A0A399IH89;A0A1V4IW50;A0A1V4IEV4;A0A6L9EQW6;A0A1V4IED1;A0A174GEM0;A0A512TS13;A0A427SFZ8;A0A1S8NQ54;A0A413F9E9;A0A1I0JUZ4;A0A1I0EZX4;A0A1S8N304
tr.A0A6P1YTK1.	A0A6P1YTK1;A0A4Q1RH58
tr.D4MUZ9.	D4MUZ9;A0A5B3G355;A0A2Y9BB39;A0A386PDZ9;A0A1V4IUM1;A0A1S8N4Z9;A0A413WBH9;A0A3E2WAC8
tr.D4MXD7.	D4MXD7
tr.A0A087DMJ5.	A0A087DMJ5
tr.D4N113.	D4N113;A0A174NB50;WP_173755379.1;WP_173737600.1;A0A564W3Q9
tr.D4BQK2.	D4BQK2
tr.A0A1V8Q531.	A0A1V8Q531;A0A087DLV3;WP_033505254.1;KFI88796.1;A0A087DJZ7;A0A1V8PP53
tr.WP_025580805.1.	WP_025580805.1;A5ZW30
tr.D4MUG4.	D4MUG4;WP_027430867.1;A0A1G9XIV8;WP_157050167.1;WP_195609617.1
tr.A0A133PIY4.	A0A133PIY4
tr.WP_097006856.1.	WP_097006856.1;A0A174KBQ7;WP_027431919.1;A0A1G9SWX2;A0A3E2W9M9;A0A174C2M6;A0A2S6HCD5;A0A2V3Y0P0;A0A3E2V2Q3;F8DHJ6;A0A413FBK7;WP_200773217.1;F0FVM9;A0A174NRW8;A0A3E3AEK2;A0A3E3EBT2;A0A0M4KA88;A0A0A0DFA8;A0A174DVW2;A0A6N7WM26;A0A4Z1E167;A0A2I1ZB30;A0A1Y4SXD5;A0A6N7WLS9;A0A0A0DEN1;A0A0F5MLB7;WP_173737645.1;A0A2T3F4Y4;E8JR35;A0A174AFU4;A0A5K1IB91;A0A1V4II10;A0A399IK53;WP_173755586.1;A0A1I0FRD9;A0A1I0G5R2;A0A4P8KF53;A0A174JQ13;A5ZM95;A0A1V8ZQC2;A0A437UIP0;A0A1L8X534;A0A2N8PZB5;A0A1J6YEZ6;A0A5K1ID49;A0A2N8PXU6;A0A4P8KJE9;A0A1I0DXE3;A0A413FGM0;A0A2T3FGY7;WP_041139184.1;A0A413JQE5;A0A5M8BR21;A0A1S8MQ85;A0A1L8SWS9;A0A120LLE3;A0A4S3PRG4;A0A174AZS6;A0A1L8W894;S1NMZ8;WP_008676385.1;WP_097034185.1;A0A1L8SX97;A0A1V4IH34;A0A1V8Z779;A0A1L8SWM8;A0A1V8XAB6;A0A386PJ16;A0A1L8SFD2;A0A174U883
tr.D4MYG8.	D4MYG8
tr.C7G6S2	C7G6S2
	A0A2V2FBJ8;A0A1T4X822
tr.WP_173767105.1.	WP_173767105.1;WP_148461413.1

Table S23. (continued)

Table S23B. (continued)

Abbreviation	Proteins Ids
tr.WP_173773355.1.	WP_173773355.1;WP_173718261.1;WP_148462774.1;WP_130790115.1;D4LE66;A0A564W263;A0A413VNK4;A0A3E2WKL7;A0A3E2VGF2
tr.WP_020993443.1.	WP_020993443.1;WP_158420085.1;WP_117851485.1
tr.A0A6P1Z4H2.	A0A6P1Z4H2;A0A174GFP3;A0A0M6X1M0
tr.A0A6P1Z6F1.	A0A6P1Z6F1
tr.A0A4V2X185.	A0A4V2X185;A6L1G3;WP_128812197.1;WP_008657017.1
tr.D4MYK4.	D4MYK4
tr.WP_147600180.1.	WP_147600180.1
tr.A0A2Y9B7L8.	A0A2Y9B7L8;D4MZ07;A5ZPB7;A0A4Q1RGS2;WP_070089089.1;WP_009268311.1
tr.A0A6P1Z1I4.	A0A6P1Z1I4
tr.WP_101723696.1.	WP_101723696.1;A0A369N8X2
tr.D4MZK6	D4MZK6;A0A6N7W712;A0A3E3I2K6;A0A1E3AJZ8
tr.A0A1V8PLT8.	A0A1V8PLT8;A0A6L4V360;A0A1V8Q5E9;WP_051915779.1;WP_051912149.1;KFI89167.1;A0A4V1WJX7;A0A315S4P8;ABA19190.1
tr.WP_027431932.1.	WP_027431932.1;A0A1G9SX47
tr.D4MUH3	D4MUH3;A0A5B3GDA7
tr.A6L1X3	A6L1X3;A0A6M4L9D4;A0A076J6D0
tr.D4MW58.	D4MW58;A0A5B3GIV9
tr.A0A0M3T6V1.	A0A0M3T6V1;A0A4S5BHN7;A0A1V8RGX0;A0A0A1GME2
tr.A0A0M4HKR2.	A0A0M4HKR2;V5QQV9;F8DIT1;A0A4Z1DXX7;A0A0A0DIX0;E8JP94;WP_200772676.1;F0FSN0;A0A2I1ZBV7;A0A0F2CNT5;A0A4S3PKC9;A0A1V8XBY6
sp.P69780.	P69780;C3T887;B7LQ99;A0A6I7EI93;A0A564UQ82;A0A2U2NVJ5;A0A285B574;A0A1W2MPD9;A0A1C1F567;A0A0V9HIR3;A0A0I1IY43;A0A0G3S7W6;A0A094ZTE8;A0A094ZLD6
tr.A0A679HBI1.	A0A679HBI1;D6D5K9;A0A0K6BVG6;A0A139KY96;A0A174VD14;A0A1C7H1V6;A0A1G1UAB5;A0A3A9B9H8;A0A3E5GAJ9;A6L643;A0A6L3FF83;A0A076J997;A0A081UDU1;F3PG16;A0A415RUW3;B0NNK2;A0A380YMF8;A0A174N679
tr.WP_033508353.1.	WP_033508353.1;WP_003827292.1;KFI88792.1;D4BQQ9;A0A315RZT5;A0A1V8Q5C3;A0A1V8PP16;A0A126ST87;A0A0M4LFS8;A0A0E2ZVB9;A0A087DLU9;A0A087DJZ3;A0A075ND16
tr.A6L1G6.	A6L1G6;A0A076IY48
tr.A0A0M4LFR2.	A0A0M4LFR2;A0A0A1GPP2;D4BQT5;A0A315S031
tr.A0A0H2PWI8.	A0A0H2PWI8;A0A0M4LZ74
tr.D4MW30.	D4MW30;A0A1Q2C8D3
tr.D4BLH5.	D4BLH5;A0A1V8PLA3
tr.A0A2U0C039.	A0A2U0C039
tr.WP_195609585.1.	WP_195609585.1;A0A2Y9CAG1
tr.A0A329TW61.	A0A329TW61
tr.A0A415MLA0.	A0A415MLA0
tr.WP_119239650.1.	WP_119239650.1;A0A564W6U6
tr.A0A6P1YWQ6.	A0A6P1YWQ6;A0A2Y9BK62;A0A174L573;WP_097005784.1
tr.A6KWT3.	A6KWT3
tr.D4N0D1.	D4N0D1;A0A412UNN9
tr.WP_148461930.1.	WP_148461930.1
tr.A0A1V8Q6B2.	A0A1V8Q6B2;A0A1V8PS80;A0A087DLN9
tr.D4LD40.	D4LD40;WP_027431803.1;A0A1G9V8Q2;A0A1B8RLK3;WP_117732098.1;WP_040646431.1;A0A5M8BQS2;A0A413JQ55;ERJ92507.1
tr.WP_005427721.1.	WP_005427721.1;A5ZM72;A0A564VDD8;WP_173765997.1;A0A3E3I089;A0A1E3UID9;A0A1E3AGH5
tr.A6L1G4.	A6L1G4;A0A6M4L8X6;A0A076J481;WP_025077303.1
tr.E5XXC7.	E5XXC7;A0A0A1GR05
tr.A0A0M6WFJ8.	A0A0M6WFJ8
tr.WP_142690962.1	WP_142690962.1;WP_008677981.1;A0A174CC76;WP_195624476.1
tr.D4MYK3	D4MYK3;A0A5B3GKY4
tr.A6L7K3.	A6L7K3
tr.WP_025577186.1.	WP_025577186.1;A0A6P1Z2M2;WP_173755338.1;WP_173738696.1;A0A564W959;WP_158422041.1;WP_097002901.1;C7GDJ9;WP_144364355.1;WP_173773798.1;A5ZWK0;A0A2S6HXC2;D4MYT6;WP_173752248.1;WP_118578672.1;A0A413F781;A0A413VKB7;A0A4Q1RIN1;A0A174JVU4;A0A5M8BTV2;A0A0M6WNL5;WP_173773849.1;G2T329;WP_148462345.1;A0A6N7WKA8;C0FP37;A0A174J7F7;WP_027431781.1;A0A1G9V7D5;A0A2Y9B760;A0A1E3AG33;A0A3E2W7H1;A0A3E3ILL9;A0A2V3YF43
tr.WP_148461524.1.	WP_148461524.1;WP_118577606.1;WP_117851081.1
tr.WP_187565966.1.	WP_187565966.1;WP_118583376.1

Table S23. (continued)

Table S23B. (continued)

Abbreviation	Proteins Ids
tr.A0A2N0SUC3.	A0A2N0SUC3;A0A0M4LVR1;A0A0A1GPF7;A0A4V3YVQ3
tr.A0A0H2P4J2.	A0A0H2P4J2
tr.A0A151C3V8.	A0A151C3V8;D4BS49
tr.A0A6N7WKN0.	A0A6N7WKN0;A0A3E3I2C7;A0A1E3APB7;WP_015553779.1;A0A413JP99
tr.D4MZD8.	D4MZD8;WP_187565694.1;WP_173770055.1;WP_173768230.1;WP_148462916.1;WP_070087872.1;G2T2F5;C0FNL3
tr.D4N105.	D4N105
tr.WP_070088499.1.	WP_070088499.1;A0A2Y9BKF8
tr.WP_187565034.1.	WP_187565034.1;WP_173717939.1;WP_148461195.1;WP_117850689.1;WP_025577577.1
tr.A0A0A1GN56.	A0A0A1GN56;A0A2N0SW01;A0A4S5BKX2;A0A0A8N580;WP_043168699.1;WP_033509292.1;KFI87692.1;A0A087DHT9
tr.F0FPW5.	F0FPW5;A0A2I1Z9P0;A0A0P6UMZ0;A0A074JDF4
tr.D4BPL1.	D4BPL1;A0A2N0SV86;A0A0A8NEK8
tr.A0A087DM86.	A0A087DM86;A0A087D7J0;E5XXY8;WP_193531471.1;WP_033508532.1;D4BR60;A0A0M4LZ14;KFI88958.1;A0A4S5BF81;A0A2N0SRR0;A0A126SXC2;A0A6L4V6X2;A0A1V8Q6X9;A0A1V8PPL6;A0A0M4MGZ4;A0A0H2PQR7
tr.WP_173755788.1.	WP_173755788.1;A0A564VMA6;WP_173737800.1;A5ZQB6
tr.A0A1V8PQF9.	A0A1V8PQF9

Abbreviation	Protein names
tr.WP_008705693.1.	IMP cyclohydrolase [Clostridiales]; IMP cyclohydrolase [*Blautia glucerasea*]; IMP cyclohydrolase [Clostridiales]; IMP cyclohydrolase - *Extibacter muris*; IMP cyclohydrolase [*Merdimonas faecis*]; IMP cyclohydrolase-like protein - *Anaerostipes hadrus*; IMP cyclohydrolase - *Eisenbergiella porci*; IMP cyclohydrolase-like protein - *Blautia obeum*; IMP cyclohydrolase - *Enterocloster asparagiformis*; IMP cyclohydrolase - *Eisenbergiella massiliensis*; IMP cyclohydrolase-like protein - *Faecalicatena orotica*; IMP cyclohydrolase - *Enterocloster lavalensis*; IMP cyclohydrolase - *Anaerostipes hadrus*; IMP cyclohydrolase - *Blautia producta*; IMP cyclohydrolase - *Eisenbergiella tayi*; IMP cyclohydrolase - *Ruminococcus bromii*; IMP cyclohydrolase [*Blautia*]
tr.A0A0A1GRX3.	DNA-directed RNA polymerase subunit beta - *Bifidobacterium longum*; DNA-directed RNA polymerase subunit beta - *Bifidobacterium longum subsp. Infantis*; DNA-directed RNA polymerase subunit beta - *Bifidobacterium longum subsp. Infantis*; DNA-directed RNA polymerase subunit beta - *Bifidobacterium angulatum*; DNA-directed RNA polymerase subunit beta - *Bifidobacterium animalis subsp. lactis* (*Bifidobacterium lactis*); DNA-directed RNA polymerase subunit beta - *Bifidobacterium pullorum subsp. saeculare*; DNA-directed RNA polymerase subunit beta - *Bifidobacterium pullorum*; DNA-directed RNA polymerase subunit beta, partial - *Bifidobacterium pullorum subsp. saeculare*
tr.A0A5B3GKY1.	Class II fructose-1,6-bisphosphate aldolase - *Anaerostipes hadrus*; Class II fructose-1,6-bisphosphate aldolase [*Lacrimispora amygdalina*]
tr.WP_015525727.1.	GGGtGRT protein [Clostridiales]; Uncharacterized protein - *Blautia obeum*; GGGtGRT protein - *Blautia Luti*; hypothetical protein [Clostridiales]; GGGtGRT protein - *Ruminococcus flavefaciens*; GGGtGRT protein - *Ruminococcus flavefaciens*; GGGtGRT protein - *Blautia producta*; GGGtGRT protein - *Blautia faecicola*; GGGtGRT protein - *Clostridium chromiireducens*
tr.A0A173R7M3.	50S ribosomal protein L5 - *Anaerostipes hadrus*; 50S ribosomal protein L5 [Eubacteriales]; 50S ribosomal protein L5 - *Clostridium perfringens*; 50S ribosomal protein L5 - *Faecalicatena orotica*; 50S ribosomal protein L5 - *Lachnospira pectinoschiza*
tr.A0A4V1NS38.	50S ribosomal protein L16 - *Blautia faecicola*; 50S ribosomal protein L16 - *Blautia luti*; 50S ribosomal protein L16 - *Blautia producta*; 50S ribosomal protein L16 [*Blautia*]; 50S ribosomal protein L16 [Eubacteriales]; 50S ribosomal protein L16 [*Blautia*]; 50S ribosomal protein L16 [Eubacteriales]; 50S ribosomal protein L16 [Eubacteriales]; 50S ribosomal protein L16 [*Mediterraneibacter glycyrrhizinilyticus*]; 50S ribosomal protein L16 - *Roseburia inulinivorans*; 50S ribosomal protein L16 - *Enterocloster asparagiformis*; 50S ribosomal protein L16 - *Enterocloster aldenensis*; 50S ribosomal protein L16 - *Hungatella effluvii*; 50S ribosomal protein L16 - *Enterocloster lavalensis*; 50S ribosomal protein L16 - *Clostridium symbiosum* (*Bacteroides symbiosus*); 50S ribosomal protein L16 [Eubacteriales]; 50S ribosomal protein L16 - *Roseburia faecis*; 50S ribosomal protein L16 - *Hungatella hathewayi*; 50S ribosomal protein L16 - *Hungatella xylanolytica*; 50S ribosomal protein L16 [*Blautia*]; 50S ribosomal protein L16 - *Blautia obeum*; 50S ribosomal protein L16 - *Faecalicatena orotica*; 50S ribosomal protein L16 - *Lachnospira pectinoschiza*; 50S ribosomal protein L16 - *Lachnospira pectinoschiza*; 50S ribosomal protein L16 [Eubacteriales]; 50S ribosomal protein L16 [Eubacteriales]; 50S ribosomal protein L16 [*Lachnospira*]

Table S23. (continued)

Table S23B. (continued)

Abbreviation	Protein names
tr.WP_022380971.1.	glycine--tRNA ligase [Clostridiales]; glycine--tRNA ligase [Clostridiales]; glycine--tRNA ligase [Eubacteriales]; glycine--tRNA ligase [Eubacteriales]; glycine--tRNA ligase [Eubacteriales]; glycine--tRNA ligase [*Blautia*]; glycine--tRNA ligase - *Blautia obeum*; glycine--tRNA ligase - *Blautia luti*; glycine--tRNA ligase [*Blautia schinkii*]; glycine--tRNA ligase [Eubacteriales]; Glycine--tRNA ligase - *Extibacter muris*; Glycine--tRNA ligase - *Roseburia intestinalis*; glycine--tRNA ligase - *Blautia producta*; glycine--tRNA ligase - *Blautia faecicola*; glycine--tRNA ligase - *Roseburia hominis*; glycine--tRNA ligase - *Faecalicatena orotica*; glycine--tRNA ligase - *Roseburia inulinivorans*; glycine--tRNA ligase - *Enterocloster aldenensis*; glycine--tRNA ligase - *Eisenbergiella tayi*; glycine--tRNA ligase - *Eisenbergiella porci*; glycine--tRNA ligase [*Lachnospira*]; glycine--tRNA ligase - *Enterocloster aldenensis*; glycine--tRNA ligase - *Eisenbergiella massiliensis*; glycine--tRNA ligase - *Lachnospira pectinoschiza*; glycine--tRNA ligase - *Lachnospira pectinoschiza*; glycine--tRNA ligase - *Roseburia faecis*; glycine--tRNA ligase - *Roseburia faecis*; glycine--tRNA ligase - *Enterocloster clostridioformis*; glycine--tRNA ligase - *Enterocloster lavalensis*; glycine--tRNA ligase - *Hungatella xylanolytica*; glycine--tRNA ligase - *Hungatella effluvii*; glycine--tRNA ligase - *Enterocloster asparagiformis*; glycine--tRNA ligase [*Lacrimispora amygdalina*]; glycine--tRNA ligase - *Clostridium chromiireducens*; glycine--tRNA ligase [*Blautia*]; glycine--tRNA ligase - *Clostridium symbiosum* (*Bacteroides symbiosus*); glycine--tRNA ligase - *Clostridium butyricum*; glycine--tRNA ligase [*Mediterraneibacter glycyrrhizinilyticus*]; glycine--tRNA ligase - *Hungatella hathewayi*; glycine--tRNA ligase [*Mediterraneibacter glycyrrhizinilyticus*]; glycine--tRNA ligase [*Merdimonas faecis*]; glycine--tRNA ligase - *Clostridium saccharobutylicum*; glycine--tRNA ligase - *Clostridium septicum*; glycine--tRNA ligase - *Clostridium perfringens*; glycine--tRNA ligase - *Faecalibacterium prausnitzii*; glycine--tRNA ligase - *Eisenbergiella tayi*; glycine--tRNA ligase - *Clostridium disporicum*; glycine--tRNA ligase - *Clostridium disporicum*; glycine--tRNA ligase [*Clostridium*]; glycine--tRNA ligase [*Clostridium*]; glycine--tRNA ligase [*Clostridium*]; glycine--tRNA ligase - *Enterocloster citroniae*; glycine--tRNA ligase - *Gemmiger formicilis*
tr.WP_025577282.1.	phosphoglycerate kinase [Clostridiales]; phosphoglycerate kinase [*Blautia glucerasea*]; phosphoglycerate kinase - *Blautia faecicola*; phosphoglycerate kinase - *Blautia producta*; phosphoglycerate kinase - *Clostridium symbiosum* (*Bacteroides symbiosus*); phosphoglycerate kinase - *Clostridium symbiosum* (*Bacteroides symbiosus*)
tr.A0A087DLM8.	50S ribosomal protein L1 - *Bifidobacterium stercoris*; 50S ribosomal protein L1 - *Bifidobacterium dentium*; 50S ribosomal protein L1 - *Bifidobacterium animalis subsp. lactis* (*Bifidobacterium lactis*)
tr.A0A087DSI3.	Isocitrate dehydrogenase [NADP] - *Bifidobacterium stercoris*; Isocitrate dehydrogenase [NADP] - *Bifidobacterium dentium*; Isocitrate dehydrogenase [NADP] - *Bifidobacterium dentium*; Isocitrate dehydrogenase [NADP] - *Bifidobacterium catenulatum*
tr.WP_020993932.1.	formate C-acetyltransferase [Clostridiales]; formate C-acetyltransferase - *Prevotella buccae*; formate C-acetyltransferase - *Prevotella copri*; formate C-acetyltransferase - *Prevotella copri*
tr.A0A1L8SSH0.	Glutamate dehydrogenase - *Enterococcus devriesei*
tr.A0A0H2PPI2.	Calcium-transporting ATPase - *Bifidobacterium bifidum*; Cation-transporting ATPase PacL - *Bifidobacterium bifidum*; cation-translocating P-type ATPase [*Bifidobacterium pullorum*]; cation-translocating P-type ATPase [*Bifidobacterium pullorum*]; cation-transporting ATPase PacL [*Bifidobacterium pullorum subsp. saeculare*]; Calcium-transporting ATPase - *Bifidobacterium scardovii*
tr.WP_008704844.1.	ketol-acid reductoisomerase [Clostridiales]; ketol-acid reductoisomerase, partial [*Blautia schinkii*]; ketol-acid reductoisomerase (NADP(+)) - *Flavonifractor plautii* (*Fusobacterium plautii*)
tr.A0A4R4FHH5.	Elongation factor G - *Extibacter muris*; Translation elongation factor 2 (EF-2/EF-G) - *Faecalicatena orotica*; elongation factor G [*Mediterraneibacter glycyrrhizinilyticus*]; elongation factor G [*Mediterraneibacter glycyrrhizinilyticus*]; elongation factor G [*Merdimonas faecis*]
tr.WP_070089092.1.	type I glyceraldehyde-3-phosphate dehydrogenase [*Merdimonas faecis*]
tr.A0A6M4KU41.	Uncharacterized protein - *Phocaeicola dorei*
tr.A0A174AKF1.	Glyceraldehyde-3-phosphate dehydrogenase - *Collinsella aerofaciens*; Glyceraldehyde-3-phosphate dehydrogenase [*Collinsella bouchesdurhonensis*]; Glyceraldehyde-3-phosphate dehydrogenase [*Collinsella bouchesdurhonensis*]; Glyceraldehyde-3-phosphate dehydrogenase - *Collinsella tanakaei*
tr.D4BNB9.	Carbamoyl-phosphate synthase large chain - *Bifidobacterium breve*
tr.D4MZ58.	Dihydropyrimidine dehydrogenase - *Anaerostipes hadrus*
tr.WP_025578671.1.	pyruvate:ferredoxin (flavodoxin) oxidoreductase [Clostridiales]
tr.A0A6L4V7V3.	Alpha-1,4 glucan phosphorylase - *Bifidobacterium catenulatum*; Alpha-1,4 glucan phosphorylase - *Bifidobacterium catenulatum*; Alpha-1,4 glucan phosphorylase - *Bifidobacterium dentium*; Alpha-1,4 glucan phosphorylase - *Bifidobacterium dentium*; Alpha-1,4 glucan phosphorylase - *Bifidobacterium angulatum*; Alpha-1,4 glucan phosphorylase - *Bifidobacterium breve*
tr.A0A6P1YYM0.	Class II fructose-1,6-bisphosphate aldolase - *Blautia producta*
tr.WP_081703234.1.	diaminopimelate epimerase [*Blautia wexlerae*]
tr.WP_119239543.1.	chaperonin GroEL [Clostridiales]; 60 kDa chaperonin - *Blautia luti*;
tr.A0A2Y9BEA5.	Propionaldehyde dehydrogenase - *Faecalicatena orotica*
tr.WP_147598065.1.	phosphoenolpyruvate carboxykinase (ATP) [*Blautia caecimuris*]

Table S23. (continued)

Table S23B. (continued)

Abbreviation	Protein names
tr.WP_022380910.1.	adenylosuccinate synthase [Clostridiales]; adenylosuccinate synthetase - *Anaerostipes hadrus*; adenylosuccinate synthetase - *Blautia producta*; adenylosuccinate synthetase - *Roseburia intestinalis*; adenylosuccinate synthetase - *Roseburia faecis*; adenylosuccinate synthetase - *Roseburia hominis*; adenylosuccinate synthase [*Blautia glucerasea*]; adenylosuccinate synthetase - *Roseburia inulinivorans*; adenylosuccinate synthase [Eubacteriales]; adenylosuccinate synthetase - *Extibacter muris*; adenylosuccinate synthetase - *Blautia faecicola*; adenylosuccinate synthetase - *Blautia luti*; adenylosuccinate synthetase - *Lachnospira pectinoschiza*; adenylosuccinate synthetase - *Eisenbergiella tayi*; adenylosuccinate synthase [*Mediterraneibacter glycyrrhizinilyticus*]; adenylosuccinate synthase [*Lacrimispora amygdalina*]; adenylosuccinate synthase [*Lacrimispora amygdalina*]; adenylosuccinate synthetase - *Hungatella xylanolytica*; adenylosuccinate synthetase - *Ruminococcus albus*; adenylosuccinate synthetase - *Lachnospira pectinoschiza*; adenylosuccinate synthase [*Lachnospira*]; adenylosuccinate synthetase - *Hungatella hathewayi*; adenylosuccinate synthetase - *Hungatella effluvii*; adenylosuccinate synthetase - *Enterocloster aldenensis*; adenylosuccinate synthetase - *Ruminococcus bromii*; adenylosuccinate synthetase - *Enterocloster citroniae*; adenylosuccinate synthetase - *Flavonifractor plautii* (*Fusobacterium plautii*); adenylosuccinate synthetase - *Flavonifractor plautii* (*Fusobacterium plautii*); adenylosuccinate synthetase - *Enterocloster clostridioformis*; adenylosuccinate synthetase - *Clostridium symbiosum (Bacteroides symbiosus)*; adenylosuccinate synthetase - *Enterocloster clostridioformis*; adenylosuccinate synthetase - *Hungatella hathewayi*
tr.A0A0M6WQN5.	Uncharacterized protein - *Roseburia faecis*
tr.WP_022380542.1.	single-stranded DNA-binding protein [Clostridiales]; single-stranded DNA-binding protein [Clostridiales]; single-stranded DNA-binding protein [*Blautia*]; single-stranded DNA-binding protein - *Blautia luti*; single-stranded DNA-binding protein - *Blautia obeum*; single-stranded DNA-binding protein [Eubacteriales]; single-stranded DNA-binding protein [*Blautia*]; single-stranded DNA-binding protein [Eubacteriales]; single-stranded DNA-binding protein [Eubacteriales]; single-stranded DNA-binding protein - Clostridium symbiosum (*Bacteroides symbiosus*); single-stranded DNA-binding protein [*Merdimonas faecis*]; single-stranded DNA-binding protein [*Blautia*]; single-stranded DNA-binding protein - *Blautia faecicola*; single-stranded DNA-binding protein - *Anaerostipes hadrus*; single-stranded DNA-binding protein - *Anaerostipes hadrus*; single-stranded DNA-binding protein [Eubacteriales]; single-stranded DNA-binding protein [*Blautia faecis*]
tr.A0A0M6WNV3.	BIG2 domain-containing protein - *Roseburia faecis*
tr.WP_022067013.1.	glucosamine-6-phosphate deaminase [Clostridiales]; Glucosamine-6-phosphate deaminase - *Blautia luti*; glucosamine-6-phosphate deaminase [Eubacteriales]; glucosamine-6-phosphate deaminase [*Blautia*]
tr.A0A0H2PPC5.	30S ribosomal protein S1 - *Bifidobacterium bifidum*; 30S ribosomal protein S1 - *Bifidobacterium dentium*; 30S ribosomal protein S1 - *Bifidobacterium adolescentis*; 30S ribosomal protein S1 - *Bifidobacterium longum subsp. Infantis*; 30S ribosomal protein S1 - *Bifidobacterium longum*; 30S ribosomal protein S1 - *Bifidobacterium longum subsp. infantis*; 30S ribosomal protein S1 - *Bifidobacterium longum*; 30S ribosomal protein S1 - *Bifidobacterium angulatum*; 30S ribosomal protein S1 - *Bifidobacterium scardovii*; 30S ribosomal protein S1 - *Bifidobacterium catenulatum*; 30S ribosomal protein S1 [*Bifidobacterium pullorum*]; 30S ribosomal protein S1 [*Bifidobacterium*]; 30S ribosomal protein S1 [*Bifidobacterium pullorum subsp. saeculare*]; 30S ribosomal protein S1 - *Bifidobacterium animalis subsp. lactis* (*Bifidobacterium lactis*)
tr.D4BS21.	Glyceraldehyde-3-phosphate dehydrogenase, type I - *Bifidobacterium breve*; Glyceraldehyde 3-phosphate dehydrogenase C - *Bifidobacterium longum*; NAD-dependent glyceraldehyde-3-phosphate dehydrogenase - *Bifidobacterium longum subsp. Infantis*
tr.WP_025576924.1.	phosphate acetyltransferase [*Blautia wexlerae*]
tr.WP_173755785.1.	oxaloacetate decarboxylase subunit alpha [*Blautia schinkii*]; oxaloacetate decarboxylase subunit alpha [*Blautia schinkii*]; Methylmalonyl-CoA carboxyltransferase 5S subunit - *Blautia luti*; Oxaloacetate decarboxylase subunit alpha - *Blautia producta*; oxaloacetate decarboxylase subunit alpha [*Mediterraneibacter glycyrrhizinilyticus*]
tr.WP_070087934.1.	50S ribosomal protein L2 [*Merdimonas faecis*]; 50S ribosomal protein L2 [*Mediterraneibacter glycyrrhizinilyticus*]; 50S ribosomal protein L2 - *Faecalicatena orotica*; 50S ribosomal protein L2 - *Clostridium symbiosum* (*Bacteroides symbiosus*); 50S ribosomal protein L2 [Eubacteriales]; 50S ribosomal protein L2 - *Hungatella xylanolytica*; 50S ribosomal protein L2 - *Extibacter muris*
tr.A0A3E3AD12.	Carbohydrate ABC transporter substrate-binding protein - *Erysipelatoclostridium ramosum*; Carbohydrate ABC transporter substrate-binding protein - *Thomasclavelia ramosa*
tr.E5XX70.	UDP-glucose 4-epimerase - *Bifidobacterium longum*; UDP-glucose 4-epimerase - *Bifidobacterium longum subsp. Infantis*; UDP-glucose 4-epimerase - *Bifidobacterium longum*; UDP-glucose 4-epimerase - *Bifidobacterium longum subsp. Infantis*
tr.A0A133LTJ9.	Aspartate--tRNA(Asp/Asn) ligase - *Bifidobacterium longum*; Aspartate--tRNA(Asp/Asn) ligase - *Bifidobacterium longum subsp. Infantis*; Aspartate--tRNA(Asp/Asn) ligase; Aspartate--tRNA(Asp/Asn) ligase - *Bifidobacterium breve*; Aspartate--tRNA(Asp/Asn) ligase - *Bifidobacterium longum subsp. infantis*; aspartate--tRNA ligase [*Bifidobacterium*]

Table S23. (continued)

Table S23B. (continued)

Abbreviation	Protein names
tr.A0A173Z2F2.	Elongation factor G - *Anaerostipes hadrus*; GTP-binding protein, partial [*Blautia caecimuris*]; Elongation factor G - *Phascolarctobacterium succinatutens*; Elongation factor G - *Ruthenibacterium lactatiformans*; translation elongation factor G [*Ruminococcus callidus*]; Elongation factor G [*Ruminococcus*]; Elongation factor G - *Ruminococcus albus*; Elongation factor G [*Megasphaera*]; Elongation factor G - *Hallella colorans*; Elongation factor G - *Alistipes finegoldii*; Elongation factor G - Ruminococcus champanellensis; Elongation factor G - *Alistipes indistinctus*; Elongation factor G - *Prevotella bivia*; Elongation factor G - *Alistipes finegoldii*; Elongation factor G - *Prevotella stercorea*; Elongation factor G - *Prevotella buccae*; Elongation factor G - *Prevotella copri*; Elongation factor G - *Alistipes shahii*; elongation factor G [*Alistipes ihumii*]; Elongation factor G - *Alistipes inops*; Elongation factor G - *Alistipes timonensis*; Elongation factor G - *Alistipes communis*; Elongation factor G domain protein - *Roseburia intestinalis*; Elongation factor G - *Alistipes communis*; Elongation factor G - *Alistipes putredinis*; Elongation factor G - *Alistipes onderdonkii*
tr.A0A126SU96.	Ketol-acid reductoisomerase (NADP(+)) - *Bifidobacterium angulatum*; Ketol-acid reductoisomerase (NADP(+)) - *Bifidobacterium dentium*
tr.A0A1V8Q6G3.	50S ribosomal protein L27 - *Bifidobacterium dentium*; 50S ribosomal protein L27 - *Bifidobacterium catenulatum*; 50S ribosomal protein L27 - *Bifidobacterium adolescentis*
tr.WP_025579304.1.	alcohol dehydrogenase catalytic domain-containing protein [Clostridiales]; Sorbitol dehydrogenase - *Roseburia faecis*; alcohol dehydrogenase catalytic domain-containing protein - *Roseburia faecis*; Putative chlorophyll synthesis pathway protein BchC - *Blautia obeum*; Putative zinc-type alcohol dehydrogenase-like protein YjmD - *Blautia luti*; Alcohol dehydrogenase - *Hungatella hathewayi*; alcohol dehydrogenase catalytic domain-containing protein [*Blautia schinkii*]
tr.A5ZUS4.	Putative carbamoyltransferase YgeW - *Blautia obeum*
tr.A0A1G5BPU3.	Ketol-acid reductoisomerase (NADP(+)) - *Ruminococcus bromii*
tr.WP_025578097.1.	DUF4981 domain-containing protein [*Blautia wexlerae*]; Beta-galactosidase - *Roseburia faecis*; Beta-galactosidase - *Blautia obeum*; Beta-galactosidase - *Blautia luti*; glycoside hydrolase family 2 TIM barrel-domain containing protein [*Blautia schinkii*]; glycoside hydrolase family 2 TIM barrel-domain containing protein [*Blautia schinkii*]; glycoside hydrolase family 2 TIM barrel-domain containing protein [*Blautia faecis*]; glycoside hydrolase family 2 TIM barrel-domain containing protein [Eubacteriales]; glycoside hydrolase family 2 TIM barrel-domain containing protein [*Blautia glucerasea*]; glycoside hydrolase family 2 TIM barrel-domain containing protein [*Blautia*]
tr.A0A0M4LTL3.	Putative ferredoxin/ferredoxin-NADP reductase - *Bifidobacterium longum subsp. Infantis*; Ferredoxin--NADP reductase - *Bifidobacterium longum*; Ferredoxin--NADP(+) reductase2C actinobacterial (Eukaryote-like) type - *Bifidobacterium longum*; Glutamate synthase - *Bifidobacterium longum subsp. infantis*; Pyridine nucleotide-disulfide oxidoreductase - *Bifidobacterium breve*; Glutamate synthase - *Bifidobacterium scardovii*; ferredoxin--NADP reductase [*Bifidobacterium pullorum subsp. saeculare*]; Glutamate synthase - *Bifidobacterium angulatum*; Ferredoxin--NADP reductase - *Bifidobacterium bifidum*; FAD-dependent oxidoreductase [*Bifidobacterium*]; NADPH:adrenodoxin oxidoreductase - *Bifidobacterium bifidum*; Ferredoxin/ferredoxin-NADP reductase - *Bifidobacterium adolescentis*; Glutamate synthase - *Bifidobacterium catenulatum*; Glutamate synthase - *Bifidobacterium dentium*; Glutamate synthase - *Bifidobacterium catenulatum*; Glutamate synthase - Bifidobacterium dentium; Glutamate synthase - *Enterococcus hirae*
tr.A0A174US18.	OmpA family protein - *Parabacteroides distasonis*
tr.A6L1G5.	Putative outer membrane protein, probably involved in nutrient binding - *Bacteroides vulgatus*; TonB-dependent receptor - *Phocaeicola dorei*; SusC/RagA family TonB-linked outer membrane protein - *Phocaeicola dorei*
tr.A0A0M6WWS6.	Phosphoglycerate kinase - *Roseburia faecis*; Phosphoglycerate kinase [*Lacrimispora amygdalina*]; Phosphoglycerate kinase - *Roseburia hominis*
tr.WP_097005984.1.	elongation factor Ts [*Lacrimispora amygdalina*]; elongation factor Ts - *Clostridium symbiosum*; elongation factor Ts - *Clostridium symbiosum* (*Bacteroides symbiosus*); elongation factor Ts - *Hungatella xylanolytica*
tr.A0A3E3ADB3.	Phosphoenolpyruvate-protein phosphotransferase - *Erysipelatoclostridium ramosum*

Table S23. (continued)

Table S23B. (continued)

Abbreviation	Protein names
tr.WP_009268670.1.	30S ribosomal protein S2 [*Mediterraneibacter glycyrrhizinilyticus*]; 30S ribosomal protein S2 [*Mediterraneibacter glycyrrhizinilyticus*]; 30S ribosomal protein S2 - *Roseburia inulinivorans*; 30S ribosomal protein S2 - *Roseburia hominis*; 30S ribosomal protein S2 [*Merdimonas faecis*]; 30S ribosomal protein S2 - *Faecalicatena orotica*; 30S ribosomal protein S2 - *Extibacter muris*; 30S ribosomal protein S2 - *Hungatella effluvii*; 30S ribosomal protein S2 [Eubacteriales]; 30S ribosomal protein S2 - *Roseburia intestinalis*; 30S ribosomal protein S2 - *Ruminococcus bromii*; 30S ribosomal protein S2 [Eubacteriales]; ribosomal protein S2 [*Megasphaera micronuciformis*]; 30S ribosomal protein S2 - *Enterocloster aldenensis*; 30S ribosomal protein S2 - Clostridium chromiireducens; 30S ribosomal protein S2 [*Clostridium*]; 30S ribosomal protein S2 - *Enterocloster asparagiformis*; 30S ribosomal protein S2 - *Enterocloster aldenensis*; 30S ribosomal protein S2 - *Roseburia faecis*; 30S ribosomal protein S2 - *Megasphaera elsdenii*; 30S ribosomal protein S2 [*Blautia schinkii*]; 30S ribosomal protein S2 - *Flavonifractor plautii* (*Fusobacterium plautii*); 30S ribosomal protein S2 - *Blautia luti*; 30S ribosomal protein S2 - *Flavonifractor plautii* (*Fusobacterium plautii*); 30S ribosomal protein S2 [Eubacteriales]; 30S ribosomal protein S2 - Clostridium perfringens; 30S ribosomal protein S2 - *Phascolarctobacterium succinatutens*; 30S ribosomal protein S2 - *Blautia faecicola*; 30S ribosomal protein S2 - *Ruminococcus albus*; 30S ribosomal protein S2 - *Phascolarctobacterium faecium*; 30S ribosomal protein S2 - *Phascolarctobacterium faecium*; 30S ribosomal protein S2 - *Subdoligranulum variabile*; 30S ribosomal protein S2 - *Clostridium septicum*
tr.F3PHE4.	10 kDa chaperonin - *Bacteroides clarus*; 10 kDa chaperonin - *Bacteroides stercoris*; Co-chaperonin GroES - *Bacteroides faecis*; Co-chaperonin GroES - *Bacteroides eggerthii*; Co-chaperonin GroES - *Bacteroides caccae*; Co-chaperonin GroES - *Bacteroides uniformis*; Co-chaperonin GroES - *Bacteroides cellulosilyticus*; Co-chaperonin GroES - *Bacteroides thetaiotaomicron*; Co-chaperonin GroES - *Bacteroides thetaiotaomicron*; Co-chaperonin GroES - *Phocaeicola plebeius*; Co-chaperonin GroES - *Bacteroides oleiciplenus*; Co-chaperonin GroES - *Bacteroides fragilis*; Co-chaperonin GroES - *Bacteroides fragilis*;
tr.A0A4S5B9L3.	Extracellular solute-binding protein - *Bifidobacterium longum subsp. Infantis*; Family 1 extracellular solute-binding protein - *Bifidobacterium stercoris*
tr.A7AIR2.	Uncharacterized protein - *Parabacteroides merdae*; Uncharacterized protein - *Parabacteroides merdae*
tr.WP_008703889.1.	anaerobic carbon-monoxide dehydrogenase catalytic subunit [Clostridiales]
tr.WP_195360800.1.	DUF5458 family protein [*Phocaeicola massiliensis*]
tr.D1PG79.	Fumarate reductase/succinate dehydrogenase flavoprotein subunit - *Prevotella copri*; Succinate dehydrogenase or fumarate reductase, flavoprotein subunit - *Phocaeicola coprocola*; Fumarate reductase/succinate dehydrogenase flavoprotein subunit - *Phocaeicola coprophilus*; Succinate dehydrogenase or fumarate reductase, flavoprotein subunit - *Prevotella buccae*; Succinate dehydrogenase or fumarate reductase, flavoprotein subunit - *Phocaeicola plebeius*; Succinate dehydrogenase or fumarate reductase, flavoprotein subunit - *Prevotella bivia*
tr.A0A126SV93.	Antigen 84 - *Bifidobacterium angulatum*
tr.A0A2Y9BGR4.	50S ribosomal protein L19 - *Faecalicatena orotica*; 50S ribosomal protein L19 [*Blautia glucerasea*]; 50S ribosomal protein L19 - *Extibacter muris*; 50S ribosomal protein L19 - *Lachnospira pectinoschiza*; 50S ribosomal protein L19 - *Roseburia intestinalis*
tr.A0A0A1GQP2.	Glutamate dehydrogenase - *Bifidobacterium longum*; Glutamate dehydrogenase - *Bifidobacterium longum subsp.* Infantis; Glutamate dehydrogenase - *Bifidobacterium longum subsp. Infantis*
tr.WP_028254790.1.	methylmalonyl-CoA mutase family protein [*Veillonella magna*]
tr.C7GD05.	50S ribosomal protein L3 - *Roseburia intestinalis*; 50S ribosomal protein L3 - *Roseburia inulinivorans*; 50S ribosomal protein L3 [*Blautia*]; 50S ribosomal protein L3 - *Blautia faecicola*; 50S ribosomal protein L3 [Eubacteriales]; 50S ribosomal protein L3 - *Extibacter muris*; 50S ribosomal protein L3 [*Mediterraneibacter glycyrrhizinilyticus*]; 50S ribosomal protein L3 - *Roseburia hominis*; 50S ribosomal protein L3 - *Faecalicatena orotica*
tr.WP_156902361.1.	formate C-acetyltransferase [*Ruminococcus callidus*]; formate C-acetyltransferase [*Ruminococcus*]; formate C-acetyltransferase [*Ruminococcus callidus*]
tr.WP_025577065.1.	acetyl-CoA decarbonylase/synthase complex subunit gamma [Clostridiales]; acetyl-CoA decarbonylase/synthase complex subunit gamma [Clostridiales]; acetyl-CoA decarbonylase/synthase complex subunit gamma [*Blautia glucerasea*]; acetyl-CoA decarbonylase/synthase complex subunit gamma [Eubacteriales]; acetyl-CoA decarbonylase/synthase complex subunit gamma [Eubacteriales]; CO dehydrogenase/acetyl-CoA synthase delta subunit - *Blautia obeum*; Corrinoid/iron-sulfur protein large subunit - *Blautia luti*
tr.D4MV01.	NADH peroxidase - *Anaerostipes hadrus*
tr.D4MZ60.	Inosine-5-monophosphate dehydrogenase - *Anaerostipes hadrus*; Inosine-5-monophosphate dehydrogenase - *Roseburia hominis*; Inosine-5'-monophosphate dehydrogenase - *Eisenbergiella massiliensis*; Inosine-5'-monophosphate dehydrogenase - *Flavonifractor plautii* (*Fusobacterium plautii*)
tr.A0A174D9I7.	Arginine-binding extracellular protein ArtP - *Anaerostipes hadrus*

Table S23. (continued)

Table S23B. (continued)

Abbreviation	Protein names
tr.A6KXV7.	Putative outer membrane protein, probably involved in nutrient binding - *Bacteroides vulgatus*; Membrane protein - Phocaeicola dorei; SusC/RagA family TonB-linked outer membrane protein - *Phocaeicola dorei*; Putative outer membrane protein, probably involved in nutrient binding - *Phocaeicola vulgatus*; SusC/RagA family TonB-linked outer membrane protein - *Phocaeicola dorei*; SusC/RagA family TonB-linked outer membrane protein - *Parabacteroides goldsteinii*; SusC/RagA family TonB-linked outer membrane protein - *Parabacteroides goldsteinii*
tr.A0A399IH89.	Probable transaldolase - *Clostridium chromiireducens*; Probable transaldolase - *Clostridium chromiireducens*; Probable transaldolase - *Clostridium chromiireducens*; Fructose-6-phosphate aldolase - *Clostridium butyricum*; Probable transaldolase - Clostridium *chromiireducens*; Probable transaldolase - *Clostridium disporicum*; Probable transaldolase - *Clostridium butyricum*; Probable transaldolase - *Clostridium butyricum*; Probable transaldolase - *Clostridium saccharobutylicum*; Probable transaldolase - *Enterocloster asparagiformis*; Probable transaldolase - *Enterocloster lavalensis*; Probable transaldolase - *Enterocloster lavalensis*; Probable transaldolase - *Clostridium saccharobutylicum*
tr.A0A6P1YTK1.	Acetyl-CoA decarbonylase/synthase complex subunit delta - *Blautia producta*; Acetyl-CoA decarbonylase/synthase complex subunit delta - *Blautia faecicola*
tr.D4MUZ9.	FAD/FMN-containing dehydrogenases - *Anaerostipes hadrus*; FAD-binding oxidoreductase - *Anaerostipes hadrus*; Glycolate oxidase - *Faecalicatena orotica*; 2-hydroxy-acid oxidase - *Clostridium septicum*; FAD-binding protein - *Clostridium chromiireducens*; Putative FAD-linked oxidoreductase - *Clostridium saccharobutylicum*; FAD-binding oxidoreductase - *Enterocloster aldenensis*; FAD-binding oxidoreductase - *Enterocloster aldenensis*
tr.D4MXD7.	Glutamate dehydrogenase - *Anaerostipes hadrus*
tr.A0A087DMJ5.	60 kDa chaperonin - *Bifidobacterium stercoris*
tr.D4N113.	Inosose isomerase - *Anaerostipes hadrus*; Inosose isomerase - *Anaerostipes hadrus*; sugar phosphate isomerase/epimerase family protein [Eubacteriales]; sugar phosphate isomerase/epimerase family protein [*Blautia schinkii*]; Inosose isomerase - *Blautia luti*
tr.D4BQK2.	Phosphate-binding protein PstS - *Bifidobacterium breve*
tr.A0A1V8Q531.	50S ribosomal protein L2 - *Bifidobacterium dentium*; 50S ribosomal protein L2 - *Bifidobacterium stercoris*; 50S ribosomal protein L2 [*Bifidobacterium*]; 50S ribosomal protein L2 [*Bifidobacterium pullorum subsp. Saeculare*]; 50S ribosomal protein L2 - *Bifidobacterium scardovii*; 50S ribosomal protein L2 - *Bifidobacterium catenulatum*
tr.WP_025580805.1.	ATP-dependent chaperone ClpB [*Blautia wexlerae*]; Chaperone protein ClpB - *Blautia obeum*
tr.D4MUG4.	50S ribosomal protein L7/L12 - *Anaerostipes hadrus*; 50S ribosomal protein L7/L12 [*Lachnospira*]; 50S ribosomal protein L12 - *Lachnospira pectinoschiza*; 50S ribosomal protein L7/L12 [*Mediterraneibacter glycyrrhizinilyticus*]; 50S ribosomal protein L7/L12 [*Mediterraneibacter glycyrrhizinilyticus*]
tr.A0A133PIY4.	30S ribosomal protein S2 - *Lactobacillus gasseri*

Table S23. (continued)

Table S23B. (continued)

Abbreviation	Protein names
tr.WP_097006856.1.	sn-glycerol-3-phosphate ABC transporter ATP-binding protein UgpC [*Lacrimispora amygdalina*]; ABC transporter - *Enterocloster clostridioformis*; sn-glycerol-3-phosphate ABC transporter ATP-binding protein UgpC [*Lachnospira*]; Carbohydrate ABC transporter ATP-binding protein, CUT1 family - *Lachnospira pectinoschiza*; sn-glycerol-3-phosphate ABC transporter ATP-binding protein UgpC - *Enterocloster aldenensis*; ABC transporter - *Hungatella hathewayi*; Carbohydrate ABC transporter ATP-binding protein (CUT1 family) - *Hungatella xylanolytica*; Carbohydrate ABC transporter ATP-binding protein (CUT1 family) - *Hungatella effluvii*; Carbohydrate ABC transporter ATP-binding protein, CUT1 family - *Enterocloster citroniae*; ABC transporter, ATP-binding protein - *Streptococcus parasanguinis*; sn-glycerol-3-phosphate ABC transporter ATP-binding protein UgpC - *Enterocloster asparagiformis*; sn-glycerol-3-phosphate ABC transporter ATP-binding protein UgpC [*Streptococcus lactarius*]; ABC transporter, ATP-binding protein - *Streptococcus sanguinis*; sn-glycerol-3-phosphate import ATP-binding protein UgpC - *Lachnospira pectinoschiza*; sn-glycerol-3-phosphate ABC transporter ATP-binding protein UgpC - *Erysipelatoclostridium ramosum*; sn-glycerol-3-phosphate ABC transporter ATP-binding protein UgpC - *Thomasclavelia ramosa*; Multiple sugar ABC transporter, ATP-binding protein - *Streptococcus gordonii*; Multiple sugar ABC transporter, ATP-binding protein - *Streptococcus sinensis*; ABC transporter ATP-binding protein - *Clostridium paraputrificum*; ABC transporter ATP-binding protein - *Streptococcus alactolyticus*; sn-glycerol-3-phosphate ABC transporter ATP-binding protein UgpC - *Streptococcus rubneri*; ABC transporter ATP-binding protein - *Streptococcus salivarius*; Sugar ABC transporter ATP-binding protein - *Massilimicrobiota timonensis*; sn-glycerol-3-phosphate ABC transporter ATP-binding protein UgpC - *Streptococcus alactolyticus*; Multiple sugar ABC transporter, ATP-binding protein - *Streptococcus sinensis*; ABC transporter, ATP-binding protein - *Streptococcus gordonii*; sn-glycerol-3-phosphate ABC transporter ATP-binding protein UgpC [*Blautia schinkii*]; ABC transporter ATP-binding protein - *Enterocloster lavalensis*; ABC transporter, ATP-binding protein - *Streptococcus equinus*; Trehalose import ATP-binding protein SugC - *Collinsella aerofaciens*; Trehalose import ATP-binding protein SugC - *Collinsella aerofaciens*; Trehalose import ATP-binding protein SugC - *Clostridium chromiireducens*; sn-glycerol-3-phosphate ABC transporter ATP-binding protein UgpC - *Clostridium chromiireducens*; sn-glycerol-3-phosphate ABC transporter ATP-binding protein UgpC [*Blautia schinkii*]; Carbohydrate ABC transporter ATP-binding protein, CUT1 family - *Enterocloster lavalensis*; Carbohydrate ABC transporter ATP-binding protein, CUT1 family - *Enterocloster lavalensis*; sn-glycerol-3-phosphate ABC transporter ATP-binding protein UgpC - *Enterococcus avium* (*Streptococcus avium*); Maltose ABC transporter - *Clostridium disporicum*; ABC transporter, ATP-binding protein - *Blautia obeum*; ABC transporter ATP-binding protein - *Enterococcus casseliflavus* (*Enterococcus flavescens*); sn-glycerol-3-phosphate ABC transporter ATP-binding protein UgpC - *Enterococcus avium* (*Streptococcus avium*); Multiple sugar-binding transport ATP-binding protein MsmK - *Enterococcus raffinosus*; Sugar ABC transporter ATP-binding protein - *Enterococcus avium* (*Streptococcus avium*); ABC transporter ATP-binding protein - *Enterococcus faecalis* (*Streptococcus faecalis*); Trehalose import ATP-binding protein SugC - *Collinsella aerofaciens*; Sugar ABC transporter ATP-binding protein - *Enterococcus avium* (*Streptococcus avium*); sn-glycerol-3-phosphate ABC transporter ATP-binding protein UgpC - *Enterococcus avium* (*Streptococcus avium*); Carbohydrate ABC transporter ATP-binding protein, CUT1 family - *Enterocloster lavalensis*; ABC transporter ATP-binding protein - *Enterocloster asparagiformis*; ABC transporter ATP-binding protein - *Enterocloster lavalensis*; ABC transporter ATP-binding protein [*Beduini massiliensis*]; ABC transporter ATP-binding protein - *Clostridium symbiosum* (*Bacteroides symbiosus*); ABC transporter ATP-binding protein - *Clostridium symbiosum* (*Bacteroides symbiosus*); Trehalose import ATP-binding protein SugC - *Clostridium saccharobutylicum*; Multiple sugar-binding transport ATP-binding protein MsmK - *Enterococcus devriesei*; ABC transporter ATP-binding protein - *Enterococcus gallinarum*; sn-glycerol-3-phosphate ABC transporter ATP-binding protein UgpC - *Enterococcus hirae*; Maltose ABC transporter - *Clostridium disporicum*; Multiple sugar-binding transport ATP-binding protein MsmK - *Enterococcus raffinosus*; ABC transporter ATP-binding protein - *Enterococcus dispar*; sn-glycerol-3-phosphate ABC transporter ATP-binding protein UgpC [*Clostridium*]; sn-glycerol-3-phosphate ABC transporter ATP-binding protein UgpC [*Clostridium tertium*]; ABC transporter ATP-binding protein - *Enterococcus devriesei*; Trehalose import ATP-binding protein SugC - *Clostridium chromiireducens*; Multiple sugar-binding transport ATP-binding protein MsmK - *Enterococcus gallinarum*; Multiple sugar-binding transport ATP-binding protein MsmK - *Enterococcus devriesei*; ABC transporter ATP-binding protein - *Enterococcus hirae*; Sugar ABC transporter ATP-binding protein - *Clostridium septicum*; ABC transporter ATP-binding protein - *Enterococcus casseliflavus* (*Enterococcus flavescens*); ABC transporter ATP-binding protein - *Enterocloster clostridioformis*
tr.D4MYG8.	50S ribosomal protein L2 - *Anaerostipes hadrus*
tr.C7G6S2.	50S ribosomal protein L7/L12 - *Roseburia intestinalis*
tr.A0A2V2FBJ8.	Phosphoglycerate kinase - *Subdoligranulum variabile*; Phosphoglycerate kinase - *Gemmiger formicilis*
tr.WP_173767105.1.	transcription elongation factor GreA [Clostridiales]; transcription elongation factor GreA [Clostridiales]
tr.WP_173773355.1.	IMP cyclohydrolase [Clostridiales]; IMP cyclohydrolase [Clostridiales]; IMP cyclohydrolase [Eubacteriales]; IMP cyclohydrolase [*Lachnoclostridium pacaense*]; IMP cyclohydrolase-like protein - *Ruminococcus champanellensis*; IMP cyclohydrolase - *Blautia luti*; IMP cyclohydrolase - *Enterocloster aldenensis*; IMP cyclohydrolase - *Enterocloster aldenensis*; IMP cyclohydrolase-like protein - *Enterocloster citroniae*

Table S23. (continued)

Table S23B. (continued)

**Abbreviation**	**Protein names**
tr.WP_020993443.1.	30S ribosomal protein S6 [Clostridiales]; 30S ribosomal protein S6 [*Blautia glucerasea*]; 30S ribosomal protein S6 [Eubacteriales]
tr.A0A6P1Z4H2.	50S ribosomal protein L5 - *Blautia producta*; 50S ribosomal protein L5 - *Clostridium symbiosum*; 50S ribosomal protein L5 - *Roseburia faecis*
tr.A0A6P1Z6F1.	Pyruvate, phosphate dikinase - *Blautia producta*
tr.A0A4V2X185.	SusC/RagA family TonB-linked outer membrane protein - *Phocaeicola dorei*; Putative outer membrane protein, probably involved in nutrient binding - *Bacteroides vulgatus*; TonB-dependent receptor [*Phocaeicola sartorii*]; carboxypeptidase-like regulatory domain-containing protein, partial [*Phocaeicola*]
tr.D4MYK4.	4Fe-4S dicluster domain-containing protein - *Anaerostipes hadrus*
tr.WP_147600180.1.	pyruvate, phosphate dikinase [*Blautia caecimuris*]
tr.A0A2Y9B7L8.	Nitrogen fixation NifU-like protein - *Faecalicatena orotica*; Fe-S cluster assembly scaffold protein NifU - *Anaerostipes hadrus*; Fe-S iron-sulfur cluster assembly protein, NifU family - *Blautia obeum*; Fe-S cluster assembly scaffold protein NifU - *Blautia faecicola*; Fe-S cluster assembly scaffold protein NifU [*Merdimonas faecis*]; Fe-S cluster assembly scaffold protein NifU [*Mediterraneibacter glycyrrhizinilyticus*]
tr.A0A6P1Z1I4.	sn-glycerol-3-phosphate ABC transporter ATP-binding protein UgpC - *Blautia producta*
tr.WP_101723696.1.	IMP dehydrogenase [*Eggerthella timonensis*]; IMP dehydrogenase - *Eggerthella lenta*
tr.D4MZK6.	Enoyl-CoA hydratase - *Anaerostipes hadrus*; Enoyl-CoA hydratase - *Eisenbergiella porci*; Enoyl-CoA hydratase - *Eisenbergiella massiliensis*; Putative enoyl-CoA hydratase echA8 - *Eisenbergiella tayi*
tr.A0A1V8PLT8.	DNA-directed RNA polymerase subunit beta - *Bifidobacterium catenulatum*; DNA-directed RNA polymerase subunit beta - *Bifidobacterium catenulatum*; DNA-directed RNA polymerase subunit beta - *Bifidobacterium dentium*; DNA-directed RNA polymerase subunit beta' [*Bifidobacterium pullorum*]; DNA-directed RNA polymerase subunit beta' [*Bifidobacterium pullorum*]; DNA-directed RNA polymerase subunit beta [*Bifidobacterium pullorum subsp. saeculare*]; DNA-directed RNA polymerase subunit beta - *Bifidobacterium animalis subsp. lactis* (*Bifidobacterium lactis*); DNA-directed RNA polymerase subunit beta - *Bifidobacterium animalis subsp. lactis* (*Bifidobacterium lactis*); RpoC, partial [*Bifidobacterium pullorum subsp. saeculare*]
tr.WP_027431932.1.	class II fructose-1,6-bisphosphate aldolase [*Lachnospira*]; Fructose-bisphosphate aldolase, class II - *Lachnospira pectinoschiza*
tr.D4MUH3.	DNA-directed RNA polymerase subunit beta - *Anaerostipes hadrus*; DNA-directed RNA polymerase subunit beta - *Anaerostipes hadrus*
tr.A6L1X3.	Putative outer membrane protein, probably involved in nutrient binding - *Bacteroides vulgatus*; SusC/RagA family TonB-linked outer membrane protein - *Phocaeicola dorei*; SusC/RagA family TonB-linked outer membrane protein - *Phocaeicola dorei*
tr.D4MW58.	Phosphate ABC transporter substrate-binding protein - *Anaerostipes hadrus*; Phosphate ABC transporter substrate-binding protein - *Anaerostipes hadrus*
tr.A0A0M3T6V1.	1,4-alpha-glucan branching enzyme GlgB - *Bifidobacterium longum subsp. Infantis*; 1,4-alpha-glucan branching enzyme GlgB - *Bifidobacterium longum subsp. Infantis*; 1,4-alpha-glucan branching enzyme GlgB - *Bifidobacterium longum*; 1,4-alpha-glucan branching enzyme GlgB - *Bifidobacterium longum*
tr.A0A0M4HKR2.	Enolase - *Streptococcus thermophilus*; Phosphopyruvate hydratase (Fragment) - *Streptococcus salivarius*; Enolase - *Streptococcus parasanguinis*; Enolase - *Streptococcus rubneri*; Enolase - *Streptococcus sinensis*; Enolase - *Streptococcus equinus*; surface-displayed alpha-enolase [*Streptococcus lactarius*]; Enolase - *Streptococcus sanguinis*; Enolase - *Streptococcus salivarius*; Enolase - *Streptococcus gordonii*; Enolase - *Enterococcus hirae*; Enolase - *Enterococcus hirae*
sp.P69780.	Major outer membrane lipoprotein Lpp - *Shigella flexneri*; Major outer membrane lipoprotein Lpp - *Escherichia coli*; Major outer membrane lipoprotein Lpp - *Escherichia fergusonii*; Major outer membrane lipoprotein Lpp - *Klebsiella variicola*; Major outer membrane lipoprotein Lpp - *Escherichia fergusonii*; Major outer membrane lipoprotein Lpp - *Enterobacter mori*; Major outer membrane lipoprotein Lpp - *Klebsiella grimontii*; Major outer membrane lipoprotein Lpp - *Shigella flexneri*; Major outer membrane lipoprotein Lpp - *Klebsiella quasipneumoniae*; Major outer membrane lipoprotein Lpp - *Klebsiella pneumoniae*; Major outer membrane lipoprotein Lpp - *Shigella sonnei*; Major outer membrane lipoprotein Lpp - *Klebsiella oxytoca*; Major outer membrane lipoprotein Lpp - *Klebsiella aerogenes* (*Enterobacter aerogenes*); Major outer membrane lipoprotein Lpp - *Enterobacter cloacae*
tr.A0A679HBI1.	Superoxide dismutase - *Bacteroides thetaiotaomicron*; Superoxide dismutase - *Bacteroides xylanisolvens*; Superoxide dismutase - *Bacteroides fragilis*; Superoxide dismutase - *Bacteroides ovatus*; Superoxide dismutase - *Bacteroides caccae*; Superoxide dismutase - *Bacteroides caecimuris*; Superoxide dismutase - *Bacteroides thetaiotaomicron*; Superoxide dismutase - *Bacteroides caecimuris*; Superoxide dismutase - *Bacteroides faecis*; Superoxide dismutase - *Phocaeicola vulgatus*; Superoxide dismutase - *Bacteroides salyersiae*; Superoxide dismutase - *Phocaeicola dorei*; Superoxide dismutase - *Bacteroides fragilis*; Superoxide dismutase - *Bacteroides clarus*; Superoxide dismutase - *Bacteroides eggerthii*; Superoxide dismutase - *Bacteroides stercoris*; Superoxide dismutase - *Bacteroides eggerthii*; Superoxide dismutase - *Bacteroides uniformis*

Table S23. (continued)

<u>Table S23B.</u> (continued)

Abbreviation	Protein names
tr.WP_033508353.1.	30S ribosomal protein S10 [*Bifidobacterium*]; 30S ribosomal protein S10 [*Terrabacteria group*]; 30S ribosomal protein S10 [*Bifidobacterium pullorum subsp. Saeculare*]; 30S ribosomal protein S10 - *Bifidobacterium breve*; 30S ribosomal protein S10 - *Bifidobacterium animalis subsp. lactis* (*Bifidobacterium lactis*); 30S ribosomal protein S10 - *Bifidobacterium dentium*; 30S ribosomal protein S10 - *Bifidobacterium catenulatum*; 30S ribosomal protein S10 - *Bifidobacterium angulatum*; 30S ribosomal protein S10 - *Bifidobacterium longum subsp. infantis*; 30S ribosomal protein S10 - *Bifidobacterium bifidum*; 30S ribosomal protein S10 [*Bifidobacterium pullorum subsp. saeculare*]; 30S ribosomal protein S10 - *Bifidobacterium scardovii*; 30S ribosomal protein S10 - *Bifidobacterium longum*
tr.A6L1G6.	Putative outer membrane protein, probably involved in nutrient binding - *Bacteroides vulgatus*; RagB/SusD family nutrient uptake outer membrane protein - *Phocaeicola dorei*;
tr.A0A0M4LFR2.	30S ribosomal protein S13 - *Bifidobacterium longum subsp. Infantis*; 30S ribosomal protein S13 - *Bifidobacterium longum*; 30S ribosomal protein S13 - *Bifidobacterium breve*; 30S ribosomal protein S13 - *Bifidobacterium animalis subsp. lactis* (*Bifidobacterium lactis*)
tr.A0A0H2PWI8.	Trigger factor - *Bifidobacterium bifidum*; Trigger factor - *Bifidobacterium bifidum*
tr.D4MW30.	Bacterial Ig-like domain (Group 2) - *Anaerostipes hadrus*; Uncharacterized protein - *Anaerostipes hadrus*
tr.D4BLH5.	Alkyl hydroperoxide reductase C - *Bifidobacterium breve*; Alkyl hydroperoxide reductase C - *Bifidobacterium catenulatum*
tr.A0A2U0C039.	30S ribosomal protein S3 - *Bifidobacterium bifidum*
tr.WP_195609585.1.	glutamine synthetase III [*Mediterraneibacter glycyrrhizinilyticus*]; glutamine synthetase - *Faecalicatena orotica*
tr.A0A329TW61.	Uronate isomerase - *Faecalibacterium prausnitzii*
tr.A0A415MLA0.	Uncharacterized protein - *Parabacteroides distasonis*
tr.WP_119239650.1.	2,3-bisphosphoglycerate-independent phosphoglycerate mutase [Clostridiales]; 2,3-bisphosphoglycerate-independent phosphoglycerate mutase - *Blautia luti*
tr.A0A6P1YWQ6.	Phosphoenolpyruvate carboxykinase (ATP) - *Blautia producta*; Phosphoenolpyruvate carboxykinase (ATP) - *Faecalicatena orotica*; Phosphoenolpyruvate carboxykinase (ATP) - *Clostridium symbiosum* (*Bacteroides symbiosus*); Phosphoenolpyruvate carboxykinase (ATP) [*Lacrimispora amygdalina*]
tr.A6KWT3.	Putative outer membrane protein, probably involved in nutrient binding - *Bacteroides vulgatus*
tr.D4N0D1.	5-methyltetrahydropteroyltriglutamate--homocysteine S-methyltransferase - *Anaerostipes hadrus*; 5-methyltetrahydropteroyltriglutamate--homocysteine S-methyltransferase - *Ruminococcus bromii*
tr.WP_148461930.1.	phosphoenolpyruvate carboxykinase (ATP) [Clostridiales]
tr.A0A1V8Q6B2.	30S ribosomal protein S15 - *Bifidobacterium dentium*; 30S ribosomal protein S15 - *Bifidobacterium catenulatum*; 30S ribosomal protein S15 - *Bifidobacterium adolescentis*
tr.D4LD40.	Sulfide dehydrogenase (Flavoprotein) subunit SudA - *Ruminococcus champanellensis*; NADPH-dependent glutamate synthase [*Lachnospira*]; Sulfide dehydrogenase (Flavoprotein) subunit SudA - *Lachnospira pectinoschiza*; Glutamate synthase (NADPH), homotetrameric - *Clostridium paraputrificum*; NADPH-dependent glutamate synthase [*Ruminococcus*]; NADPH-dependent glutamate synthase [*Ruminococcus callidus*]; NADPH-dependent glutamate synthase - *Clostridium symbiosum* (*Bacteroides symbiosus*); NADPH-dependent glutamate synthase - Clostridium symbiosum (*Bacteroides symbiosus*); Glutamate synthase [*Ruminococcus callidus*]
tr.WP_005427721.1.	BMC domain-containing protein [Clostridiales]; BMC domain protein - *Blautia obeum*; Propanediol utilization protein PduA - *Blautia luti*; BMC domain-containing protein, partial [*Blautia glucerasea*]; BMC domain-containing protein - *Eisenbergiella massiliensis*; Ethanolamine utilization protein EutM - *Eisenbergiella tayi*; Propanediol utilization protein PduA - *Eisenbergiella tayi*
tr.A6L1G4.	Putative outer membrane protein, probably involved in nutrient binding - *Bacteroides vulgatus*; RagB/SusD family nutrient uptake outer membrane protein - *Phocaeicola dorei*; RagB/SusD family nutrient uptake outer membrane protein - *Phocaeicola dorei*; RagB/SusD family nutrient uptake outer membrane protein [*Phocaeicola sartorii*]
tr.E5XXC7.	Alanine--tRNA ligase - *Bifidobacterium longum*; Alanine--tRNA ligase - *Bifidobacterium longum*
tr.A0A0M6WFJ8.	3-hydroxybutyryl-CoA dehydrogenase - *Roseburia faecis*
tr.WP_142690962.1.	NADP-specific glutamate dehydrogenase [*Clostridium*]; NADP-specific glutamate dehydrogenase [*Clostridium*]; Glutamate dehydrogenase - *Clostridium disporicum*; NADP-specific glutamate dehydrogenase [*Clostridium tertium*]
tr.D4MYK3.	Methionine synthase II (Cobalamin-independent) - *Anaerostipes hadrus*; 5-methyltetrahydropteroyltriglutamate--homocysteine S-methyltransferase - *Anaerostipes hadrus*
tr.A6L7K3.	Major outer membrane protein OmpA - *Bacteroides vulgatus*

Table S23. (continued)

Table S23B. (continued)

Abbreviation	Protein names
tr.WP_025577186.1.	polyribonucleotide nucleotidyltransferase [Clostridiales]; polyribonucleotide nucleotidyltransferase - *Blautia producta*; polyribonucleotide nucleotidyltransferase [*Blautia schinkii*]; polyribonucleotide nucleotidyltransferase [Eubacteriales]; polyribonucleotide nucleotidyltransferase - *Blautia luti*; polyribonucleotide nucleotidyltransferase [*Blautia*]; polyribonucleotide nucleotidyltransferase [*Lacrimispora amygdalina*]; polyribonucleotide nucleotidyltransferase - *Roseburia intestinalis*; polyribonucleotide nucleotidyltransferase [*Lacrimispora amygdalina*]; polyribonucleotide nucleotidyltransferase, partial [*Blautia schinkii*]; polyribonucleotide nucleotidyltransferase - *Blautia obeum*; polyribonucleotide nucleotidyltransferase - *Hungatella xylanolytica*; polyribonucleotide nucleotidyltransferase - *Anaerostipes hadrus*; polyribonucleotide nucleotidyltransferase [*Blautia*]; polyribonucleotide nucleotidyltransferase [Eubacteriales]; polyribonucleotide nucleotidyltransferase - *Enterocloster asparagiformis*; polyribonucleotide nucleotidyltransferase - *Enterocloster aldenensis*; Polyribonucleotide nucleotidyltransferase - *Blautia faecicola*; polyribonucleotide nucleotidyltransferase - *Clostridium symbiosum* (*Bacteroides symbiosus*); Polyribonucleotide nucleotidyltransferase - *Clostridium symbiosum* (*Bacteroides symbiosus*); polyribonucleotide nucleotidyltransferase - *Roseburia faecis*; S1 RNA-binding domain-containing protein, partial [*Blautia schinkii*]; Polyribonucleotide nucleotidyltransferase - *Roseburia hominis*; polyribonucleotide nucleotidyltransferase [Eubacteriales]; polyribonucleotide nucleotidyltransferase - *Eisenbergiella porci*; polyribonucleotide nucleotidyltransferase - *Roseburia inulinivorans*; polyribonucleotide nucleotidyltransferase - *Lachnospira pectinoschiza*; polyribonucleotide nucleotidyltransferase [*Lachnospira multipara*]; polyribonucleotide nucleotidyltransferase - *Lachnospira pectinoschiza*; polyribonucleotide nucleotidyltransferase - *Faecalicatena orotica*; polyribonucleotide nucleotidyltransferase - *Eisenbergiella tayi*; Polyribonucleotide nucleotidyltransferase - *Enterocloster aldenensis*; polyribonucleotide nucleotidyltransferase - *Eisenbergiella massiliensis*; polyribonucleotide nucleotidyltransferase - *Hungatella effluvii*
tr.WP_148461524.1	ABC transporter substrate-binding protein [Clostridiales]; ABC transporter substrate-binding protein [Clostridiales]; ABC transporter substrate-binding protein [Eubacteriales]
tr.WP_187565966.1.	knotted carbamoyltransferase YgeW [*Blautia faecis*]; knotted carbamoyltransferase YgeW [Clostridiales]
tr.A0A2N0SUC3.	Sugar-binding protein - *Bifidobacterium longum*; ABC superfamily ATP binding cassette transporter, solute-binding protein - *Bifidobacterium longum subsp. Infantis*; Bacterial extracellular solute-binding protein - *Bifidobacterium longum*; Putative arabinose-binding protein - *Bifidobacterium longum subsp. infantis*
tr.A0A0H2P4J2.	Elongation factor Ts - *Bifidobacterium bifidum*
tr.A0A151C3V8.	ABC transporter substrate-binding protein - *Bifidobacterium longum*; ABC transporter, substrate-binding protein, family 5 - *Bifidobacterium breve*
tr.A0A6N7WKN0.	Electron transfer flavoprotein subunit alpha/FixB family protein - *Eisenbergiella tayi*; Electron transfer flavoprotein subunit alpha/FixB family protein - *Eisenbergiella massiliensis*; Acryloyl-CoA reductase electron transfer subunit beta - *Eisenbergiella tayi*; Electron transfer flavoprotein subunit alpha/FixB family protein [*Lacrimispora amygdalina*]; Electron transfer flavoprotein subunit alpha/FixB family protein - *Clostridium symbiosum* (*Bacteroides symbiosus*)
tr.D4MZD8.	LL-diaminopimelate aminotransferase - *Anaerostipes hadrus*; LL-diaminopimelate aminotransferase [*Blautia faecis*]; LL-diaminopimelate aminotransferase [Eubacteriales]; LL-diaminopimelate aminotransferase [*Blautia glucerasea*]; LL-diaminopimelate aminotransferase [Eubacteriales]; LL-diaminopimelate aminotransferase [*Merdimonas faecis*]; LL-diaminopimelate aminotransferase - *Roseburia hominis*; LL-diaminopimelate aminotransferase - *Roseburia inulinivorans*
tr.D4N105.	Inositol 2-dehydrogenase - *Anaerostipes hadrus*
tr.WP_070088499.1.	elongation factor G [*Merdimonas faecis*]; elongation factor G - *Faecalicatena orotica*
tr.WP_187565034.1.	substrate-binding domain-containing protein [*Blautia faecis*]; substrate-binding domain-containing protein [Clostridiales]; substrate-binding domain-containing protein [Eubacteriales]; substrate-binding domain-containing protein [Eubacteriales]; substrate-binding domain-containing protein [*Blautia wexlerae*]
tr.A0A0A1GN56.	ANTAR domain-containing protein - *Bifidobacterium longum*; Response regulator with RNA-binding domain - *Bifidobacterium longum*; Response regulator - *Bifidobacterium longum subsp. Infantis*; Putative transcriptional regulatory protein pdtaR - *Bifidobacterium longum subsp. infantis*; response regulator [*Bifidobacterium pullorum*]; response regulator [*Bifidobacterium pullorum*]; putative response regulator receiver domain protein [*Bifidobacterium pullorum subsp. saeculare*]; Two-component system response regulator - *Bifidobacterium scardovii*
tr.F0FPW5.	Glyceraldehyde-3-phosphate dehydrogenase - *Streptococcus sanguinis*; Glyceraldehyde-3-phosphate dehydrogenase - *Streptococcus salivarius*; Glyceraldehyde-3-phosphate dehydrogenase - *Streptococcus thermophilus*; Glyceraldehyde-3-phosphate dehydrogenase - *Streptococcus salivarius*
tr.D4BPL1.	WYL domain-containing protein - *Bifidobacterium breve*; WYL domain-containing protein - *Bifidobacterium longum*; WYL domain protein - *Bifidobacterium longum subsp. Infantis*

Table S23. (continued)

Table S23B. (continued)

Abbreviation	Protein names
tr.A0A087DM86.	Proline--tRNA ligase - *Bifidobacterium stercoris*; Proline--tRNA ligase - *Bifidobacterium scardovii*; Proline--tRNA ligase - *Bifidobacterium longum*; proline--tRNA ligase [*Bifidobacterium pullorum*]; proline--tRNA ligase [*Bifidobacterium pullorum*]; Proline--tRNA ligase - *Bifidobacterium breve*; Proline--tRNA ligase - Bifidobacterium bifidum; prolyl-tRNA synthetase [*Bifidobacterium pullorum subsp. saeculare*]; Proline--tRNA ligase - *Bifidobacterium longum subsp. infantis*; Proline--tRNA ligase - *Bifidobacterium longum*; Proline--tRNA ligase - *Bifidobacterium angulatum*; Proline--tRNA ligase - *Bifidobacterium catenulatum*; Proline--tRNA ligase - *Bifidobacterium dentium*; Proline--tRNA ligase - *Bifidobacterium catenulatum*; Proline--tRNA ligase - *Bifidobacterium longum subsp. infantis*; Proline--tRNA ligase - *Bifidobacterium bifidum*
tr.WP_173755788.1.	carboxyl transferase [Clostridiales]; Methylmalonyl-CoA carboxyltransferase 12S subunit - *Blautia luti*; carboxyl transferase domain-containing protein [*Blautia schinkii*]; carboxyl transferase domain protein - *Blautia obeum*
tr.A0A1V8PQF9.	Transketolase - *Bifidobacterium catenulatum*

Table S23C. clinical data 6M

set 1	set 2	set 3	set 4	set 5	mean
SCORADMAN	SCORADMAN	SCORADMAN	SCORADMAN	SCORADMAN	SCORADMAN
alrgyfat	alrgyfat	alrgyfat	alrgyfat	alrgyfat	alrgyfat
alrgymot	SPTOWF	alrgymot	alrgymot	alrgymot	alrgymot
SPTOWF	FOODTRIG4	SPTOWF	SPTOWF	SPTOWF	SPTOWF
STOOLCONSIST	SPTOP	STOOLCONSIST	STOOLCONSIST	num_ab	num_ab
STOOLCOLOUR		STOOLCOLOUR	STOOLCOLOUR	STOOLCOLOUR	STOOLCONSIST
STOOLFREQ		STOOLFREQ	STOOLFREQ	STOOLFREQ	STOOLCOLOUR
treatment		treatment	FOODTRIG4	treatment	STOOLFREQ
FOODTRIG4		delivery	age	FOODTRIG4	treatment
delivery		age	num_inf	delivery	FOODTRIG4
age		SPTOSB	SPTOSB	age	delivery
num_inf		sibl	GASWIND	num_inf	age
SPTOP				SPTOSB	num_inf
GASWIND				SPTOP	SPTOSB
SPITTING				GASWIND	SPTOP
sex				FOODTRIG7	GASWIND
FOODTRIG2					SPITTING

(yellow)	variable importance >= 0.01 for all 5 models
(light green)	variable importance >= 0.01 for 4 models

Explanation variables: see Table S2

Table S23. (continued)

<u>Table S23D.</u> immune data 6M

The abbreviations in the first table below are explained in the next table

set 1	set 2	set 3	set 4	set 5	mean
X4E.BP1	MCP.1	X4E.BP1	X4E.BP1	X4E.BP1	X4E.BP1
IL.1.alpha	LAP.TGF.beta.1	IL.1.alpha	IL.1.alpha	IL.1.alpha	IL.1.alpha
CXCL5	TRAIL	CXCL5	CXCL5	CXCL5	CXCL5
CCL4	TNFRSF9	CCL4	CCL4	CCL4	CCL4
IL.12B	CSF.1	IL.15RA	TGF.alpha	MCP.1	MCP.1
TGF.alpha	MMP.1	EN.RAGE	IL.15RA	IL.12B	IL.12B
IL.15RA	OSM	MCP.4	EN.RAGE	TGF.alpha	TGF.alpha
LAP.TGF.beta.1	Flt3L	IL7	OPG	PD.L1	PD.L1
STAMBP	CD5	MMP.10	CCL3	LAP.TGF.beta.1	IL.15RA
CASP.8	IL.18R1	LIF	MCP.4	STAMBP	LAP.TGF.beta.1
TNFRSF9	CX3CL1		CCL28	EN.RAGE	STAMBP
OPG	DNER		IL7	CASP.8	EN.RAGE
MCP.4	IL8		IL.10RB	TRAIL	CASP.8
MMP.1			CCL19	TNFRSF9	TRAIL
TNF			CXCL10	CSF.1	TNFRSF9
CCL28			MMP.10	OPG	CSF.1
Flt3L				LIF.R	OPG
IL.10RB				CCL3	LIF.R
CXCL10				MMP.1	CCL3
ADA				FGF.19	MCP.4
CCL23				TNF	MMP.1
CD5				VEGFA	FGF.19
IL.18R1				IL7	TNF
MCP.2				OSM	VEGFA
				Flt3L	CCL28
				ADA	IL7
				SIRT2	OSM
				CCL23	Flt3L
				TWEAK	IL.10RB
				IL.18R1	CCL19
				uPA	
				CX3CL1	
				DNER	
				CCL20	
				TNFSF14	
				IL6	
				CD40	
				CXCL6	

Table S23. (continued)

Table S23D. (continued)

set 1	set 2	set 3	set 4	set 5	mean
				CXCL11	
				HGF	
				IL18	
				CDCP1	
				IL8	
				SCF	
				CXCL9	

variable importance >= 0.01 for 4 models

Abbreviations

variable	uniprot ID	name
X4E.BP1	Q13541	Eukaryotic translation initiation factor 4E-binding protein 1 (4E-BP1)
IL.1.alpha	P01583	Interleukin-1 alpha (IL-1 alpha)
CXCL5	P42830	C-X-C motif chemokine 5 (CXCL5)
CCL4	P13236	C-C motif chemokine 4 (CCL4)
IL.12B	P29460	Interleukin-12 subunit beta (IL-12B)
TGF.alpha	P01135	Transforming growth factor alpha (TGF-alpha)
IL.15RA	Q13261	Interleukin-15 receptor subunit alpha (IL-15RA)
LAP.TGF.beta.1	P01137	Latency-associated peptide transforming growth factor beta-1 (LAP TGF-beta-1)
STAMBP	O95630	STAM-binding protein (STAMBP)
CASP.8	Q14790	Caspase-8 (CASP-8)
TNFRSF9	Q07011	Tumor necrosis factor receptor superfamily member 9 (TNFRSF9)
OPG	O00300	Osteoprotegerin (OPG)
MCP.4	Q99616	Monocyte chemotactic protein 4 (MCP-4)
MMP.1	P03956	Matrix metalloproteinase-1 (MMP-1)
TNF	P01375	Tumor necrosis factor (TNF)
CCL28	Q9NRJ3	C-C motif chemokine 28 (CCL28)
Flt3L	P49771	Fms-related tyrosine kinase 3 ligand (Flt3L)
IL.10RB	Q08334	Interleukin-10 receptor subunit beta (IL-10RB)
CXCL10	P02778	C-X-C motif chemokine 10 (CXCL10)
ADA	P00813	Adenosine Deaminase (ADA)
CCL23	P55773	C-C motif chemokine 23 (CCL23)
CD5	P06127	T-cell surface glycoprotein CD5 (CD5)
IL.18R1	Q13478	Interleukin-18 receptor 1 (IL-18R1)
MCP.2	P80075	Monocyte chemotactic protein 2 (MCP-2)
MCP.1	P13500	Monocyte chemotactic protein 1 (MCP-1)
TRAIL	P50591	TNF-related apoptosis-inducing ligand (TRAIL)

Table S23. (continued)

Table S23D. (continued)

variable	uniprot ID	name
CSF.1	P09603	Macrophage colony-stimulating factor 1 (CSF-1)
OSM	P13725	Oncostatin-M (OSM)
CX3CL1	P78423	Fractalkine (CX3CL1)
DNER	Q8NFT8	Delta and Notch-like epidermal growth factor-related receptor (DNER)
IL8	P10145	Interleukin-8 (IL-8)
EN.RAGE	P80511	Protein S100-A12 (EN-RAGE)
IL7	P13232	Interleukin-7 (IL-7)
MMP.10	P09238	Matrix metalloproteinase-10 (MMP-10)
LIF	P15018	Leukemia inhibitory factor (LIF)
CCL3	P10147	C-C motif chemokine 3 (CCL3)
CCL19	Q99731	C-C motif chemokine 19 (CCL19)
PD.L1	Q9NZQ7	Programmed cell death 1 ligand 1 (PD-L1)
LIF.R	P42702	Leukemia inhibitory factor receptor (LIF-R)
FGF.19	O95750	Fibroblast growth factor 19 (FGF-19)
VEGFA	P15692	Vascular endothelial growth factor A (VEGF-A)
SIRT2	Q8IXJ6	SIR2-like protein 2 (SIRT2)
TWEAK	O43508	Tumor necrosis factor (Ligand) superfamily, member 12 (TWEAK)
uPA	P00749	Urokinase-type plasminogen activator (uPA)
CCL20	P78556	C-C motif chemokine 20 (CCL20)
TNFSF14	O43557	Tumor necrosis factor ligand superfamily member 14 (TNFSF14)
IL6	P05231	Interleukin-6 (IL6)
CD40	P25942	CD40L receptor (CD40)
CXCL6	P80162	C-X-C motif chemokine 6 (CXCL6)
CXCL11	O14625	C-X-C motif chemokine 11 (CXCL11)
HGF	P14210	Hepatocyte growth factor (HGF)
IL18	Q14116	Interleukin-18 (IL-18)
CDCP1	Q9H5V8	CUB domain-containing protein 1 (CDCP1)
SCF	P21583	Stem cell factor (SCF)
CXCL9	Q07325	C-X-C motif chemokine 9 (CXCL9)

Table S23. (continued)

<u>Table S23E.</u> metabolomics – platform for polar and semi-polar metabolites – negative mode 6M

set 1	set 2	set 3	set 4	set 5	mean
myo-Inositol/ Galactose/ Fructose	myo-Inositol/ Galactose/ Fructose	myo-Inositol/ Galactose/ Fructose	myo-Inositol/ Galactose/ Fructose	myo-Inositol/ Galactose/ Fructose	myo-Inositol/ Galactose/ Fructose
Protocatechuic acid	Protocatechuic acid	Mandelic acid	Protocatechuic acid	Protocatechuic acid	Protocatechuic acid
Pyrocatechol	Pyrocatechol	p-Hydroxyphenylacetic acid	Pyrocatechol	Pyrocatechol	Pyrocatechol
Phenylacetic acid	Phenylacetic acid	Pyruvic acid	Phenylacetic acid	Phenylacetic acid	Phenylacetic acid
3-Hydroxybutyric acid	3-Hydroxybutyric acid	O-Acetylserine/ Glutamic acid	3-Hydroxybutyric acid	3-Hydroxybutyric acid	3-Hydroxybutyric acid
N6-Carboxymethyllysine	N6-Carboxymethyllysine		N6-Carboxymethyllysine	N6-Carboxymethyllysine	N6-Carboxymethyllysine
	Histidine		Histidine	Histidine	Histidine
	Syringic acid		Syringic acid	Syringic acid	Syringic acid
	Argininosuccinic acid		trans-Aconitic acid	trans-Aconitic acid	trans-Aconitic acid
			Phenylacetylglutamine	Phenylacetylglutamine	Phenylacetylglutamine
			N-Acetylneuraminic acid	N-Acetylneuraminic acid	N-Acetylneuraminic acid
			2,5-Furandicarboxylic acid	2,5-Furandicarboxylic acid	2,5-Furandicarboxylic acid
			FAD	N-Acetylglutamine	FAD
			Pseudouridine	Gluconic acid	N-Acetylglutamine
			Xylulose	2-Hydroxyethanesulfonate	Gluconic acid
			Thymidine	Pseudouridine	2-Hydroxyethanesulfonate
			Lysine	Xylulose	Pseudouridine
			3-Methylxanthine/1-Methylxanthine/ 7-Methylxanthine	Thymidine	Xylulose
			4-Hydroxycinnamic acid	Lysine	
			Citric acid	3-Methylxanthine/1-Methylxanthine/ 7-Methylxanthine	
			Hippuric acid	4-Hydroxycinnamic acid	
			Hydrocinnamic acid	Taurine	
			4-Hydroxybenzoic acid	Citric acid	
			Uridine	Mandelic acid	
			Indoxyl glucoside	p-Hydroxyphenylacetic acid	
			Oxoglutaric acid	Hydrocinnamic acid	
			Glycine	4-Hydroxybenzoic acid	
			Dihydrocaffeic acid/HPHPA/Hydroxyphenyllactic acid	Uridine	

Table S23. (continued)

Table S23E. (continued)

**set 1**	**set 2**	**set 3**	**set 4**	**set 5**	**mean**
			Deoxyinosine	Argininosuccinic acid	
			Tryptophan	Oxoglutaric acid	
			Pyroglutamic acid	Indolelactic acid	
			Methionine.sulfoxide	Dihydrocaffeic acid/HPHPA/ Hydroxyphenyllactic acid	
			p-Cresol	Deoxyinosine	
			3-Hydroxybenzoic acid	Tryptophan	
			N-Acetyltryptophan	Methionine.sulfoxide	
			Ferulic acid	Xanthine	
			Pregnenolone sulfate	N2-gamma-Glutamylglutamine	
			N-Acetylserine	3-Methyl-2-oxovaleric acid	
			Phenyllactic acid	N-alpha-Acetylarginine	
			Ascorbic acid		
			Quinolinic acid		
	variable importance >= 0.01 for all 5 models				
	variable importance >= 0.01 for 4 models				

**Compound_name_reported**	**Compound_name_HMDB**
myo-Inositol/ Galactose/ Fructose	myo-Inositol/ D-Galactose/ D-Fructose
Protocatechuic acid	Protocatechuic acid
Pyrocatechol	Pyrocatechol
Phenylacetic acid	Phenylacetic acid
3-Hydroxybutyric acid	3-Hydroxybutyric acid
N6-Carboxymethyllysine	N6-Carboxymethyllysine
Histidine	L-Histidine
Syringic acid	Syringic acid
Argininosuccinic acid	Argininosuccinic acid
Mandelic acid	Mandelic acid
p-Hydroxyphenylacetic acid	p-Hydroxyphenylacetic acid
Pyruvic acid	Pyruvic acid
O-Acetylserine/ Glutamic acid	O-Acetylserine/ Glutamic acid
trans-Aconitic acid	trans-Aconitic acid
Phenylacetylglutamine	Phenylacetylglutamine
N-Acetylneuraminic acid	N-Acetylneuraminic acid
2,5-Furandicarboxylic acid	2,5-Furandicarboxylic acid
FAD	FAD
Pseudouridine	Pseudouridine
Xylulose	D-Xylulose

Table S23. (continued)

Table S23E. (continued)

Compound_name_reported	Compound_name_HMDB
Thymidine	Thymidine
Lysine	L-Lysine
3-Methylxanthine/1-Methylxanthine/ 7-Methylxanthine	3-Methylxanthine/1-Methylxanthine/7-Methylxanthine
4-Hydroxycinnamic acid	4-Hydroxycinnamic acid
Citric acid	Citric acid
Hippuric acid	Hippuric acid
Hydrocinnamic acid	Hydrocinnamic acid
4-Hydroxybenzoic acid	4-Hydroxybenzoic acid
Uridine	Uridine
Indoxyl glucoside	Indoxyl glucoside
Oxoglutaric acid	Oxoglutaric acid
Glycine	Glycine
Dihydrocaffeic acid/HPHPA/Hydroxyphenyllactic acid	3,4-Dihydroxyhydrocinnamic acid/HPHPA/ Hydroxyphenyllactic acid
Deoxyinosine	Deoxyinosine
Tryptophan	L-Tryptophan
Pyroglutamic acid	Pyroglutamic acid
Methionine.sulfoxide	L-Methionine.sulfoxide
p-Cresol	p-Cresol
3-Hydroxybenzoic acid	3-Hydroxybenzoic acid
N-Acetyltryptophan	N-Acetyltryptophan
Ferulic acid	Ferulic acid
Pregnenolone sulfate	Pregnenolone sulfate
N-Acetylserine	N-Acetylserine
Phenyllactic acid	Phenyllactic acid
Ascorbic acid	Ascorbic acid
Quinolinic acid	Quinolinic acid
N-Acetylglutamine	N-Acetylglutamine
Gluconic acid	Gluconic acid
2-Hydroxyethanesulfonate	2-Hydroxyethanesulfonate
Taurine	Taurine
Indolelactic acid	Indolelactic acid
Xanthine	Xanthine
N2-gamma-Glutamylglutamine	N2-gamma-Glutamylglutamine
3-Methyl-2-oxovaleric acid	3-Methyl-2-oxovaleric acid
N-alpha-Acetylarginine	N-a-Acetyl-L-arginine

Table S23. (continued)

Table S23F. metabolomics – platform for polar and semi-polar metabolites – positive mode 6M

set 1	set 2	set 3	set 4	set 5	mean
N1-Methyl-4-pyridone-3-carboxamide/Nudiflоramide	N1-Methyl-4-pyridone-3-carboxamide/Nudifloramide	N1-Methyl-4-pyridone-3-carboxamide/Nudifloramide	N1-Methyl-4-pyridone-3-carboxamide/Nudifloramide	N1-Methyl-4-pyridone-3-carboxamide/Nudifloramide	N1-Methyl-4-pyridone-3-carboxamide/Nudifloramide
Citrulline	Citrulline	Citrulline	Citrulline	Citrulline	Citrulline
Dodecanoylcarnitine	Dodecanoylcarnitine	Dodecanoylcarnitine	Dihydrouracil	Dodecanoylcarnitine	Dodecanoylcarnitine
Dihydrouracil	Dihydrouracil	Dihydrouracil	5-Hydroxytryptophan	Dihydrouracil	Dihydrouracil
N6,N6,N6-Trimethyllysine	N6,N6,N6-Trimethyllysine	N6,N6,N6-Trimethyllysine	Feature_mz_130.086	N6,N6,N6-Trimethyllysine	N6,N6,N6-Trimethyllysine
Betaine	Guanidoacetic acid	Guanidoacetic acid	Serotonin	Guanidoacetic acid	Guanidoacetic acid
5-Hydroxytryptophan	Betaine	Betaine	Riboflavin	Betaine	Betaine
Feature_mz_130.086	Feature_mz_130.086	Feature_mz_130.086	Threonine/Homoserine	5-Hydroxytryptophan	5-Hydroxytryptophan
Riboflavin	Serotonin	Beta-Guanidinopropionic acid		Feature_mz_130.086	Feature_mz_130.086
Picolinic acid	Pyridoxal	Choline		Serotonin	Serotonin
Beta-Guanidinopropionic acid	Aspartic acid	Creatine		Riboflavin	Riboflavin
Uracil	Beta-Guanidinopropionic acid			Pyridoxal	Pyridoxal
N-Acetyltyrosine	5-Aminopentanoic acid			Picolinic acid	Picolinic acid
5-Aminolevulinic acid/4-Hydroxyproline	Uracil			Aspartic acid	Aspartic acid
Tyrosine	N-Acetyltyrosine			5-Aminopentanoic acid	Beta-Guanidinopropionic acid
4-Guanidinobutanoic acid	1-Methyladenosine/N6-Methyladenosine/2'-O-Methyladenosine			Uracil	5-Aminopentanoic acid
	Kynurenic acid			Choline	Uracil
	Tyramine			1-Methyladenosine/N6-Methyladenosine/2'-O-Methyladenosine	N-Acetyltyrosine
	Tryptamine			Kynurenic acid	
	Creatine			Tyramine	
	Glycylproline			Tryptamine	
	Threonine/Homoserine			Glycylproline	
	Methionine			5-Aminolevulinic acid/4-Hydroxyproline	
	Adenosine			Tyrosine	
	Phenylalanine			Methionine	
	Ornithine			Phenylethylamine	
	Leucine			Carnitine	

Table S23. (continued)

Table S23F. (continued)

set 1	set 2	set 3	set 4	set 5	mean
	beta-Alanine/Alanine/ Sarcosine			Cytidine	
	Nicotinic acid			Symmetric dimethylarginine/Asymmetric dimethylarginine	
				Proline	
				Xanthurenic acid	
				Spermidine	
				Biotin	
				Inosine	
				Sphinganine	

(yellow)	variable importance >= 0.01 for all 5 models
(light green)	variable importance >= 0.01 for 4 models

Compound_name_reported	Compound_name_HMDB
N1-Methyl-4-pyridone-3-carboxamide/Nudifloramide	N1-Methyl-4-pyridone-3-carboxamide/Nudifloramide
Citrulline	Citrulline
Dodecanoylcarnitine	Dodecanoylcarnitine
Dihydrouracil	Dihydrouracil
N6,N6,N6-Trimethyllysine	N6,N6,N6-Trimethyl-L-lysine
Betaine	Betaine
5-Hydroxytryptophan	5-Hydroxy-L-tryptophan
Feature_mz_130.086	unknown
Riboflavin	Riboflavin
Picolinic acid	Picolinic acid
Beta-Guanidinopropionic acid	Beta-Guanidinopropionic acid
Uracil	Uracil
N-Acetyltyrosine	N-Acetyl-L-tyrosine
5-Aminolevulinic acid/4-Hydroxyproline	5-Aminolevulinic acid/4-Hydroxyproline
Tyrosine	L-Tyrosine
4-Guanidinobutanoic acid	4-Guanidinobutanoic acid
Guanidoacetic acid	Guanidoacetic acid
Serotonin	Serotonin
Pyridoxal	Pyridoxal
Aspartic acid	L-Aspartic acid
5-Aminopentanoic acid	5-Aminopentanoic acid
1-Methyladenosine/N6-Methyladenosine/2'-O-Methyladenosine	1-Methyladenosine/N6-Methyladenosine/2'-O-Methyladenosine
Kynurenic acid	Kynurenic acid

Table S23. (continued)

Table S23F. (continued)

**Compound_name_reported**	**Compound_name_HMDB**
Tyramine	Tyramine
Tryptamine	Tryptamine
Creatine	Creatine
Glycylproline	Glycylproline
Threonine/Homoserine	L-Threonine/ L-Homoserine
Methionine	L-Methionine
Adenosine	Adenosine
Phenylalanine	L-Phenylalanine
Ornithine	Ornithine
Leucine	L-Leucine
beta-Alanine/Alanine/Sarcosine	beta-Alanine/L-Alanine/Sarcosine
Nicotinic acid	Nicotinic acid
Choline	Choline
Phenylethylamine	Phenylethylamine
Carnitine	L-Carnitine
Cytidine	Cytidine
Symmetric dimethylarginine/Asymmetric dimethylarginine	Symmetric dimethylarginine
Proline	L-Proline
Xanthurenic acid	Xanthurenic acid
Spermidine	Spermidine
Biotin	Biotin
Inosine	Inosine
Sphinganine	Sphinganine

Table S23. (continued)

Table S23G. metabolomics – platform for polar and semi-polar metabolites – positive mode 12M

set 1	set 2	set 3	set 4	set 5	mean
Feature_mz_130.086	Feature_mz_130.086	Feature_mz_130.086	Feature_mz_130.086	Feature_mz_130.086	Feature_mz_130.086
Citrulline	Citrulline	Citrulline	Citrulline	Citrulline	Citrulline
Targinine/Homoarginine	Targinine/Homoarginine	Targinine/Homoarginine	Ethanolamine	Targinine/Homoarginine	Targinine/Homoarginine
Ornithine	Ornithine	Ornithine	Sphinganine	Ornithine	Ornithine
Threonine/Homoserine	Threonine/Homoserine	Threonine/Homoserine		Threonine/Homoserine	Threonine/Homoserine
Thymine	Thymine	Thymine		Thymine	Thymine
1-Methyladenosine/N6-Methyladenosine/2'-O-Methyladenosine	Ethanolamine	1-Methyladenosine/N6-Methyladenosine/2'-O-Methyladenosine		Cadaverine	1-Methyladenosine/N6-Methyladenosine/2'-O-Methyladenosine
Serotonin	Cadaverine	Ethanolamine		Sphinganine	Ethanolamine
Sphinganine	Serotonin	Cadaverine		Pyridoxal	Cadaverine
Deoxyguanosine	Pyridoxal	Serotonin		Arginine	Serotonin
5-Aminolevulinic acid/4-Hydroxyproline	Deoxyguanosine	Sphinganine			Sphinganine
Cytidine	5-Hydroxytryptophan	Pyridoxal			Pyridoxal
Thiamine	5-Aminolevulinic acid/4-Hydroxyproline	Deoxyguanosine			Deoxyguanosine
N6,N6,N6-Trimethyllysine	N2,N2-Dimethylguanosine	5-Hydroxytryptophan			5-Hydroxytryptophan
Aspartic acid	Thiamine	5-Aminolevulinic acid/4-Hydroxyproline			5-Aminolevulinic acid/4-Hydroxyproline
Picolinic acid	Guanidoacetic acid	N2,N2-Dimethylguanosine			N2,N2-Dimethylguanosine
Carnitine	Tyrosine	Cytidine			Cytidine
	Betaine	Thiamine			Thiamine
	Indoleacetic acid	Arginine			
	beta-Alanine/Alanine/Sarcosine	N6,N6,N6-Trimethyllysine			
	Choline	Dihydrouracil			
	N-Acetylcadaverine	Guanidoacetic acid			
	Adenine	Picolinic acid			
		Betaine			
		Cytosine			
		Quinaldic acid			
		Xanthurenic acid			
		Indoleacetic acid			
		Proline			
		Adenosine			
		N1-Methyl-4-pyridone-3-carboxamide/Nudiflor amide			
		Pipecolic acid			
		Kynurenic acid			

variable importance >= 0.01 for all 5 models

variable importance >= 0.01 for 4 models

Table S23. (continued)

Table S23G. (continued)

**Compound_name_reported**	**Compound_name_HMDB**
Feature_mz_130.086	unknown
Citrulline	Citrulline
Targinine/Homoarginine	L-Targinine/Homo-L-arginine
Ornithine	Ornithine
Threonine/Homoserine	L-Threonine/ L-Homoserine
Thymine	Thymine
1-Methyladenosine/N6-Methyladenosine/2'-O-Methyladenosine	1-Methyladenosine/N6-Methyladenosine/2'-O-Methyladenosine
Serotonin	Serotonin
Sphinganine	Sphinganine
Deoxyguanosine	Deoxyguanosine
5-Aminolevulinic acid/4-Hydroxyproline	5-Aminolevulinic acid/4-Hydroxyproline
Cytidine	Cytidine
Thiamine	Thiamine
N6,N6,N6-Trimethyllysine	N6,N6,N6-Trimethyl-L-lysine
Aspartic acid	L-Aspartic acid
Picolinic acid	Picolinic acid
Carnitine	L-Carnitine
Ethanolamine	Ethanolamine
Cadaverine	Cadaverine
Pyridoxal	Pyridoxal
5-Hydroxytryptophan	5-Hydroxy-L-tryptophan
N2,N2-Dimethylguanosine	N2,N2-Dimethylguanosine
Guanidoacetic acid	Guanidoacetic acid
Tyrosine	L-Tyrosine
Betaine	Betaine
Indoleacetic acid	Indoleacetic acid
beta-Alanine/Alanine/Sarcosine	beta-Alanine/L-Alanine/Sarcosine
Choline	Choline
N-Acetylcadaverine	N-Acetylcadaverine
Adenine	Adenine
Arginine	L-Arginine
Dihydrouracil	Dihydrouracil
Cytosine	Cytosine
Quinaldic acid	Quinaldic acid
Xanthurenic acid	Xanthurenic acid
Proline	L-Proline
Adenosine	Adenosine
N1-Methyl-4-pyridone-3-carboxamide/Nudifloramide	N1-Methyl-4-pyridone-3-carboxamide/Nudifloramide
Pipecolic acid	Pipecolic acid
Kynurenic acid	Kynurenic acid

Table S24. Occurrence of metabolites with permutation-based variable importance ≥ 0.01 (at 6 months or 12 months) in genera with permutation-based variable importance ≥ 0.01 (at baseline). Abbreviations: MPN: metabolomics – platform for polar and semi-polar metabolites – negative mode; MPP: metabolomics – platform for polar and semi-polar metabolites – positive mode; VMH: Vitual Metabolic Human.

**platform**	**Compound name HMDB**	**Compound name reported**	**HMDB ID**	**VMH ID**	**visit(s)**	**occurs in …**
MPN	myo-Inositol/D-Galactose/<br>D-Fructose	myo-Inositol/Galactose/<br>Fructose	HMDB0000211	INOST	6M	*Klebsiella:* present (VMH)<br>*Haemophilus:* present (VMH)<br>*Hungatella*: present (VMH)<br>*Lachnoclostridium:* present (VMH)<br>*Bacteroides:* present (VMH)<br>*Clostridium_sensu_stricto_1:* present (VMH)<br>*Lachnospiraceae_unclassified*: present (VMH)<br>*Streptococcus:* present (VMH)<br>*Erysipelatoclostridium*: present (VMH)
MPN	Protocatechuic acid	Protocatechuic acid	HMDB0001856	34DHB	6M	*Klebsiella:* present (VMH)
MPN	Pyrocatechol	Pyrocatechol	HMDB0000957	CATECHOL	6M	*Klebsiella:* present (VMH)<br>*Clostridium_sensu_stricto_1:* present (VMH)
MPN	Phenylacetic acid	Phenylacetic acid	HMDB0000209	PAC	6M	*Klebsiella:* present (VMH), carbon source (VMH)<br>*Dialister :* present (VMH)<br>*Lachnoclostridium:* present (VMH)<br>*Bacteroides :* present (VMH), fermentation product (VMH)<br>*Clostridium_sensu_stricto_1 :* present (VMH)<br>*Lachnospiraceae_unclassified :* present (VMH)<br>*Streptococcus :* present (VMH)<br>*Erysipelatoclostridium :* present (VMH)
MPN	3-Hydroxybutyric acid	3-Hydroxybutyric acid	HMDB0000357	BHB	6M	*Klebsiella :* present (VMH)<br>*Lachnoclostridium :* present (VMH)<br>*Bacteroides :* present (VMH)<br>*Clostridium_sensu_stricto_1 :* present (VMH)<br>*Lachnospiraceae_unclassified:* present (VMH)<br>*Streptococcus:* present (VMH)
MPN	N6-Carboxymethyllysine	N6-Carboxymethyllysine	HMDB0240347	Not Available	6M	not reported to occur in any genera with variable importance ≥ 0.01

Table S24. (continued)

platform	Compound name HMDB	Compound name reported	HMDB ID	VMH ID	visit(s)	occurs in …
MPN	L-Histidine	Histidine	HMDB0000177	HIS_L	6M	*Klebsiella:* present (VMH + HMDB), carbon source (VMH)<br>*Haemophilus :* present (VMH + HMDB)<br>*Gemella* : present (VMH + HMDB)<br>*Dialister* : present (VMH + HMDB)<br>*Hungatella :* present (VMH + HMDB)<br>*Lachnoclostridium* : present (VMH + HMDB)<br>*Bacteroides* : present (VMH + HMDB)<br>*Clostridium_sensu_stricto_1 :* present (VMH + HMDB)<br>*Lachnospiraceae_unclassified* : present (VMH + HMDB)<br>*TM7x* (not in VMH) : present (HMDB)<br>*Streptococcus:* present (VMH + HMDB)<br>*Collinsella*: present (VMH + HMDB)<br>*Erysipelatoclostridium:* present (VMH + HMDB)<br>*Robinsoniella* (not in VMH): present (HMDB)<br>Remark: HMDB: present in all organisms, including bacteria.
MPN	Syringic acid	Syringic acid	HMDB0002085	Not Available	6M	not reported to occur in any genera with variable importance ≥ 0.01
MPN	trans-Aconitic acid	trans-Aconitic acid	HMDB0000958	Not Available	6M	not reported to occur in any genera with variable importance ≥ 0.01
MPN	Phenylacetylglutamine	Phenylacetylglutamine	HMDB0006344	PHEACGLN	6M	*Lachnospiraceae_unclassified*: present (HMDB)<br>Remark: HMDB: *Lachnospiraceae*<br>(PMID: 26241311: *Lachnospiraceae unclassified*. *Lachnoclostridium* and *Robinsoniella* not mentioned).

Table S24. (continued)

platform	Compound name HMDB	Compound name reported	HMDB ID	VMH ID	visit(s)	occurs in …
MPN	N-Acetylneuraminic acid	N-Acetylneuraminic acid	HMDB0000230	ACNAM	6M	*Klebsiella:* present (VMH)<br>*Haemophilus:* present (VMH), carbon source (VMH)<br>*Gemella* : present (VMH)<br>*Hungatella :* present (VMH)<br>*Lachnoclostridium* : present (VMH), carbon source (VMH)<br>*Bacteroides :* present (VMH), carbon source (VMH)<br>*Clostridium_sensu_stricto_1 :* present (VMH), carbon source (VMH)<br>*Lachnospiraceae_unclassified :* present (VMH)<br>*Streptococcus* : present (VMH), carbon source (VMH)<br>*Collinsella :* present (VMH), carbon source (VMH)<br>*Erysipelatoclostridium* : present (VMH)
MPN	2,5-Furandicarboxylic acid	2,5-Furandicarboxylic acid	HMDB0004812	Not Available	6M	not reported to occur in any genera with variable importance ≥ 0.01
MPN	FAD	FAD	HMDB0001248	FAD	6M	*Klebsiella:* present (VMH + HMDB)<br>*Haemophilus:* present (VMH + HMDB)<br>*Gemella:* present (VMH + HMDB)<br>*Dialister:* present (VMH + HMDB)<br>*Hungatella*: present (VMH + HMDB)<br>*Lachnoclostridium:* present (VMH + HMDB)<br>*Bacteroides*: present (VMH + HMDB)<br>*Clostridium_sensu_stricto_1:* present (VMH + HMDB)<br>*Lachnospiraceae_unclassified:* present (VMH + HMDB)<br>*TM7x* (not in VMH): present (HMDB)<br>*Streptococcus*: present (VMH + HMDB)<br>*Collinsella:* present (VMH + HMDB)<br>*Erysipelatoclostridium*: present (VMH + HMDB)<br>*Robinsoniella* (not in VMH): present (HMDB)<br>Remark: HMDB: present in all organisms, including bacteria.
MPN	N-Acetylglutamine	N-Acetylglutamine	HMDB0006029	Not Available	6M	not reported to occur in any genera with variable importance ≥ 0.01

Table S24. (continued)

platform	Compound name HMDB	Compound name reported	HMDB ID	VMH ID	visit(s)	occurs in …
MPN	Gluconic acid	Gluconic acid	HMDB0000625	GLCN	6M	*Klebsiella*: present (VMH + HMDB), carbon source (VMH)<br>*Haemophilus :* present (HMDB)<br>*Gemella :* present (VMH + HMDB)<br>*Dialister :* present (HMDB)<br>*Hungatella :* present (VMH + HMDB)<br>*Lachnoclostridium :* present (VMH + HMDB)<br>*Bacteroides :* present (VMH + HMDB)<br>*Clostridium_sensu_stricto_1 :* present (VMH + HMDB)<br>*Lachnospiraceae_unclassified :* present (VMH + HMDB)<br>*TM7x* (not in VMH) : present (HMDB)<br>*Streptococcus:* present (VMH + HMDB)<br>*Collinsella:* present (HMDB)<br>*Erysipelatoclostridium:* present (HMDB)<br>*Robinsoniella* (not in VMH): present (HMDB)<br>Remark: HMDB: present in all organisms, including bacteria.
MPN	2-Hydroxyethanesulfonate	2-Hydroxyethanesulfonate	HMDB0003903	ISETAC	6M	*Klebsiella :* present (VMH)<br>*Dialister :* present (VMH)<br>*Hungatella :* present (VMH)<br>*Lachnoclostridium :* present (VMH)<br>*Clostridium_sensu_stricto_1 :* present (VMH)<br>*Streptococcus :* present (VMH)<br>*Collinsella :* present (VMH)
MPN	Pseudouridine	Pseudouridine	HMDB0000767	Not Available	6M	*Klebsiella*: present (HMDB)<br>*Haemophilus:* present (HMDB)<br>*Gemella:* present (HMDB)<br>*Dialister:* present (HMDB)<br>*Hungatella:* present (HMDB)<br>*Lachnoclostridium:* present (HMDB)<br>*Bacteroides:* present (HMDB)<br>*Clostridium_sensu_stricto_1:* present (HMDB)<br>*Lachnospiraceae_unclassified:* present (HMDB)<br>*TM7x* (not in VMH): present (HMDB)<br>*Streptococcus:* present (HMDB)<br>*Collinsella:* present (HMDB)<br>*Erysipelatoclostridium:* present (HMDB)<br>*Robinsoniella* (not in VMH): present (HMDB)<br>Remark: HMDB: present in all organisms, including bacteria.

Table S24. (continued)

platform	Compound name HMDB	Compound name reported	HMDB ID	VMH ID	visit(s)	occurs in …
MPN	D-Xylulose	Xylulose	HMDB0001644	XYLU_D	6M	*Klebsiella*: present (VMH)<br>*Haemophilus*: present (VMH)<br>*Gemella*: present (VMH)<br>*Dialister*: present (VMH)<br>*Hungatella*: present (VMH)<br>*Lachnoclostridium*: present (VMH)<br>*Bacteroides*: present (VMH)<br>*Clostridium_sensu_stricto_1*: present (VMH)<br>*Lachnospiraceae_unclassified*: present (VMH)<br>*Streptococcus*: present (VMH)<br>*Collinsella*: present (VMH)<br>*Erysipelatoclostridium*: present (VMH)
MPP	N1-Methyl-4-pyridone-3-carboxamide/ Nudifloramide	N1-Methyl-4-pyridone-3-carboxamide/ Nudifloramide	HMDB0004194	M02506	6M	not reported to occur in any genera with variable importance ≥ 0.01
MPP	Citrulline	Citrulline	HMDB0000904	CITR_L	6M, 12M	*Klebsiella*: present (VMH + HMDB)<br>*Haemophilus*: present (VMH + HMDB)<br>*Gemella*: present (VMH + HMDB)<br>*Dialister*: present (VMH + HMDB)<br>*Hungatella:* present (VMH + HMDB)<br>*Lachnoclostridium*: present (VMH + HMDB)<br>*Bacteroides:* present (VMH + HMDB)<br>*Clostridium_sensu_stricto_1*: present (VMH + HMDB)<br>*Lachnospiraceae_unclassified*: present (VMH + HMDB)<br>*TM7x* (not in VMH): present (HMDB)<br>*Streptococcus:* present (VMH + HMDB)<br>*Collinsella*: present (VMH + HMDB)<br>*Erysipelatoclostridium:* present (VMH + HMDB)<br>*Robinsoniella* (not in VMH): present (HMDB)<br>Remark: HMDB: present in all organisms, including bacteria.
MPP	Dodecanoylcarnitine	Dodecanoylcarnitine	HMDB0002250	DDECCRN	6M	not reported to occur in any genera with variable importance ≥ 0.01

Table S24. (continued)

platform	Compound name HMDB	Compound name reported	HMDB ID	VMH ID	visit(s)	occurs in …
MPP	Dihydrouracil	Dihydrouracil	HMDB0000076	56DURA	6M	*Klebsiella*: present (VMH + HMDB)<br>*Haemophilus*: present (VMH + HMDB)<br>*Gemella*: present (VMH + HMDB)<br>*Dialister*: present (VMH + HMDB)<br>*Hungatella*: present (VMH + HMDB)<br>*Lachnoclostridium*: present (VMH + HMDB)<br>*Bacteroides*: present (VMH + HMDB)<br>*Clostridium_sensu_stricto_1*: present (VMH + HMDB)<br>*Lachnospiraceae_unclassified*: present (VMH + HMDB)<br>*TM7x* (not in VMH): present (HMDB)<br>*Streptococcus*: present (VMH + HMDB)<br>*Collinsella*: present (VMH + HMDB)<br>*Erysipelatoclostridium*: present (VMH + HMDB)<br>*Robinsoniella* (not in VMH): present (HMDB)<br>Remark: HMDB: present in all organisms, including bacteria.
MPP	N6,N6,N6-Trimethyl-L-lysine	N6,N6,N6-Trimethyllysine	HMDB0001325	TMLYS	6M	not reported to occur in any genera with variable importance ≥ 0.01
MPP	Guanidoacetic acid	Guanidoacetic acid	HMDB0000128	GUDAC	6M	not reported to occur in any genera with variable importance ≥ 0.01
MPP	Betaine	Betaine	HMDB0000043	Not Available	6M	not reported to occur in any genera with variable importance ≥ 0.01
MPP	5-Hydroxy-L-tryptophan	5-Hydroxytryptophan	HMDB0000472	5HTRP	6M, 12M	*Klebsiella*: present (VMH)<br>*Haemophilus*: present (VMH)<br>*Gemella*: present (VMH)<br>*Streptococcus*: present (VMH)<br>*Collinsella*: present (VMH)
MPP	unknown	Feature_mz_130.086	Not Available	Not Available	6M, 12M	not reported to occur in any genera with variable importance ≥ 0.01

Table S24. (continued)

platform	Compound name HMDB	Compound name reported	HMDB ID	VMH ID	visit(s)	occurs in …
MPP	Serotonin	Serotonin	HMDB0000259	SRTN	6M, 12M	*Klebsiella*: present (VMH + HMDB)<br>*Haemophilus*: present (VMH + HMDB)<br>*Gemella*: present (VMH + HMDB)<br>*Dialister*: present (HMDB)<br>*Hungatella*: present (HMDB)<br>*Lachnoclostridium*: present (HMDB)<br>*Bacteroides*: present (HMDB)<br>*Clostridium_sensu_stricto_1*: present (HMDB)<br>*Lachnospiraceae_unclassified*: present (HMDB)<br>*TM7x* (not in VMH): present (HMDB)<br>*Streptococcus*: present (VMH + HMDB)<br>*Collinsella*: present (VMH + HMDB)<br>*Erysipelatoclostridium*: present (HMDB)<br>*Robinsoniella* (not in VMH): present (HMDB)<br>Remark: HMDB: present in all organisms, including bacteria.
MPP	Riboflavin	Riboflavin	HMDB0000244	RIBFLV	6M	*Klebsiella*: present (VMH)<br>*Haemophilus*: present (VMH)<br>*Gemella*: present (VMH)<br>*Dialister*: present (VMH)<br>*Hungatella*: present (VMH)<br>*Lachnoclostridium*: present (VMH)<br>*Bacteroides*: present (VMH)<br>*Clostridium_sensu_stricto_1*: present (VMH)<br>*Lachnospiraceae_unclassified*: present (VMH)<br>*Streptococcus*: present (VMH)<br>*Collinsella*: present (VMH)<br>*Erysipelatoclostridium*: present (VMH)
MPP	Pyridoxal	Pyridoxal	HMDB0001545	PYDX	6M, 12M	*Klebsiella*: present (VMH)<br>*Haemophilus*: present (VMH)<br>Gemella: present (VMH)<br>Dialister: present (VMH)<br>Hungatella: present (VMH)<br>Lachnoclostridium: present (VMH)<br>Bacteroides: present (VMH)<br>Clostridium_sensu_stricto_1: present (VMH)<br>Lachnospiraceae_unclassified: present (VMH)<br>Streptococcus: present (VMH)<br>Collinsella: present (VMH)<br>Erysipelatoclostridium: present (VMH)

Table S24. (continued)

platform	Compound name HMDB	Compound name reported	HMDB ID	VMH ID	visit(s)	occurs in …
MPP	Picolinic acid	Picolinic acid	HMDB0002243	C10164	6M	not reported to occur in any genera with variable importance ≥ 0.01
MPP	L-Aspartic acid	Aspartic acid	HMDB0000191	ASP_L	6M	*Klebsiella*: present (VMH + HMDB), carbon source (VMH)<br>*Haemophilus* : present (VMH + HMDB)<br>*Gemella* : present (VMH + HMDB)<br>*Dialister* : present (VMH + HMDB)<br>*Hungatella* : present (VMH + HMDB)<br>*Lachnoclostridium* : present (VMH + HMDB)<br>*Bacteroides* : present (VMH + HMDB)<br>*Clostridium_sensu_stricto_1* : present (VMH + HMDB)<br>*Lachnospiraceae_unclassified* : present (VMH + HMDB)<br>*TM7x* (not in VMH) : present (HMDB)<br>*Streptococcus*: present (VMH + HMDB)<br>*Collinsella*: present (VMH + HMDB)<br>*Erysipelatoclostridium*: present (VMH + HMDB)<br>*Robinsoniella* (not in VMH): present (HMDB)<br>Remark: HMDB: present in all organisms, including bacteria.
MPP	Beta-Guanidinopropionic acid	Beta-Guanidinopropionic acid	HMDB0013222	Not Available	6M	not reported to occur in any genera with variable importance ≥ 0.01
MPP	5-Aminopentanoic acid	5-Aminopentanoic acid	HMDB0003355	5APTN	6M	*Klebsiella*: present (VMH)<br>*Gemella*: present (VMH)<br>*Dialister*: present (VMH)<br>*Hungatella*: present (VMH)<br>*Lachnoclostridium*: present (VMH)<br>*Clostridium_sensu_stricto_1*: present (VMH)
MPP	Uracil	Uracil	HMDB0000300	URA	6M	*Klebsiella*: present (VMH)<br>*Haemophilus*: present (VMH)<br>*Gemella*: present (VMH)<br>*Dialister*: present (VMH)<br>*Hungatella*: present (VMH)<br>*Lachnoclostridium*: present (VMH)<br>*Bacteroides*: present (VMH)<br>*Clostridium_sensu_stricto_1*: present (VMH)<br>*Lachnospiraceae_unclassified*: present (VMH)<br>*Streptococcus*: present (VMH)<br>*Collinsella*: present (VMH)<br>*Erysipelatoclostridium*: present (VMH)

Table S24. (continued)

platform	Compound name HMDB	Compound name reported	HMDB ID	VMH ID	visit(s)	occurs in …
MPP	N-Acetyl-L-tyrosine	N-Acetyltyrosine	HMDB0000866	Not Available	6M	not reported to occur in any genera with variable importance ≥ 0.01
MPP	L-Targinine/Homo-L-arginine	Targinine/Homoarginine	HMDB0029416	Not Available	12M	not reported to occur in any genera with variable importance ≥ 0.01
MPP	Ornithine	Ornithine	HMDB0000214	ORN	12M	*Klebsiella*: present (VMH + HMDB)<br>*Haemophilus*: present (VMH + HMDB)<br>*Gemella*: present (VMH + HMDB)<br>*Dialister*: present (VMH + HMDB)<br>*Hungatella*: present (VMH + HMDB)<br>*Lachnoclostridium*: present (VMH + HMDB)<br>*Bacteroides*: present (VMH + HMDB)<br>*Clostridium_sensu_stricto_1*: present (VMH + HMDB)<br>*Lachnospiraceae_unclassified*: present (VMH + HMDB)<br>*TM7x* (not in VMH): present (HMDB)<br>*Streptococcus*: present (VMH + HMDB)<br>*Collinsella*: present (VMH + HMDB)<br>*Erysipelatoclostridium*: present (VMH + HMDB)<br>*Robinsoniella* (not in VMH): Present (HMDB)<br>Remark: HMDB: present in all organisms, including bacteria.
MPP	L-Threonine/L-Homoserine	Threonine/Homoserine	HMDB0000167	THR_L	12M	*Klebsiella*: present (VMH)<br>*Haemophilus*: present (VMH)<br>*Gemella*: present (VMH)<br>*Dialister*: present (VMH)<br>*Hungatella*: present (VMH)<br>*Lachnoclostridium*: present (VMH)<br>*Bacteroides*: present (VMH)<br>*Clostridium_sensu_stricto_1*: present (VMH)<br>*Lachnospiraceae_unclassified*: present (VMH)<br>*Streptococcus*: present (VMH)<br>*Collinsella*: present (VMH)<br>*Erysipelatoclostridium*: present (VMH)

Table S24. (continued)

platform	Compound name HMDB	Compound name reported	HMDB ID	VMH ID	visit(s)	occurs in …
MPP	Thymine	Thymine	HMDB0000262	THYM	12M	*Klebsiella*: present (VMH + HMDB)<br>*Haemophilus*: present (VMH + HMDB)<br>*Gemella*: present (HMDB)<br>*Dialister*: present (HMDB)<br>*Hungatella*: present (VMH + HMDB)<br>*Lachnoclostridium*: present (VMH + HMDB)<br>*Bacteroides*: present (VMH + HMDB)<br>*Clostridium_sensu_stricto_1*: present (VMH + HMDB)<br>*Lachnospiraceae_unclassified*: present (VMH + HMDB)<br>*TM7x* (not in VMH): present (HMDB)<br>*Streptococcus*: present (VMH + HMDB)<br>*Collinsella*: present (VMH + HMDB)<br>*Erysipelatoclostridium*: present (HMDB)<br>*Robinsoniella* (not in VMH): present (HMDB)<br>Remark: HMDB: present in all organisms, including bacteria.
MPP	1-Methyladenosine/N6-Methyladenosine/2'-O-Methyladenosine	1-Methyladenosine/N6-Methyladenosine/2'-O-Methyladenosine	HMDB0003331	Not Available	12M	not reported to occur in any genera with variable importance ≥ 0.01
MPP	Ethanolamine	Ethanolamine	HMDB0000149	ETHA	12M	*Klebsiella*: present (VMH + HMDB)<br>*Haemophilus*: present (VMH + HMDB)<br>*Gemella*: present (VMH + HMDB)<br>*Dialister*: present (VMH + HMDB)<br>*Hungatella*: present (VMH + HMDB)<br>*Lachnoclostridium*: present (VMH + HMDB)<br>*Bacteroides*: present (VMH + HMDB)<br>*Clostridium_sensu_stricto_1*: present (VMH + HMDB)<br>*Lachnospiraceae_unclassified*: present (VMH + HMDB)<br>*TM7x* (not in VMH): present (HMDB)<br>*Streptococcus*: present (VMH + HMDB)<br>*Collinsella*: present (VMH + HMDB)<br>*Erysipelatoclostridium*: present (VMH + HMDB)<br>*Robinsoniella* (not in VMH): present (HMDB)<br>Remark: HMDB: present in all organisms, including bacteria.

Table S24. (continued)

platform	Compound name HMDB	Compound name reported	HMDB ID	VMH ID	visit(s)	occurs in …
MPP	Cadaverine	Cadaverine	HMDB0002322	15DAP	12M	*Klebsiella*: present (VMH)<br>*Hungatella*: present (VMH)<br>*Lachnoclostridium*: present (VMH)<br>*Bacteroides*: present (VMH)<br>*Lachnospiraceae_unclassified*: present (VMH)<br>*Streptococcus*: present (VMH)<br>*Erysipelatoclostridium*: present (VMH)
MPP	Sphinganine	Sphinganine	HMDB0000269	SPHGN	12M	*Klebsiella*: present (HMDB)<br>*Haemophilus*: present (HMDB)<br>*Gemella*: present (HMDB)<br>*Dialister*: present (HMDB)<br>*Hungatella*: present (HMDB)<br>*Lachnoclostridium*: present (HMDB)<br>*Bacteroides*: present (HMDB)<br>*Clostridium_sensu_stricto_1*: present (HMDB)<br>*Lachnospiraceae_unclassified*: present (HMDB)<br>*TM7x* (not in VMH): present (HMDB)<br>*Streptococcus*: present (HMDB)<br>*Collinsella*: present (HMDB)<br>*Erysipelatoclostridium*: present (HMDB)<br>*Robinsoniella* (not in VMH): present (HMDB)<br>Remark: HMDB: present in all organisms, including bacteria.
MPP	Deoxyguanosine	Deoxyguanosine	HMDB0000085	DGSN	12M	*Klebsiella*: present (VMH)<br>*Haemophilus*: present (VMH)<br>*Gemella*: present (VMH)<br>*Dialister*: present (VMH)<br>*Hungatella*: present (VMH)<br>*Lachnoclostridium*: present (VMH)<br>*Bacteroides*: present (VMH)<br>*Clostridium_sensu_stricto_1*: present (VMH)<br>*Lachnospiraceae_unclassified*: present (VMH)<br>*Streptococcus*: present (VMH)<br>*Collinsella*: present (VMH)<br>*Erysipelatoclostridium*: present (VMH)

Table S24. (continued)

platform	Compound name HMDB	Compound name reported	HMDB ID	VMH ID	visit(s)	occurs in …
MPP	5-Aminolevulinic acid/4-Hydroxyproline	5-Aminolevulinic acid/4-Hydroxyproline	HMDB0001149	5AOP	12M	*Klebsiella*: present (VMH + HMDB)<br>*Haemophilus*: present (VMH + HMDB)<br>*Gemella*: present (HMDB)<br>*Dialister*: present (VMH + HMDB)<br>*Hungatella*: present (VMH + HMDB)<br>*Lachnoclostridium*: present (VMH + HMDB)<br>*Bacteroides*: present (VMH + HMDB)<br>*Clostridium_sensu_stricto_1*: present (VMH + HMDB)<br>*Lachnospiraceae_unclassified*: present (VMH + HMDB)<br>*TM7x* (not in VMH): present (HMDB)<br>*Streptococcus*: present (VMH + HMDB)<br>*Collinsella*: present (VMH + HMDB)<br>*Erysipelatoclostridium*: present (VMH + HMDB)<br>*Robinsoniella* (not in VMH): present (HMDB)<br>Remark: HMDB: present in all organisms, including bacteria.
MPP	N2,N2-Dimethylguanosine	N2,N2-Dimethylguanosine	HMDB0004824	Not Available	12M	not reported to occur in any genera with variable importance ≥ 0.01
MPP	Cytidine	Cytidine	HMDB0000089	CYTD	12M	*Klebsiella*: present (VMH)<br>*Haemophilus*: present (VMH)<br>*Gemella*: present (VMH)<br>*Dialister*: present (VMH)<br>*Hungatella*: present (VMH)<br>*Lachnoclostridium*: present (VMH)<br>*Bacteroides*: present (VMH)<br>*Clostridium_sensu_stricto_1*: present (VMH)<br>*Lachnospiraceae_unclassified*: present (VMH)<br>*Streptococcus*: present (VMH)<br>*Collinsella*: present (VMH)<br>*Erysipelatoclostridium*: present (VMH)

Table S24. (continued)

platform	Compound name HMDB	Compound name reported	HMDB ID	VMH ID	visit(s)	occurs in …
MPP	Thiamine	Thiamine	HMDB0000235	THM	12M	*Klebsiella*: present (VMH + HMDB)<br>*Haemophilus*: present (VMH + HMDB)<br>*Gemella*: present (VMH + HMDB)<br>*Dialister*: present (VMH + HMDB)<br>*Hungatella*: present (VMH + HMDB)<br>*Lachnoclostridium*: present (VMH + HMDB)<br>*Bacteroides*: present (VMH + HMDB)<br>*Clostridium_sensu_stricto_1*: present (VMH + HMDB)<br>*Lachnospiraceae_unclassified*: present (VMH + HMDB)<br>*TM7x* (not in VMH): present (HMDB)<br>*Streptococcus*: present (VMH + HMDB)<br>*Collinsella*: present (VMH + HMDB)<br>*Erysipelatoclostridium*: present (VMH + HMDB)<br>*Robinsoniella* (not in VMH): present (HMDB)<br>Remark: HMDB: present in all organisms, including bacteria.

Table S25. Pathway information (KEGG) for the microbial protein groups, immune factors and metabolites with variable importance ≥ 0.01.

Platform	Visit	Reported_variable_name	Uniprot, NCBI or HMDB ID	KO or compound ID	KEGG pathways
microbial metaproteomics	baseline	IMP cyclohydrolase [Clostridiales]<br>IMP cyclohydrolase [Blautia glucerasea]<br>IMP cyclohydrolase [Clostridiales]<br>IMP cyclohydrolase - Extibacter muris<br>IMP cyclohydrolase [Merdimonas faecis]<br>IMP cyclohydrolase-like protein - Anaerostipes hadrus<br>IMP cyclohydrolase - Eisenbergiella porci<br>IMP cyclohydrolase-like protein - Blautia obeum<br>IMP cyclohydrolase - Enterocloster asparagiformis<br>IMP cyclohydrolase - Eisenbergiella massiliensis<br>IMP cyclohydrolase-like protein - Faecalicatena orotica<br>IMP cyclohydrolase - Enterocloster lavalensis<br>IMP cyclohydrolase - Anaerostipes hadrus<br>IMP cyclohydrolase - Blautia producta<br>IMP cyclohydrolase - Eisenbergiella tayi<br>IMP cyclohydrolase - Ruminococcus bromii<br>IMP cyclohydrolase [Blautia]	WP_008705693.1<br>WP_173726912.1<br>WP_117854094.1<br>A0A4R4FB20<br>WP_070087401.1<br>D4MZD9<br>A0A6N7WM25<br>A5ZQR8<br>A0A413FLR6<br>A0A3E3I955<br>A0A2Y9CAV0<br>A0A1I0AVI8<br>A0A173XPM4<br>A0A6P1Z5F2<br>A0A1E3AC12<br>A0A413QC06<br>WP_173752049.1	K11176	Purine metabolism; Biosynthesis of secondary metabolites
microbial metaproteomics	baseline	DNA-directed RNA polymerase subunit beta - Bifidobacterium longum<br>DNA-directed RNA polymerase subunit beta - Bifidobacterium longum subsp. Infantis<br>DNA-directed RNA polymerase subunit beta - Bifidobacterium longum subsp. Infantis<br>DNA-directed RNA polymerase subunit beta - Bifidobacterium angulatum<br>DNA-directed RNA polymerase subunit beta - Bifidobacterium animalis subsp. lactis (Bifidobacterium lactis)<br>DNA-directed RNA polymerase subunit beta - Bifidobacterium pullorum subsp. Saeculare<br>DNA-directed RNA polymerase subunit beta - Bifidobacterium pullorum<br>DNA-directed RNA polymerase subunit beta, partial - Bifidobacterium pullorum subsp. saeculare	A0A0A1GRX3<br>A0A1S2VY79<br>A0A0M3T5H1<br>A0A126ST81<br>A0A315S068<br>KFI89168.1<br>WP_051912151.1<br>AUR34142.1	K03043	RNA polymerase

Table S25. (continued)

Platform	Visit	Reported_variable_name	Uniprot, NCBI or HMDB ID	KO or compound ID	KEGG pathways
microbial metaproteomics	baseline	Class II fructose-1,6-bisphosphate aldolase - Anaerostipes hadrus<br>Class II fructose-1,6-bisphosphate aldolase [Lacrimispora amygdalina]	A0A5B3GKY1<br><br>WP_144365668.1	K01624	Glycolysis / Gluconeogenesis; Pentose phosphate pathway; Methane metabolism; Biosynthesis of secondary metabolites; Carbon metabolism; Biosynthesis of amino acids
microbial metaproteomics	baseline	GGGtGRT protein [Clostridiales]<br>Uncharacterized protein - Blautia obeum<br>GGGtGRT protein - Blautia Luti<br>hypothetical protein [Clostridiales]<br>GGGtGRT protein - Ruminococcus flavefaciens<br>GGGtGRT protein - Ruminococcus flavefaciens<br>GGGtGRT protein - Blautia producta<br>GGGtGRT protein - Blautia faecicola<br>GGGtGRT protein - Clostridium chromiireducens	WP_015525727.1<br>A5ZUI6<br>A0A564W402<br>WP_008707497.1<br>A0A1K1PRP2<br><br>A0A1H6I7Y0<br>A0A6P1ZBB0<br>A0A4Q1RH04<br><br>A0A1V4ID03	Not available	Not available
microbial metaproteomics	baseline	50S ribosomal protein L5 - Anaerostipes hadrus<br>50S ribosomal protein L5 [Eubacteriales]<br>50S ribosomal protein L5 - Clostridium perfringens<br>50S ribosomal protein L5 - Faecalicatena orotica<br>50S ribosomal protein L5 - Lachnospira pectinoschiza	A0A173R7M3<br><br>WP_070087926.1<br>Q0TMQ8<br><br>A0A2Y9BM50<br><br>A0A174LMQ2	K02931	Ribosome

Table S25. (continued)

Platform	Visit	Reported_variable_name	Uniprot, NCBI or HMDB ID	KO or compound ID	KEGG pathways
microbial metaproteomics	baseline	50S ribosomal protein L16 - Blautia faecicola	A0A4V1NS38	K02878	Ribosome
		50S ribosomal protein L16 - Blautia luti	A0A564W4Q7		
		50S ribosomal protein L16 - Blautia producta	A0A6P1Z5F6		
		50S ribosomal protein L16 [Blautia]	WP_008707261.1		
		50S ribosomal protein L16 [Eubacteriales]	WP_118062095.1		
		50S ribosomal protein L16 [Blautia]	WP_158421032.1		
		50S ribosomal protein L16 [Eubacteriales]	WP_117852531.1		
		50S ribosomal protein L16 [Eubacteriales];	WP_015553837.1		
		50S ribosomal protein L16 [Mediterraneibacter glycyrrhizinilyticus]	WP_009267277.1;		
		50S ribosomal protein L16 - Roseburia inulinivorans	C0FTI7		
		50S ribosomal protein L16 - Enterocloster asparagiformis	A0A413F9M6		
		50S ribosomal protein L16 - Enterocloster aldenensis	A0A3E2WBN9		
		50S ribosomal protein L16 - Hungatella effluvii	A0A2V3YPY2		
		50S ribosomal protein L16 - Enterocloster lavalensis	A0A1I0HXR3		
		50S ribosomal protein L16 - Clostridium symbiosum (Bacteroides symbiosus)	A0A174GGM7		
		50S ribosomal protein L16 [Eubacteriales]	WP_097002538.1		
		50S ribosomal protein L16 - Roseburia faecis	A0A0M6WYV2		
		50S ribosomal protein L16 - Hungatella hathewayi	A0A174ADQ2		
		50S ribosomal protein L16 - Hungatella xylanolytica	A0A2S6HH86		
		50S ribosomal protein L16 [Blautia]	WP_022067329.1		
		50S ribosomal protein L16 - Blautia obeum	A5ZW85		
		50S ribosomal protein L16 - Faecalicatena orotica	A0A2Y9BG59		
		50S ribosomal protein L16 - Lachnospira pectinoschiza	A0A1G9XH09		
		50S ribosomal protein L16 - Lachnospira pectinoschiza	A0A174LQZ1		
		50S ribosomal protein L16 [Eubacteriales]	WP_070087931.1		
		50S ribosomal protein L16 [Eubacteriales]	WP_173771785.1		
		50S ribosomal protein L16 [Lachnospira]	WP_027430844.1		

Table S25. (continued)

Platform	Visit	Reported_variable_name	Uniprot, NCBI or HMDB ID	KO or compound ID	KEGG pathways
immune data	6M	Eukaryotic translation initiation factor 4E-binding protein 1 (4E-BP1)	Q13541	K07205	ErbB signaling pathway; HIF-1 signaling pathway; mTOR signaling pathway; PI3K-Akt signaling pathway; AMPK signaling pathway; Longevity regulating pathway; Cellular senescence; Insulin signaling pathway
immune data	6M	Interleukin-1 alpha (IL-1 alpha)	P01583	K04383	MAPK signaling pathway; Cytokine-cytokine receptor interaction; Necroptosis; Cellular senescence; Osteoclast differentiation; Hematopoietic cell lineage
immune data	6M	C-X-C motif chemokine 5 (CXCL5)	P42830	K05506	Cytokine-cytokine receptor interaction; Chemokine signaling pathway; IL-17 signaling pathway; TNF signaling pathway
immune data	6M	C-C motif chemokine 4 (CCL4)	P13236	K12964	Cytokine-cytokine receptor interaction; Chemokine signaling pathway; NF-kappa B signaling pathway; Toll-like receptor signaling pathway; Cytosolic DNA-sensing pathway
immune data	6M	Monocyte chemotactic protein 1 (MCP-1)	P13500	K14624	Cytokine-cytokine receptor interaction; Chemokine signaling pathway; NOD-like receptor signaling pathway; IL-17 signaling pathway; TNF signaling pathway;
immune data	6M	Interleukin-12 subunit beta (IL-12B)	P29460	K05425	Cytokine-cytokine receptor interaction; Toll-like receptor signaling pathway; RIG-I-like receptor signaling pathway; C-type lectin receptor signaling pathway; JAK-STAT signaling pathway; Th1 and Th2 cell differentiation

Table S25. (continued)

Platform	Visit	Reported_variable_name	Uniprot, NCBI or HMDB ID	KO or compound ID	KEGG pathways
immune data	6M	Transforming growth factor alpha (TGF-alpha)	P01135	K08774	EGFR tyrosine kinase inhibitor resistance; MAPK signaling pathway; ErbB signaling pathway; Ras signaling pathway; Calcium signaling pathway; PI3K-Akt signaling pathway; Estrogen signaling pathway
immune data	6M	Programmed cell death 1 ligand 1 (PD-L1)	Q9NZQ7	K06745	Cell adhesion molecules
immune data	6M	Interleukin-15 receptor subunit alpha (IL-15RA)	Q13261	K05074	Cytokine-cytokine receptor interaction; JAK-STAT signaling pathway
immune data	6M	Latency-associated peptide transforming growth factor beta-1 (LAP TGF-beta-1)	P01137	K13375	MAPK signaling pathway; Cytokine-cytokine receptor interaction; FoxO signaling pathway; Cell cycle; Efferocytosis; Cellular senescence; TGF-beta signaling pathway; Osteoclast differentiation; Hippo signaling pathway; Th17 cell differentiation; Relaxin signaling pathway
immune data	6M	STAM-binding protein (STAMBP)	O95630	K11866	Endocytosis
immune data	6M	Protein S100-A12 (EN-RAGE)	P80511	K23766	Not available
immune data	6M	Caspase-8 (CASP-8)	Q14790	K04398	p53 signaling pathway; Apoptosis; Necroptosis; Toll-like receptor signaling pathway; NOD-like receptor signaling pathway; RIG-I-like receptor signaling pathway; Cytosolic DNA-sensing pathway; Toll and Imd signaling pathway; C-type lectin receptor signaling pathway; IL-17 signaling pathway; TNF signaling pathway
immune data	6M	TNF-related apoptosis-inducing ligand (TRAIL)	P50591	K04721	Cytokine-cytokine receptor interaction; FoxO signaling pathway; Apoptosis; Necroptosis; Natural killer cell mediated cytotoxicity

Table S25. (continued)

Platform	Visit	Reported_variable_name	Uniprot, NCBI or HMDB ID	KO or compound ID	KEGG pathways
immune data	6M	Tumor necrosis factor receptor superfamily member 9 (TNFRSF9)	Q07011	K05146	Cytokine-cytokine receptor interaction
immune data	6M	Macrophage colony-stimulating factor 1 (CSF-1)	P09603	K05453	MAPK signaling pathway; Ras signaling pathway; Rap1 signaling pathway; Cytokine-cytokine receptor interaction; PI3K-Akt signaling pathway; Osteoclast differentiation; Hematopoietic cell lineage; TNF signaling pathway
immune data	6M	Osteoprotegerin (OPG)	O00300	K05148	Cytokine-cytokine receptor interaction; Osteoclast differentiation
immune data	6M	Leukemia inhibitory factor receptor (LIF-R)	P42702	K05058	Cytokine-cytokine receptor interaction; Signaling pathways regulating pluripotency of stem cells; JAK-STAT signaling pathway
immune data	6M	C-C motif chemokine 3 (CCL3)	P10147	K05408	Cytokine-cytokine receptor interaction; Chemokine signaling pathway; Toll-like receptor signaling pathway
immune data	6M	Monocyte chemotactic protein 4 (MCP-4)	Q99616	K16595	Cytokine-cytokine receptor interaction; Chemokine signaling pathway; NF-kappa B signaling pathway
immune data	6M	Matrix metalloproteinase-1 (MMP-1)	P03956	K01388	PPAR signaling pathway; IL-17 signaling pathway; Relaxin signaling pathway
immune data	6M	Fibroblast growth factor 19 (FGF-19)	O95750	K22603	MAPK signaling pathway; Ras signaling pathway; Rap1 signaling pathway; Calcium signaling pathway; PI3K-Akt signaling pathway; Regulation of actin cytoskeleton

Table S25. (continued)

Platform	Visit	Reported_variable_name	Uniprot, NCBI or HMDB ID	KO or compound ID	KEGG pathways
immune data	6M	Tumor necrosis factor (TNF)	P01375	K03156	MAPK signaling pathway; Cytokine-cytokine receptor interaction; NF-kappa B signaling pathway; Sphingolipid signaling pathway; mTOR signaling pathway; Apoptosis; Necroptosis; TGF-beta signaling pathway; Osteoclast differentiation; Antigen processing and presentation; Toll-like receptor signaling pathway; NOD-like receptor signaling pathway; RIG-I-like receptor signaling pathway; C-type lectin receptor signaling pathway; Hematopoietic cell lineage; Natural killer cell mediated cytotoxicity; IL-17 signaling pathway; T cell receptor signaling pathway; Fc epsilon RI signaling pathway; TNF signaling pathway; Adipocytokine signaling pathway
immune data	6M	Vascular endothelial growth factor A (VEGF-A)	P15692	K05448	MAPK signaling pathway; Ras signaling pathway; Rap1 signaling pathway; Calcium signaling pathway; HIF-1 signaling pathway; PI3K-Akt signaling pathway; VEGF signaling pathway; Focal adhesion; Relaxin signaling pathway
immune data	6M	C-C motif chemokine 28 (CCL28)	Q9NRJ3	K05513	Cytokine-cytokine receptor interaction; Chemokine signaling pathway
immune data	6M	Interleukin-7 (IL-7)	P13232	K05431	Cytokine-cytokine receptor interaction; PI3K-Akt signaling pathway; JAK-STAT signaling pathway; Hematopoietic cell lineage

Table S25. (continued)

Platform	Visit	Reported_variable_name	Uniprot, NCBI or HMDB ID	KO or compound ID	KEGG pathways
immune data	6M	Oncostatin-M (OSM)	P13725	K05418	Cytokine-cytokine receptor interaction; PI3K-Akt signaling pathway; JAK-STAT signaling pathway
immune data	6M	Fms-related tyrosine kinase 3 ligand (Flt3L)	P49771	K05454	MAPK signaling pathway; Ras signaling pathway; PI3K-Akt signaling pathway; Hematopoietic cell lineage
immune data	6M	Interleukin-10 receptor subunit beta (IL-10RB)	Q08334	K05135	Cytokine-cytokine receptor interaction; JAK-STAT signaling pathway
immune data	6M	C-C motif chemokine 19 (CCL19)	Q99731	K05512	Cytokine-cytokine receptor interaction; Chemokine signaling pathway; NF-kappa B signaling pathway
platform for polar to semi-polar metabolites - negative mode	6M	myo-Inositol/Galactose/Fructose	HMDB0000211	C00137	Galactose metabolism; Ascorbate and aldarate metabolism; Streptomycin biosynthesis; Inositol phosphate metabolism; Biosynthesis of secondary metabolites; Biosynthesis of nucleotide sugars; ABC transporters; Phosphatidylinositol signaling system

Table S25. (continued)

Platform	Visit	Reported_variable_name	Uniprot, NCBI or HMDB ID	KO or compound ID	KEGG pathways
platform for polar to semi-polar metabolites - negative mode	6M	Protocatechuic acid	HMDB0001856	C00230	Benzoate degradation; Phenylalanine, tyrosine and tryptophan biosynthesis; Toluene degradation; Polycyclic aromatic hydrocarbon degradation; Aminobenzoate degradation; Biosynthesis of siderophore group nonribosomal peptides; Biosynthesis of phenylpropanoids; Biosynthesis of secondary metabolites; Degradation of aromatic compounds
platform for polar to semi-polar metabolites - negative mode	6M	Pyrocatechol	HMDB0000957	C15571	Not available
platform for polar to semi-polar metabolites - negative mode	6M	Phenylacetic acid	HMDB0000209	C07086	Phenylalanine metabolism; Styrene degradation
platform for polar to semi-polar metabolites - negative mode	6M	3-Hydroxybutyric acid	HMDB0000357	C01089	Butanoate metabolism; cAMP signaling pathway
platform for polar to semi-polar metabolites - negative mode	6M	N6-Carboxymethyllysine	HMDB0240347	Not available	Not available
platform for polar to semi-polar metabolites - negative mode	6M	Histidine	HMDB0000177	C00135	Histidine metabolism; Staurosporine biosynthesis; beta-Alanine metabolism; D-Amino acid metabolism; Aminoacyl-tRNA biosynthesis; Biosynthesis of alkaloids derived from histidine and purine; Biosynthesis of secondary metabolites; Biosynthesis of amino acids; ABC transporters; Protein digestion and absorption

Table S25. (continued)

Platform	Visit	Reported_variable_name	Uniprot, NCBI or HMDB ID	KO or compound ID	KEGG pathways
platform for polar to semi-polar metabolites - negative mode	6M	Syringic acid	HMDB0002085	C10833	Aminobenzoate degradation
platform for polar to semi-polar metabolites - negative mode	6M	trans-Aconitic acid	HMDB0000958	C02341	C5-Branched dibasic acid metabolism
platform for polar to semi-polar metabolites - negative mode	6M	Phenylacetylglutamine	HMDB0006344	C04148	Phenylalanine metabolism
platform for polar to semi-polar metabolites - negative mode	6M	N-Acetylneuraminic acid	HMDB0000230	C19910	Not available
platform for polar to semi-polar metabolites - negative mode	6M	2,5-Furandicarboxylic acid	HMDB0004812	C20450	Furfural degradation
platform for polar to semi-polar metabolites - negative mode	6M	FAD	HMDB0001248	C00016	Riboflavin metabolism; Biosynthesis of secondary metabolites; Biosynthesis of cofactors; Vitamin digestion and absorption
platform for polar to semi-polar metabolites - negative mode	6M	N-Acetylglutamine	HMDB0006029	Not available	Not available
platform for polar to semi-polar metabolites - negative mode	6M	Gluconic acid	HMDB0000625	C00257	Pentose phosphate pathway; Biosynthesis of secondary metabolites; Carbon metabolism
platform for polar to semi-polar metabolites - negative mode	6M	2-Hydroxyethanesulfonate	HMDB0003903	C05123	Taurine and hypotaurine metabolism
platform for polar to semi-polar metabolites - negative mode	6M	Pseudouridine	HMDB0000767	C02067	Pyrimidine metabolism
platform for polar to semi-polar metabolites - negative mode	6M	Xylulose	HMDB0001644	C00310	Pentose and glucuronate interconversions
platform for polar to semi-polar metabolites - positive mode	6M	N1-Methyl-4-pyridone-3-carboxamide/Nudifloramide	HMDB0004194	C05843	Nicotinate and nicotinamide metabolism

Table S25. (continued)

Platform	Visit	Reported_variable_name	Uniprot, NCBI or HMDB ID	KO or compound ID	KEGG pathways
platform for polar to semi-polar metabolites - positive mode	6M, 12M	Citrulline	HMDB0000904	C00327	Arginine biosynthesis; Biosynthesis of secondary metabolites; Biosynthesis of amino acids
platform for polar to semi-polar metabolites - positive mode	6M	Dodecanoylcarnitine	HMDB0002250	Not available	Not available
platform for polar to semi-polar metabolites - positive mode	6M	Dihydrouracil	HMDB0000076	C00429	Pyrimidine metabolism; beta-Alanine metabolism; Pantothenate and CoA biosynthesis
platform for polar to semi-polar metabolites - positive mode	6M	N6,N6,N6-Trimethyllysine	HMDB0001325	C03793	Lysine degradation
platform for polar to semi-polar metabolites - positive mode	6M	Guanidoacetic acid	HMDB0000128	C00581	Glycine, serine and threonine metabolism; Arginine and proline metabolism
platform for polar to semi-polar metabolites - positive mode	6M	Betaine	HMDB0000043	C00719	Glycine, serine and threonine metabolism; Metabolic pathways; ABC transporters
platform for polar to semi-polar metabolites - positive mode	6M, 12M	5-Hydroxytryptophan	HMDB0000472	C00643	Tryptophan metabolism; Biosynthesis of alkaloids derived from shikimate pathway
platform for polar to semi-polar metabolites - positive mode	6M, 12M	Feature_mz_130.086	Not Available	Not Available	Not Available
platform for polar to semi-polar metabolites - positive mode	6M, 12M	Serotonin	HMDB0000259	C00780	Tryptophan metabolism; Biosynthesis of alkaloids derived from shikimate pathway; Biosynthesis of secondary metabolites; cAMP signaling pathway; Gap junction; Inflammatory mediator regulation of TRP channels; Serotonin receptor agonists/antagonists

Table S25. (continued)

Platform	Visit	Reported_variable_name	Uniprot, NCBI or HMDB ID	KO or compound ID	KEGG pathways
platform for polar to semi-polar metabolites - positive mode	6M	Riboflavin	HMDB0000244	C00255	Riboflavin metabolism; Biosynthesis of secondary metabolites; Biosynthesis of cofactors; ABC transporters; Vitamin digestion and absorption
platform for polar to semi-polar metabolites - positive mode	6M, 12M	Pyridoxal	HMDB0001545	C00250	Vitamin B6 metabolism; Biosynthesis of cofactors; Vitamin digestion and absorption
platform for polar to semi-polar metabolites - positive mode	6M	Picolinic acid	HMDB0002243	C10164	Tryptophan metabolism

Table S25. (continued)

Platform	Visit	Reported_variable_name	Uniprot, NCBI or HMDB ID	KO or compound ID	KEGG pathways
platform for polar to semi-polar metabolites - positive mode	6M	Aspartic acid	HMDB0000191	C00049	Arginine biosynthesis; Alanine, aspartate and glutamate metabolism; Glycine, serine and threonine metabolism; Monobactam biosynthesis; Cysteine and methionine metabolism; Lysine biosynthesis; Histidine metabolism; beta-Alanine metabolism; D-Amino acid metabolism; Nicotinate and nicotinamide metabolism; Pantothenate and CoA biosynthesis; Aminoacyl-tRNA biosynthesis; Biosynthesis of various other secondary metabolites; Biosynthesis of alkaloids derived from ornithine, lysine and nicotinic acid; Biosynthesis of secondary metabolites; Carbon metabolism; 2-Oxocarboxylic acid metabolism; Biosynthesis of amino acids; Biosynthesis of cofactors; ABC transporters; Two-component system; Bacterial chemotaxis; Protein digestion and absorption
platform for polar to semi-polar metabolites - positive mode	6M	Beta-Guanidinopropionic acid	HMDB0013222	C03065	Not available
platform for polar to semi-polar metabolites - positive mode	6M	5-Aminopentanoic acid	HMDB0003355	C00431	Lysine degradation; Arginine and proline metabolism; D-Amino acid metabolism

Table S25. (continued)

Platform	Visit	Reported_variable_name	Uniprot, NCBI or HMDB ID	KO or compound ID	KEGG pathways
platform for polar to semi-polar metabolites - positive mode	6M	Uracil	HMDB0000300	C00106	Pyrimidine metabolism; beta-Alanine metabolism; Pantothenate and CoA biosynthesis; Nucleotide metabolism
platform for polar to semi-polar metabolites - positive mode	6M	N-Acetyltyrosine	HMDB0000866	C01657	Not available
platform for polar to semi-polar metabolites - positive mode	12M	Targinine/Homoarginine	HMDB0029416	C03884	Not available
platform for polar to semi-polar metabolites - positive mode	12M	Ornithine	HMDB0000214	C00077	Arginine biosynthesis; Arginine and proline metabolism; D-Amino acid metabolism; Glutathione metabolism; Biosynthesis of various other secondary metabolites; Biosynthesis of alkaloids derived from ornithine, lysine and nicotinic acid; Biosynthesis of secondary metabolites; 2-Oxocarboxylic acid metabolism; Biosynthesis of amino acids; ABC transporters; Efferocytosis

Table S25. (continued)

Platform	Visit	Reported_variable_name	Uniprot, NCBI or HMDB ID	KO or compound ID	KEGG pathways
platform for polar to semi-polar metabolites - positive mode	12M	Threonine/Homoserine	HMDB0000167	C00188	Glycine, serine and threonine metabolism; Monobactam biosynthesis; Valine, leucine and isoleucine biosynthesis; D-Amino acid metabolism; Porphyrin metabolism; Aminoacyl-tRNA biosynthesis; Biosynthesis of secondary metabolites; Biosynthesis of amino acids; ABC transporters; Protein digestion and absorption; Mineral absorption
platform for polar to semi-polar metabolites - positive mode	12M	Thymine	HMDB0000262	C00178	Pyrimidine metabolism; Nucleotide metabolism
platform for polar to semi-polar metabolites - positive mode	12M	1-Methyladenosine/N6-Methyladenosine/2'-O-Methyladenosine	HMDB0003331	C02494	Not available
platform for polar to semi-polar metabolites - positive mode	12M	Ethanolamine	HMDB0000149	C00189	Glycerophospholipid metabolism; Retrograde endocannabinoid signaling
platform for polar to semi-polar metabolites - positive mode	12M	Cadaverine	HMDB0002322	C01672	Lysine degradation; D-Amino acid metabolism; Glutathione metabolism; Tropane, piperidine and pyridine alkaloid biosynthesis; Biosynthesis of alkaloids derived from ornithine; lysine and nicotinic acid; Biosynthesis of secondary metabolites; Protein digestion and absorption
platform for polar to semi-polar metabolites - positive mode	12M	Sphinganine	HMDB0000269	C00836	Sphingolipid metabolism; Sphingolipid signaling pathway
platform for polar to semi-polar metabolites - positive mode	12M	Deoxyguanosine	HMDB0000085	C00330	Purine metabolism; Nucleotide metabolism; ABC transporters

Table S25. (continued)

Platform	Visit	Reported_variable_name	Uniprot, NCBI or HMDB ID	KO or compound ID	KEGG pathways
platform for polar to semi-polar metabolites - positive mode	12M	5-Aminolevulinic acid/4-Hydroxyproline	HMDB0001149	C00430	Glycine, serine and threonine metabolism; Porphyrin metabolism; Biosynthesis of secondary metabolites; Biosynthesis of cofactors; ABC transporters
platform for polar to semi-polar metabolites - positive mode	12M	N2,N2-Dimethylguanosine	HMDB0004824	Not available	Not available
platform for polar to semi-polar metabolites - positive mode	12M	Cytidine	HMDB0000089	C00475	Pyrimidine metabolism; Nucleotide metabolism; ABC transporters
platform for polar to semi-polar metabolites - positive mode	12M	Thiamine	HMDB0000235	C00378	Thiamine metabolism; Biosynthesis of cofactors; ABC transporters; Sulfur relay system; Vitamin digestion and absorption

Table S26. Variable importance per view, based on mean decrease in node impurity (Gini index) (training sets). Top 10 for all visits in one view (approach 1), and top 10 per visit (approach 2) based on mean variable importance.

	variable	all visits	0M	6M	12M
clinical data (abbreviations: see Table S1)	SCORADMAN	x	x	x	x
	alrgyfatY	x	x	x	x
	age	x	x	x	x
	treatmentB	x			
	SPTOWFPositive	x	x	x	
	alrgymotY	x	x	x	
	deliveryVaginal	x	x		
	FOODTRIG2Y	x	x		
	num_inf	x		x	x
	num_ab	x			x
	siblY		x		
	SPTOPPositive		x		
	sexM		x		
	STOOLCONSIST			x	x
	STOOLCOLOUR			x	x
	STOOLFREQ			x	x
	GASWIND			x	x
	VOMITING				x
16S rRNA gene sequencing	*Haemophilus*	x	x	x	x
	Veillonella	x		x	x
	Klebsiella	x	x		
	Lachnoclostridium	x	x		x
	Hungatella	x	x	x	x
	Faecalibacterium	x		x	
	Eisenbergiella	x			x
	Blautia	x		x	
	Erysipelatoclostridium	x	x		
	Bacteroides	x	x		x
	Clostridium_sensu_stricto_1		x		
	Streptococcus		x		
	Lachnospiraceae_unclassified		x		
	TM7x		x		
	Veillonellaceae_unclassified			x	
	Abiotrophia			x	
	Erysipelotrichaceae_ge			x	
	Butyricicoccus			x	
	Eggerthella			x	
	Enterococcus				x
	Subdoligranulum				x
	Anaerostipes				x
	Alistipes				x

Table S26. (continued)

	variable	all visits	0M	6M	12M
metaproteomics – microbial proteins* (abbreviated fasta maxquant, for complete fasta maxquant, see footnotes of the table)	tr.A0A3E4U7M2	x		x	
	tr.A0A3E3EA02	x			x
	tr.A0A369M4T5	x		x	
	tr.A0A5B3GKY1	x	x		
	tr.A0A151C8B3	x			
	tr.WP_119239543.1	x			
	tr.A0A0A1GRX3	x	x		
	tr.E5XVX1	x			x
	tr.A0A2Y9B7L8	x			
	tr.WP_008703889.1	x			
	tr.A0A1V8Q6G3		x		
	tr.D1PG79		x		
	tr.WP_008705693.1		x		
	tr.A0A173R7M3		x		
	tr.WP_173773355.1		x		
	tr.WP_008702995.1		x		
	tr.WP_025577282.1		x		
	tr.A0A1L8SSH0		x		
	tr.A0A0H2PUX6			x	
	tr.D4BR49			x	
	tr.A0A1Q2CBB9			x	
	tr.A0A4S5BCM0			x	
	tr.WP_070089094.1			x	
	tr.WP_025578671.1			x	
	tr.WP_028255879.1			x	
	tr.A0A3E3EF25			x	
	tr.WP_020993932.1				x
	tr.A6L7K3				x
	tr.WP_025580226.1				x
	tr.A0A329TVD2				x
	tr.A0A4V2X185				x
	tr.WP_147598065.1				x
	tr.A0A3E3EA89				x
	tr.WP_022067817.1				x

Table S26. (continued)

	variable	all visits	0M	6M	12M
metaproteomics – human proteins* (abbreviated fasta maxquant, for complete fasta maxquant, see footnotes of the table)	sp.P01701	x	x	x	
	sp.P06702	x		x	x
	sp.A0A075B6H9	x	x		
	sp.P01024	x			x
	sp.P01703	x			
	sp.A0A0C4DH72	x	x	x	
	sp.A0A0A0MT36	x		x	
	sp.P02810	x			
	sp.P01834	x			
	sp.Q86UP6	x		x	x
	sp.P15924		x		x
	sp.Q9UGM3		x		
	sp.P68133		x		
	sp.Q5D862		x		
	sp.Q86YZ3		x		x
	sp.P01009		x		x
	sp.P09923		x		
	sp.Q9Y6R7			x	
	sp.P15085			x	
	sp.P01764			x	
	sp.P68133			x	
	sp.P04745			x	
	sp.P09093				x
	sp.Q8WWU7				x
	sp.P21796				x
	sp.A0A0J9YXX1				x

Table S26. (continued)

	variable	all visits	0M	6M	12M
immune data** (abbreviations: see footnotes)	CXCL5	x	x	x	x
	4E-BP1	x	x	x	
	MMP-10	x	x		
	IL18	x	x		x
	EN-RAGE	x	x	x	
	IL-15RA	x	x		x
	IL8	x			x
	CX3CL1	x			x
	IL-1-alpha	x		x	
	IL-20RA	x			
	MMP-1		x		
	LIF-R		x		
	Flt3L		x		
	CASP-8		x		
	CCL4			x	
	PD-L1			x	
	MCP-4			x	
	TNFSF14			x	
	TNFRSF9			x	x
	LIF			x	
	uPA				x
	IL7				x
	OSM				x
	IL-12B				x

Table S26. (continued)

	variable	all visits	0M	6M	12M
metabolomics - platform for polar to semi-polar metabolites in negative mode	protocatechuic acid	x		x	
	myo-inositol / galactose / fructose	x	x	x	
	pyrocatechol	x		x	
	lysine	x			x
	orotic acid	x			x
	o-acetylserine / glutamic acid	x		x	
	trans-aconitic acid	x		x	
	3-hydroxybenzoic acid	x			
	4-hydroxycinnamic acid	x			x
	1-methyluric acid	x			
	xylulose		x		
	thymidine		x		
	desaminotyrosine		x		
	phenyllactic acid		x		
	malic acid		x		
	methionine sulfoxide		x		
	3-(3-hydroxyphenyl) propanoic acid		x		
	uric acid		x		
	pantothenic acid		x		
	syringic acid			x	
	3-hydroxybutyric acid			x	
	phenylacetic acid			x	
	dimethylglycine			x	
	gluconic acid			x	
	pyroglutamic acid				x
	histidine				x
	n-acetyltryptophan				x
	alpha-aminobutyric acid / gamma-aminobutyric acid / 3-aminoisobutanoic acid / dimethylglycine				x
	indolelactic acid				x
	p-cresol sulfate				x
	n6-carboxymethyllysine				x

Table S26. (continued)

	variable	all visits	0M	6M	12M
metabolomics - platform for polar to semi-polar metabolites in positive mode	citrulline	x		x	x
	feature_mz_130.086	x		x	x
	kynurenic acid	x	x		
	5-aminolevulinic acid/4-hydroxyproline	x	x		
	dihydrouracil	x	x	x	
	n6,n6,n6-trimethylysine	x		x	
	adenosine	x	x		
	serotonin	x		x	x
	ornithine	x			x
	trimethylamine	x	x		
	ethanolamine		x		
	xanthurenic acid		x		
	deoxyguanosine		x		
	urocanic acid		x		
	sphingosine		x		
	n1-methyl-4-pyridone-3-carboxamide / nudifloramide			x	
	guanidineacetic acid			x	
	beta-guanidinopropionic acid			x	
	dodecanoylcarnitine			x	
	betaine			x	
	threonine/homoserine				x
	thymine				x
	quinaldic acid				x
	n-acetylcadaverine				x
	pyridoxal				x
	1-methyladenosine / N6-Methyladenosine / 2'-O-Methyladenosine				x

Table S26. (continued)

	variable	all visits	0M	6M	12M
metabolomics – platform for bile acids and fatty acids	eicosapentaenoic acid	x	x	x	x
	dihomo-alpha-linolenic acid	x			x
	taurolithocholic acid	x	x		
	glycochenodeoxycholic acid	x	x	x	x
	glycoursodeoxycholic acid	x		x	x
	glycocholic acid	x	x	x	x
	arachidonic acid	x	x		x
	docosahexaenoic acid	x	x	x	x
	deoxycholic acid	x		x	
	cholic acid	x	x		x
	taurocholic acid		x		
	chenodeoxycholic acid		x	x	
	taurodeoxycholic acid		x		
	lithocholic acid			x	
	hyocholic acid			x	
	tauroursodeoxycholic acid			x	x
	4,8,12,15,19-docosapentaenoic acid				x

*Fasta Maxquant:
tr.A0A3E4U7M2:
tr.A0A3E4U7M2.A0A3E4U7M2_9CLOT.L.fucose.isomerase.OS.Hungatella.hathewayi.OX.154046.GN.fucI.PE.3.SV.1.tr.WP_130789564.1.WP_130789564.1_NCBI.L.fucose.isomerase..Lachnoclostridium.pacaense..tr.A0A6P1Z546.A0A6P1Z546_9FIRM.L.fucose.isomerase.OS.Blautia.product
tr.A0A3E3EA02:
tr.A0A3E3EA02.A0A3E3EA02_9FIRM.Elongation.factor.Tu.OS.Erysipelatoclostridium.ramosum.OX.1547.GN.tuf.PE.3.SV.1.tr.A0A3E3AC32.A0A3E3AC32_9FIRM.Elongation.factor.Tu.OS.Erysipelatoclostridium.ramosum.OX.1547.GN.tuf.PE.3.SV.1
tr.A0A369M4T5:
tr.A0A369M4T5.A0A369M4T5_EGGLN.Serine.threonine.protein.kinase.OS.Eggerthella.lenta.OX.84112.GN.C1853_16350.PE.3.SV.1
tr.A0A5B3GKY1:
tr.A0A5B3GKY1.A0A5B3GKY1_ANAHA.Class.II.fructose.1.6.bisphosphate.aldolase.OS.Anaerostipes.hadrus.OX.649756.GN.fba.PE.4.SV.1.tr.WP_144365668.1.WP_144365668.1_NCBI.class.II.fructose.1.6.bisphosphate.aldolase..Lacrimispora.amygdalina.
tr.A0A151C8B3:
tr.A0A151C8B3.A0A151C8B3_BIFLN.ABC.transporter.substrate.binding.protein.OS.Bifidobacterium.longum.OX.216816.GN.APC1461_0390.PE.4.SV.1.tr.A0A2N0SXI9.A0A2N0SXI9_BIFLN.Solute.binding.protein.of.ABC.transporter.system.OS.Bifidobacterium.longum.OX.216816.GN.DP
tr.WP_119239543.1:
tr.WP_119239543.1.WP_119239543.1_NCBI.MULTISPECIES..chaperonin.GroEL..Clostridiales..tr.A0A564W1T1.A0A564W1T1_9FIRM.60.kDa.chaperonin.OS.Blautia.luti.OX.89014.GN.groL.PE.3.SV.1.tr.WP_173773732.1.WP_173773732.1_NCBI.chaperonin.GroEL..partial..Blautia.schink
tr.A0A0A1GRX3:
tr.A0A0A1GRX3.A0A0A1GRX3_BIFLN.DNA.directed.RNA.polymerase.subunit.beta.OS.Bifidobacterium.longum.OX.216816.GN.rpoB.PE.3.SV.1.tr.A0A1S2VY79.A0A1S2VY79_BIFLI.DNA.directed.RNA.polymerase.subunit.beta.OS.Bifidobacterium.longum.subsp..infantis.OX.1682.GN.rpoB
tr.E5XVX1:
tr.E5XVX1.E5XVX1_BIFLN.Uncharacterized.protein.OS.Bifidobacterium.longum.OX.216816.GN.HMPREF0177_00123.PE.4.SV.1.tr.A0A0A1GT43.A0A0A1GT43_BIFLN.DUF349.domain.containing.protein.OS.Bifidobacterium.longum.OX.216816.GN.APC1462_1477.PE.4.SV.1.tr.A0A0M4LRX8.A0A
tr.A0A2Y9B7L8:
tr.A0A2Y9B7L8.A0A2Y9B7L8_9FIRM.Nitrogen.fixation.NifU.like.protein.OS.Faecalicatena.orotica.OX.1544.GN.A8806_10120.PE.4.SV.1.tr.D4MZ07.D4MZ07_ANAHA.Fe.S.cluster.assembly.scaffold.protein.NifU.OS.Anaerostipes.hadrus.OX.649756.GN.nifU.PE.4.SV.1.tr.A5ZPB7.A5Z
tr.WP_008703889.1:
tr.WP_008703889.1.WP_008703889.1_NCBI.MULTISPECIES..anaerobic.carbon.monoxide.dehydrogenase.catalytic.subunit..Clostridiales.
tr.A0A1V8Q6G3:
tr.A0A1V8Q6G3.A0A1V8Q6G3_9BIFI.50S.ribosomal.protein.L27.OS.Bifidobacterium.dentium.OX.1689.GN.rpmA.PE.3.SV.1.tr.A0A1V8PSH8.A0A1V8PSH8_9BIFI.50S.ribosomal.protein.L27.OS.Bifidobacterium.catenulatum.OX.1686.GN.rpmA.PE.3.SV.1.tr.A0A087DLM1.A0A087DLM1_BIFAD.5
tr.D1PG79:

tr.D1PG79.D1PG79_9BACT.Fumarate.reductase.succinate.dehydrogenase.flavoprotein.subunit.OS.Prevotella.copri.DSM.18205.OX.537011.GN.sdhA.PE.4.SV.1
tr.WP_008705693.1:
tr.WP_008705693.1.WP_008705693.1_NCBI.MULTISPECIES..IMP.cyclohydrolase..Clostridiales..tr.WP_173726912.1.WP_173726912.1_NCBI.IMP.cyclohydrolase..Blautia.glucerasea..tr.WP_117854094.1.WP_117854094.1_NCBI.MULTISPECIES..IMP.cyclohydrolase..Clostridiales..tr.A
tr.A0A173R7M3:
tr.A0A173R7M3.A0A173R7M3_ANAHA.50S.ribosomal.protein.L5.OS.Anaerostipes.hadrus.OX.649756.GN.rplE.PE.3.SV.1
tr.WP_173773355.1:
tr.WP_173773355.1.WP_173773355.1_NCBI.MULTISPECIES..IMP.cyclohydrolase..Clostridiales..tr.WP_173718261.1.WP_173718261.1_NCBI.MULTISPECIES..IMP.cyclohydrolase..Clostridiales..tr.WP_148462774.1.WP_148462774.1_NCBI.MULTISPECIES..IMP.cyclohydrolase..Clostridia
tr.WP_008702995.1:
tr.WP_008702995.1.WP_008702995.1_NCBI.MULTISPECIES..sirohydrochlorin.cobaltochelatase..Clostridiales.
tr.WP_025577282.1:
tr.WP_025577282.1.WP_025577282.1_NCBI.MULTISPECIES..phosphoglycerate.kinase..Clostridiales..tr.WP_173766836.1.WP_173766836.1_NCBI.phosphoglycerate.kinase..Blautia.glucerasea..tr.A0A4Q1RGS8.A0A4Q1RGS8_9FIRM.Phosphoglycerate.kinase.OS.Blautia.faecicola.OX.25
tr.A0A1L8SSH0:
tr.A0A1L8SSH0.A0A1L8SSH0_9ENTE.Glutamate.dehydrogenase.OS.Enterococcus.devriesei.OX.319970.GN.RV00_GL003076.PE.3.SV.1
tr.A0A0H2PUX6:
tr.A0A0H2PUX6.A0A0H2PUX6_BIFBI.ABC.transporter.ATP.binding.protein.OS.Bifidobacterium.bifidum.OX.1681.GN.APS66_02165.PE.4.SV.1
tr.D4BR49:
tr.D4BR49.D4BR49_BIFBR.Receptor.family.ligand.binding.protein.OS.Bifidobacterium.breve.DSM.20213...JCM.1192.OX.518634.GN.BIFBRE_04584.PE.3.SV.1.tr.A0A4S5BB12.A0A4S5BB12_BIFLI.ABC.transporter.substrate.binding.protein.OS.Bifidobacterium.longum.subsp..infant
tr.A0A1Q2CBB9:
tr.A0A1Q2CBB9.A0A1Q2CBB9_ANAHA.30S.ribosomal.protein.S2.OS.Anaerostipes.hadrus.OX.649756.GN.rpsB.PE.3.SV.1.tr.D4N023.D4N023_ANAHA.30S.ribosomal.protein.S2.OS.Anaerostipes.hadrus.OX.649756.GN.rpsB.PE.3.SV.1
tr.A0A4S5BCM0:
tr.A0A4S5BCM0.A0A4S5BCM0_BIFLI.Carbamoyl.phosphate.synthase.large.chain.OS.Bifidobacterium.longum.subsp..infantis.OX.1682.GN.carB.PE.3.SV.1.tr.A0A2I1J0J4.A0A2I1J0J4_BIFLN.Carbamoyl.phosphate.synthase.large.chain.OS.Bifidobacterium.longum.OX.216816.GN.carB
tr.WP_070089094.1:
tr.WP_070089094.1.WP_070089094.1_NCBI.triose.phosphate.isomerase..Merdimonas.faecis.
tr.WP_025578671.1:
tr.WP_025578671.1.WP_025578671.1_NCBI.MULTISPECIES..pyruvate.ferredoxin..flavodoxin..oxidoreductase..Clostridiales.
tr.WP_028255879.1:
tr.WP_028255879.1.WP_028255879.1_NCBI.30S.ribosomal.protein.S2..Veillonella.magna.
tr.A0A3E3EF25:
tr.A0A3E3EF25.A0A3E3EF25_9FIRM.Hsp20.alpha.crystallin.family.protein.OS.Erysipelatoclostridium.ramosum.OX.1547.GN.DW242_03070.PE.3.SV.1
tr.WP_020993932.1:
tr.WP_020993932.1.WP_020993932.1_NCBI.MULTISPECIES..formate.C.acetyltransferase..Clostridiales.
tr.A6L7K3:
tr.A6L7K3.A6L7K3_BACV8.Major.outer.membrane.protein.OmpA.OS.Bacteroides.vulgatus..strain.ATCC.8482...DSM.1447...JCM.5826...NBRC.14291...NCTC.11154..OX.435590.GN.BVU_4065.PE.4.SV.1
tr.WP_025580226.1:
tr.WP_025580226.1.WP_025580226.1_NCBI.DNA.directed.RNA.polymerase.subunit.beta..Blautia.wexlerae.
tr.A0A329TVD2:
tr.A0A329TVD2.A0A329TVD2_9FIRM.Bifunctional.metallophosphatase.5.nucleotidase.OS.Faecalibacterium.prausnitzii.OX.853.GN.C4N26_12080.PE.3.SV.1
tr.A0A4V2X185:
tr.A0A4V2X185.A0A4V2X185_9BACT.SusC.RagA.family.TonB.linked.outer.membrane.protein.OS.Phocaeicola.dorei.OX.357276.GN.EL88_18780.PE.3.SV.1.tr.A6L1G3.A6L1G3_BACV8.Putative.outer.membrane.protein..probably.involved.in.nutrient.binding.OS.Bacteroides.vulgatus
tr.WP_147598065.1:
tr.WP_147598065.1.WP_147598065.1_NCBI.phosphoenolpyruvate.carboxykinase..ATP...Blautia.caecimuris.
tr.A0A3E3EA89:
tr.A0A3E3EA89.A0A3E3EA89_9FIRM.DNA.directed.RNA.polymerase.subunit.beta.OS.Erysipelatoclostridium.ramosum.OX.1547.GN.rpoB.PE.3.SV.1
tr.WP_022067817.1:
tr.WP_022067817.1.WP_022067817.1_NCBI.MULTISPECIES..rhamnulokinase..Clostridiales.
sp.P01701:
sp.P01701.LV151_HUMAN.Immunoglobulin.lambda.variable.1.51.OS.Homo.sapiens.OX.9606.GN.IGLV1.51.PE.1.SV.2
sp.P06702:
sp.P06702.S10A9_HUMAN.Protein.S100.A9.OS.Homo.sapiens.OX.9606.GN.S100A9.PE.1.SV.1

sp.A0A075B6H9:
sp.A0A075B6H9.LV469_HUMAN.Immunoglobulin.lambda.variable.4.69.OS.Homo.sapiens.OX.9606.GN.IGLV4.69.PE.1.SV.1
sp.P01024:
sp.P01024.CO3_HUMAN.Complement.C3.OS.Homo.sapiens.OX.9606.GN.C3.PE.1.SV.2
sp.P01703:
sp.P01703.LV140_HUMAN.Immunoglobulin.lambda.variable.1.40.OS.Homo.sapiens.OX.9606.GN.IGLV1.40.PE.1.SV.2
sp.A0A0C4DH72:
sp.A0A0C4DH72.KV106_HUMAN.Immunoglobulin.kappa.variable.1.6.OS.Homo.sapiens.OX.9606.GN.IGKV1.6.PE.3.SV.1.sp.P01599.KV117_HUMAN.Immunoglobulin.kappa.variable.1.17.OS.Homo.sapiens.OX.9606.GN.IGKV1.17.PE.1.SV.2
sp.A0A0A0MT36:
sp.A0A0A0MT36.KVD21_HUMAN.Immunoglobulin.kappa.variable.6D.21.OS.Homo.sapiens.OX.9606.GN.IGKV6D.21.PE.3.SV.1.sp.A0A0C4DH24.KV621_HUMAN.Immunoglobulin.kappa.variable.6.21.OS.Homo.sapiens.OX.9606.GN.IGKV6.21.PE.3.SV.1
sp.P02810:
sp.P02810.PRPC_HUMAN.Salivary.acidic.proline.rich.phosphoprotein.1.2.OS.Homo.sapiens.OX.9606.GN.PRH1.PE.1.SV.2
sp.P01834:
sp.P01834.IGKC_HUMAN.Immunoglobulin.kappa.constant.OS.Homo.sapiens.OX.9606.GN.IGKC.PE.1.SV.2
sp.Q86UP6:
sp.Q86UP6.CUZD1_HUMAN.CUB.and.zona.pellucida.like.domain.containing.protein.1.OS.Homo.sapiens.OX.9606.GN.CUZD1.PE.2.SV.1
sp.P15924:
sp.P15924.DESP_HUMAN.Desmoplakin.OS.Homo.sapiens.OX.9606.GN.DSP.PE.1.SV.3
sp.Q9UGM3:
sp.Q9UGM3.DMBT1_HUMAN.Deleted.in.malignant.brain.tumors.1.protein.OS.Homo.sapiens.OX.9606.GN.DMBT1.PE.1.SV.2
sp.P68133:
sp.P68133.ACTS_HUMAN.Actin..alpha.skeletal.muscle.OS.Homo.sapiens.OX.9606.GN.ACTA1.PE.1.SV.1
sp.Q5D862:
sp.Q5D862.FILA2_HUMAN.Filaggrin.2.OS.Homo.sapiens.OX.9606.GN.FLG2.PE.1.SV.1
sp.Q86YZ3:
sp.Q86YZ3.HORN_HUMAN.Hornerin.OS.Homo.sapiens.OX.9606.GN.HRNR.PE.1.SV.2
sp.P01009:
sp.P01009.A1AT_HUMAN.Alpha.1.antitrypsin.OS.Homo.sapiens.OX.9606.GN.SERPINA1.PE.1.SV.3
sp.P09923:
sp.P09923.PPBI_HUMAN.Intestinal.type.alkaline.phosphatase.OS.Homo.sapiens.OX.9606.GN.ALPI.PE.1.SV.2
sp.Q9Y6R7:
sp.Q9Y6R7.FCGBP_HUMAN.IgGFc.binding.protein.OS.Homo.sapiens.OX.9606.GN.FCGBP.PE.1.SV.3
sp.P15085:
sp.P15085.CBPA1_HUMAN.Carboxypeptidase.A1.OS.Homo.sapiens.OX.9606.GN.CPA1.PE.1.SV.2
sp.P01764:
sp.P01764.HV323_HUMAN.Immunoglobulin.heavy.variable.3.23.OS.Homo.sapiens.OX.9606.GN.IGHV3.23.PE.1.SV.2.sp.P0DP03.HV335_HUMAN.Immunoglobulin.heavy.variable.3.30.5.OS.Homo.sapiens.OX.9606.GN.IGHV3.30.5.PE.3.SV.1.sp.P01768.HV330_HUMAN.Immunoglobulin.heavy.var
sp.P68133:
sp.P68133.ACTS_HUMAN.Actin..alpha.skeletal.muscle.OS.Homo.sapiens.OX.9606.GN.ACTA1.PE.1.SV.1
sp.P04745:
sp.P04745.AMY1_HUMAN.Alpha.amylase.1.OS.Homo.sapiens.OX.9606.GN.AMY1A.PE.1.SV.2
sp.P09093:
sp.P09093.CEL3A_HUMAN.Chymotrypsin.like.elastase.family.member.3A.OS.Homo.sapiens.OX.9606.GN.CELA3A.PE.1.SV.3
sp.Q8WWU7:
sp.Q8WWU7.ITLN2_HUMAN.Intelectin.2.OS.Homo.sapiens.OX.9606.GN.ITLN2.PE.2.SV.1
sp.P21796:
sp.P21796.VDAC1_HUMAN.Voltage.dependent.anion.selective.channel.protein.1.OS.Homo.sapiens.OX.9606.GN.VDAC1.PE.1.SV.2
sp.A0A0J9YXX1:
sp.A0A0J9YXX1.HV5X1_HUMAN.Immunoglobulin.heavy.variable.5.10.1.OS.Homo.sapiens.OX.9606.GN.IGHV5.10.1.PE.3.SV.1.sp.A0A0C4DH38.HV551_HUMAN.Immunoglobulin.heavy.variable.5.51.OS.Homo.sapiens.OX.9606.GN.IGHV5.51.PE.3.SV.1

**Abbreviations immune data: CXCL5: C-X-C motif chemokine 5; 4E-BP1: Eukaryotic translation initiation factor 4E-binding protein 1; MMP-10: Matrix metalloproteinase-10; IL18 : Interleukin-18; EN-RAGE: Protein S100-A12; IL-15RA: Interleukin-15 receptor subunit alpha; IL8 : Interleukin-8; CX3CL1: Fractalkine; IL-1-alpha: Interleukin-1 alpha; IL-20RA: Interleukin-20 receptor subunit alpha; MMP-1: Matrix metalloproteinase-1; LIF-R: Leukemia inhibitory factor receptor; Flt3L: Fms-related tyrosine kinase 3 ligand; CASP-8: Caspase-8; CCL4: C-C motif chemokine 4; PD-L1: Programmed cell death 1 ligand 1; MCP-4: Monocyte chemotactic protein 4; TNFSF14: Tumor necrosis factor ligand superfamily member 14; TNFRSF9: Tumor necrosis factor receptor superfamily member 9; LIF: Leukemia inhibitory factor; uPA: Urokinase-type plasminogen activator; IL7: Interleukin-7; OSM: Oncostatin-M; IL-12B: Interleukin-12 subunit beta.

Application on a public data set

As an additional example, we apply our multi-omics approach on a subset of 11 insuline resistant and 13 insuline sensitive individuals from a study of Sailani *et al* (Sailani *et al.*, 2020, data set: https://doi.org/10.6084/m9.figshare.12376508.v1), for whom human cytokine, metabolomics, nasal microbiome and proteomics data were publicly available that were measured during the periods January 2015 – April 2015 (T1), May 2015 – August 2015 (T2) and September – December 2015 (T3). In case samples for the same subject were measured multiple times during the same period, we included the first measurement. We applied our approach to classify the subjects into insuline resistant and insuline sensitive. For each view and each of the two approaches, we fitted parameters mtry and ntee (Tables S27-S28). As the data set was (approximately) balanced, there was not need to fit the decision threshold and we could use the default of 0.5. The results in Tables S29-S30 show that also for this data set approach 2 outperforms approach 1.

Table S27. Application on a public data set. Fitted parameters mtry and ntree for each model in approach 1, together with their AUC on the training and test set.

model	mtry	ntree	AUC train	AUC test
**cytokine data**	4	2000	0.477	0.633
**metabolomics**	30	500	0.595	0.688
**nasal microbiome**	7	1000	0.465	0.652
**proteomics**	21	500	0.459	0.649

Table S28. Application on a public data set. Fitted parameters mtry and ntree for each model in approach 2, together with their AUC on the training and test set.

model	mtry	ntree	AUC train	AUC test
**cytokine data – T1**	11	1000	0.573	0.816
**cytokine data – T2**	5	500	0.415	0.571
**cytokine data – T3**	8	500	0.529	0.631
**metabolomics – T1**	28	2000	0.593	0.788
**metabolomics – T2**	24	1000	0.709	0.792
**metabolomics – T3**	25	500	0.573	0.734
**nasal microbiome – T1**	4	500	0.469	0.581
**nasal microbiome – T2**	4	500	0.534	0.659
**nasal microbiome – T3**	8	1500	0.492	0.757
**proteomics – T1**	19	500	0.638	0.861
**proteomics – T2**	17	2000	0.527	0.663
**proteomics – T3**	13	500	0.508	0.632

Table S29. Application on a public data set. Forward selection of best combination of views for approach 1. AUC-test: mean AUC over the 5 test sets. Red = removed because lowers performance. Blue = kept because increases performance. Bold = best classifier.

Combination	AUC test
**metabolomics (best individual classifier)**	**0.688**
metabolomics + cytokine data	0.652 (< 0.688)
metabolomics + nasal microbiome	0.532 (< 0.688)
metabolomics + proteomics	0.671 (< 0.688)

Table S30. Application on a public data set. Forward selection of best combination of views for approach 2. AUC-test: mean AUC over the 5 test sets. Red = removed because lowers performance. Blue = kept because increases performance. Bold = best classifier.

Combination	AUC test
proteomics – T1 (best individual classifier)	0.861
**proteomics – T1 + cytokine data – T1**	**0.913**
proteomics – T1 + cytokine data – T1 + cytokine data – T2	0.832 (< 0.913)
proteomics – T1 + cytokine data – T1 + cytokine data – T3	0.778 (< 0.913)
proteomics – T1 + cytokine data – T1 + metabolomics – T1	0.901 (< 0.913)
proteomics – T1 + cytokine data – T1 + metabolomics – T2	0.913*
proteomics – T1 + cytokine data – T1 + metabolomics – T3	0.890 (< 0.913)
proteomics – T1 + cytokine data – T1 + nasal microbiome T1	0.859 (< 0.913)
proteomics – T1 + cytokine data – T1 + nasal microbiome T2	0.810 (< 0.913)
proteomics – T1 + cytokine data – T1 + nasal microbiome T3	0.729 (< 0.913)
proteomics – T1 + cytokine data – T1 + proteomics – T2	0.834 (< 0.913)
proteomics – T1 + cytokine data – T1 + proteomics – T3	0.837 (< 0.913)

* but proteomics – T1 + cytokine data – T1 has a better trade-off between sensitivity and specificity (proteomics – T1 + cytokine data: sensitivity = 0.753, specificity = 0.700; proteomics – T1 + cytokine data – T1 + metabolomics – T2: sensitivity = 0.670, specificity = 0.733)

Aitchison, J. The Statistical Analysis of Compositional Data. Chapman and Hall, London; 1986.

Bateman, A., *et al.* UniProt: the universal protein knowledgebase in 2021. *Nucleic Acids Research* 2021;49(D1):D480-D489.

Chatchatee, P., *et al.* Tolerance development in cow's milk-allergic infants receiving amino acid-based formula: A randomized controlled trial. *J Allergy Clin Immunol* 2022;149(2):650-658 e655.

Cox, J. and Mann, M. MaxQuant enables high peptide identification rates, individualized p.p.b.-range mass accuracies and proteome-wide protein quantification. *Nat Biotechnol* 2008;26(12):1367-1372.

Cox, J., *et al.* Andromeda: A Peptide Search Engine Integrated into the MaxQuant Environment. *J Proteome Res* 2011;10(4):1794-1805.

Hendrickx, D.M., *et al.* Assessment of infant outgrowth of cow's milk allergy in relation to the faecal microbiome and metaproteome. *Sci Rep* 2023;13(1):12029.

Hendrickx, D.M., *et al.* Identification of potential inflammation markers for outgrowth of cow's milk allergy. *bioRxiv* 2024; 2024.05.24.595813; doi: https://doi.org/10.1101/2024.05.24.595813

Hosseinkhani, F., *et al.* Towards Standards for Human Fecal Sample Preparation in Targeted and Untargeted LC-HRMS Studies. *Metabolites* 2021;11(6).

Lahti, L. and Shetty, S. microbiome R package. 2012-2019.

Martino, C., *et al.* A Novel Sparse Compositional Technique Reveals Microbial Perturbations. *Msystems* 2019;4(1).
Quast, C., *et al.* The SILVA ribosomal RNA gene database project: improved data processing and web-based tools. *Nucleic Acids Res* 2013;41(Database issue):D590-596.
R Core Team. R: A Language and Environment for Statistical Computing. 2022.
Sailani, M.R. *et al*, Deep longitudinal multiomics profiling reveals two biological seasonal patterns in California, Nat Commun, 2020, 11, 4933.
Sailani, Reza (2020). Multi_Omics_Seasonal.RData. figshare. Dataset. https://doi.org/10.6084/m9.figshare.12376508.v1
